# Comparing the predictions of CT-based subject-specific finite element models of human metastatic vertebrae with digital volume correlation measurements

**DOI:** 10.1007/s10237-025-01950-x

**Published:** 2025-04-19

**Authors:** Chiara Garavelli, Alessandra Aldieri, Marco Palanca, Enrico Dall’Ara, Marco Viceconti

**Affiliations:** 1 Department of Industrial Engineering, Alma Mater Studiorum - University of Bologna, Bologna, Italy; 2 Medical Technology Lab, IRCCS Istituto Ortopedico Rizzoli, Bologna, Italy; 3 PolitoBIOMedLab, Department of Mechanical and Aerospace Engineering, Politecnico di Torino, Corso Duca Degli Abruzzi, 24, Turin, Italy; 4 Division of Clinical Medicine, School of Medicine and Population Health, University of Sheffield, Sheffield, UK; 5 Insigneo Institute, University of Sheffield, Sheffield, UK

**Keywords:** Vertebra, Fracture prediction, Finite element model, Digital volume correlation, Validation

## Abstract

Several conditions can increase the incidence of vertebral fragility fractures, including metastatic bone disease. Computational tools could help clinicians estimate the risk of vertebral fracture in these patients; however, comparison with in vitro data is mandatory before using them in clinical practice. Nine spine segments were tested under compression and imaged with micro-computed tomography (µCT). The displacement field was calculated for each vertebra using a global digital volume correlation (DVC) approach. Subject-specific homogenised finite element models of each vertebra were built from µCT images, applying experimentally matched boundary conditions at the endplates. Numerical and experimental displacements, reaction forces, and locations showing higher strain concentrations were eventually compared. Additionally, given that µCT cannot be performed in clinical settings, the outcomes of a µCT-based model were also compared to those of a model built from clinical CT scans of the same specimen. Good agreement between DVC and µCT-based FE displacements was found, both for healthy (*R*^2^ = 0.69 ÷ 0.83, RMSE = 3 ÷ 22%, max error < 45 μm) and metastatic (*R*^2^ = 0.64 ÷ 0.93, RMSE = 5 ÷ 18%, max error < 54 μm) vertebrae. Strong correlations were found between µCT-based and clinical CT-based FE model outcomes (*R*^2^ = 0.99, RMSE < 1.3%, max difference = 6 μm). Furthermore, the models qualitatively identified the most deformed regions identified with the experiments. In conclusion, the combination of experimental full-field technique and in-silico modelling enabled the development of a promising pipeline to validate bone strength predictors in the elastic range. Further improvements are needed to analyse vertebral post-yield behaviour better.

**Supplementary Information:**

The online version contains supplementary material available at 10.1007/s10237-025-01950-x.

## Introduction

In 2022, the World Health Organisation (WHO) reported an estimated 20 million new cancer cases worldwide and over 35 million new cancer cases are predicted in 2050 (Bray et al. 2024). Among them, one-third presents signs of spinal metastasis (Van Den Brande et al. 2022), which has been demonstrated to increase the vertebral risk of fracture (Kaneko et al. 2004) and decrease the quality of life (Alexandru 2012). In the light of this, constant monitoring of vertebral stability in pathological patients is crucial, aiming to prevent fracture occurrence.

In the last decades, the finite element (FE) methodology has been used to predict the strength of healthy or osteoporotic vertebrae (Crawford et al. 2003; Imai et al. 2006; Buckley et al. 2007; Dall’Ara et al. 2012; Molinari and Falcinelli 2022). CT-based subject-specific FE models have recently been used to assess the resistance of vertebrae affected by metastatic osteolytic lesions (Costa et al. 2019), providing a better risk stratification than traditional densitometric measurements (Stadelmann et al. 2020). Typically, FE model predictions are validated by comparing them to point-wise local strain measurements acquired on the cortex using strain gauges (Alkalay and Harrigan 2016), global mechanical properties measurements (Stadelmann et al. 2020) and, more recently, digital image correlation measurements of displacements and strains on the vertebral external surface (Gustafson et al. 2017; Baleani et al. 2023; Garavelli et al. 2022). However, the accurate prediction of the local internal deformations within the whole vertebral body, within the lesion and around it, would be desirable to investigate the mechanical response of the vertebra before fracture.

From this perspective, digital volume correlation (DVC) represents a valuable tool for comparing numerical predictions with experimental outcomes, as it provides full-field displacement measurements over the whole bone volume (Roberts et al. 2014; Grassi and Isaksson 2015; Dall’Ara and Tozzi 2022). This contactless technique requires two stacks of images of the same bone in undeformed and deformed configurations as input. Computed tomography (CT) images with different resolution are the primary source of input to apply the DVC technique in the study of bone behaviour: from micro-CT (µCT) scans (Tozzi et al. 2016) to clinical CT scans (Peña Fernández et al. 2020). After methodological optimisation, experimental uncertainties on the displacement field can be lowered below the voxel size (Palanca et al. 2015). The DVC approach has been widely used to validate computational models of different bones (Dall’Ara and Tozzi 2022) and enables the assignment of experimentally matched boundary conditions (BCs) to the models (Chen et al. 2017; Kusins et al. 2020). Vertebral bodies FE model outcomes have already been compared to DVC measurements, accounting for the bone tissue microstructure (i.e. micro-FE models (Costa et al. 2017; Palanca et al. 2022)) or based on heterogeneous mapping of the bone mineral density from clinical images (Jackman et al. 2016; Hussein et al. 2018) providing promising results. Nevertheless, these studies focussed on porcine or human healthy vertebrae.

The aim of this work was thus to develop FE models for both healthy and metastatic human vertebral bodies and to pointwise compare their displacement predictions across the whole vertebral body with DVC-derived experimental measurements. Eventually, in light of the proposed strain-based criteria to predict fracture initiation in bone (Molinari and Falcinelli 2022), the ability of the models to correctly identify the regions with higher strain localization in the elastic regime was also assessed.

## Materials and methods

### Mechanical testing

Experimental tests were approved by the ethical committees of the University of Bologna (n. 17,325, 08/02/2019) and the University of Sheffield (n. 031782, 22/06/2020). The tests were conducted on nine thoracolumbar cadaveric segments (spine levels from T5 to L3) obtained from an ethically approved donation program (Anatomy Gifts Registry, Inc.) and previously used in Palanca et al. 2021, 2023; Cavazzoni et al. 2023.

**Fig. 1:**
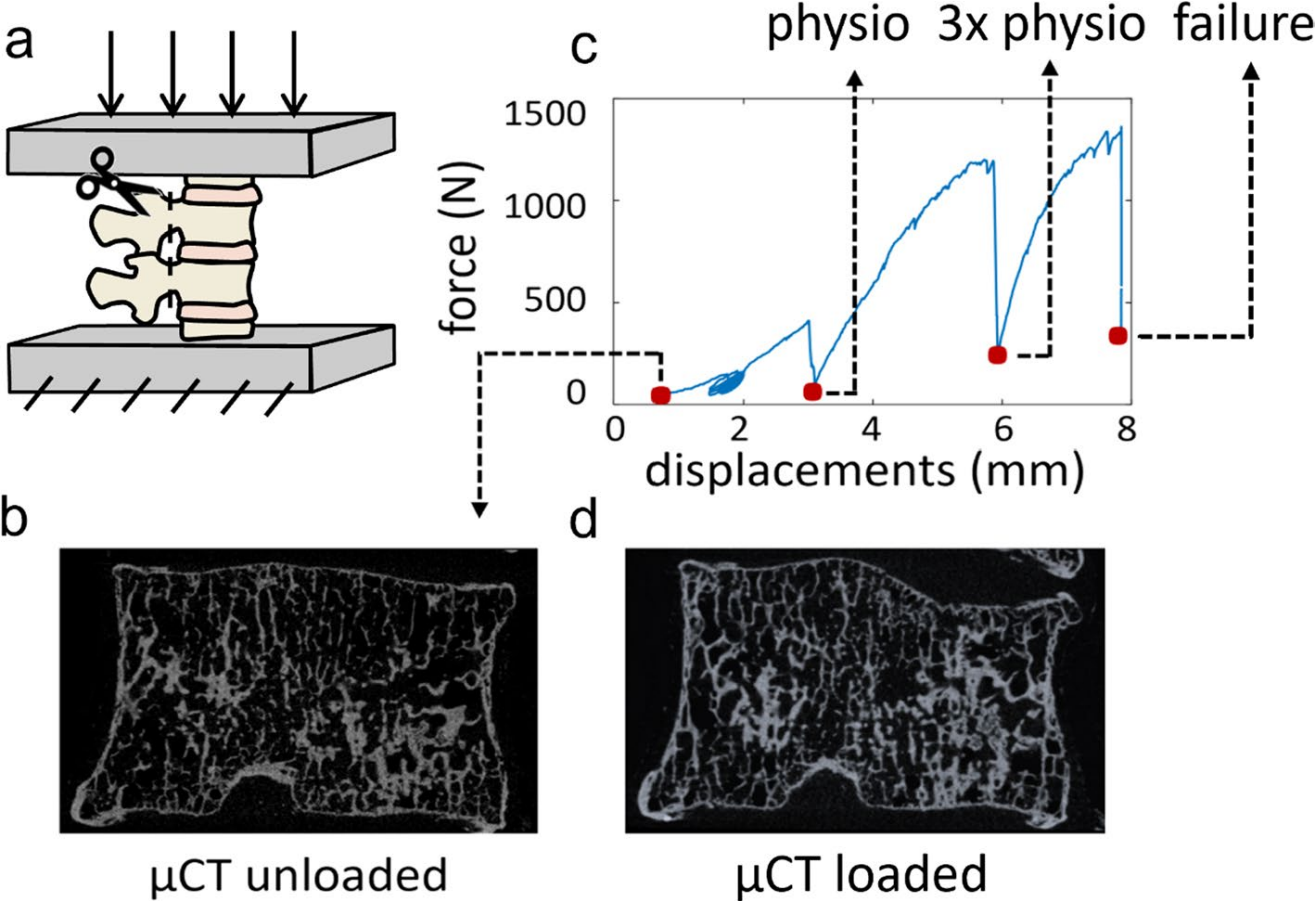
Experimental test. The specimens were composed of four vertebrae without the posterior elements. Only the middle vertebrae were analysed in the study, while the most cranial and caudal ones were used for applying the load (**a**). μCT scans were acquired in the unloaded configuration (**b**), before starting the compression test (**c**), and then in the loaded condition (**d**), after each load step (red dots) until failure (in the picture, as a representative example, the scan after failure has been reported). In the FE analyses only one load-step was considered: the highest with at least 75% of the analysed vertebral bone volume showing DVC principal strains below thresholds for failure (Bayraktar et al. 2004)

Each specimen was composed of four vertebrae: the two vertebrae at the extremities were embedded in polymethylmethacrylate (PMMA) bases and used to apply the load, while the two in the middle, used for the comparison with DVC measurements, were composed of one vertebra showing signs of metastatic lesions and one radiologically healthy (control). The specific type of metastasis (i.e. lytic, blastic or mixed) was defined for each metastatic vertebra radiologically (Cavazzoni et al. 2025), based on the identification of focal regions with low bone mineral density (lytic lesion) or regions with the bone mineral density much higher than the surrounding trabecular bone (blastic lesion). Mixed lesions contained a mix of the lytic and blastic tissues. During the preparation of the specimens, the posterior elements were removed (Fig. 1a) to fit a custom jig. Each specimen was fixed in the jig and placed within a µCT scanner (VivaCT80, Scanco, Switzerland). The unloaded (Fig. 1b) control and metastatic vertebral bodies were scanned with the following parameters: isotropic voxel size 39 μm, current 114 mA, voltage 70kVp, integration time 300 ms, power 8W). The performed mechanical tests are described in greater detail in Palanca et al. 2023. Briefly, the compressive load of the first loading step was selected to induce strains typical of the physiological regime (around 2000–3000 microstrain) on the cortical shell of the healthy vertebra (Palanca et al. 2021). The load was then increased by three times (3xphysio) for the second step, and then it was increased until one of the two vertebrae failed (Fig. 1c). After each load step, the specimen was left to relax for 20 min and then µCT scanned (Fig. 1d).

**Fig. 2:**
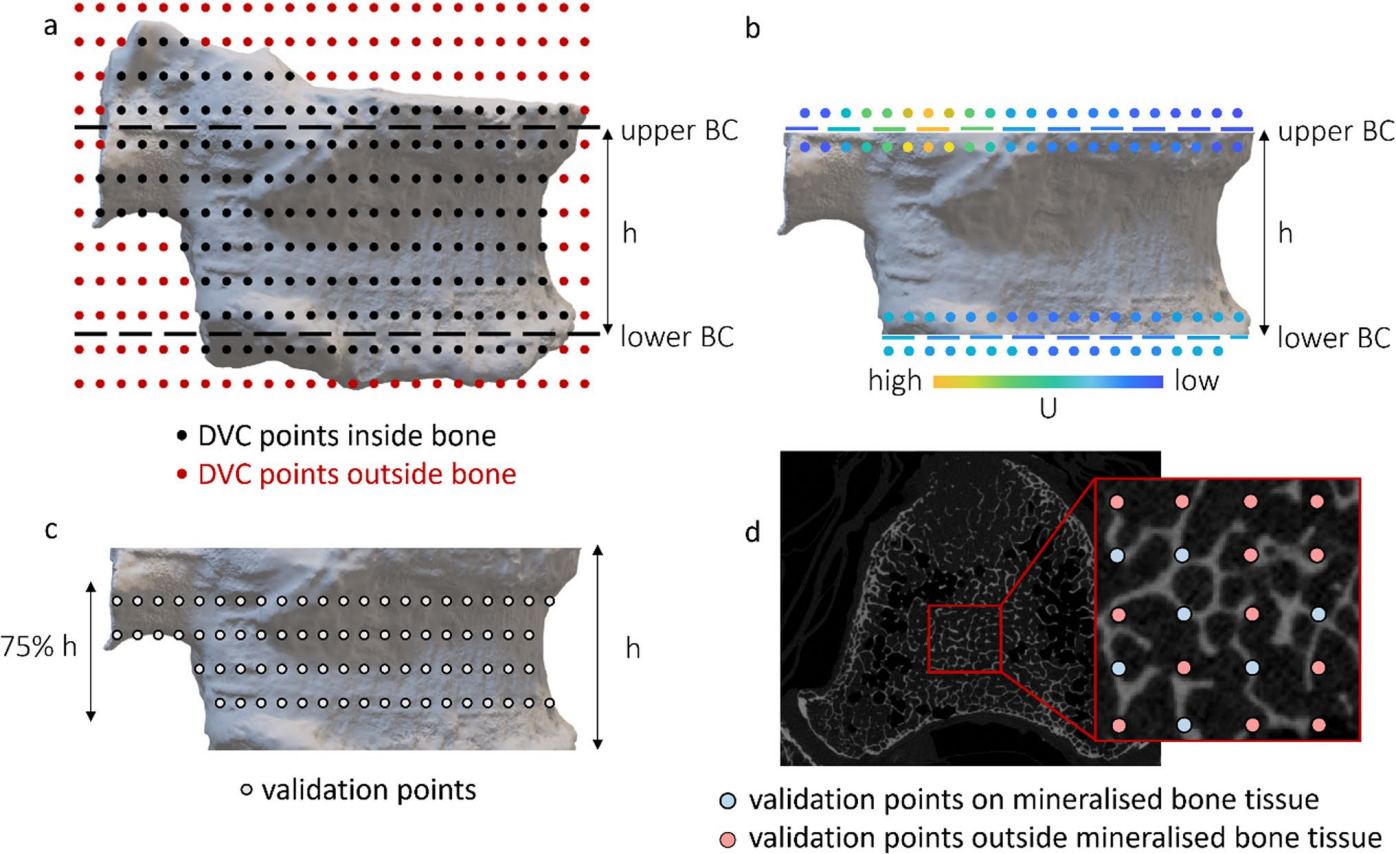
Implementation of the point-to-point FE/DVC comparison. **a** The DVC approach measures displacement and strains at the nodes of a hexahedral grid with element size equal to the nodal spacing. Among all the points (red + black dots), only those falling inside the vertebral body are used (black dots). The BCs location has been defined as a mesh node plane between the two DVC grid slices falling inside the bone and closest to the endplates. **b** The DVC displacements (U, coloured dots) have been interpolated onto the previously defined mesh nodes (corresponding to the dotted coloured lines). **c** The DVC points used for the validation are those within 75% of the distance between the BC lines. Point-to-point comparison between the results of the experiments and models was carried out identifying the coordinates of the DVC point, and calculating the FE displacements in those coordinated using the elements shape functions. **d** Additionally, the validation was performed considering only the points localised inside mineral bone tissue (blue dots)

DVC analysis, implemented through the BoneDVC algorithm (Dall’Ara et al. 2017), was performed at each loading step by inputting the µCT images of the unloaded and loaded specimens. The original µCT images were cropped to include only one vertebral body for each analysis. The displacement field was obtained by applying an elastic registration, which can minimise the difference between the deformed and registered images (i.e. the undeformed images after applying the target displacement field) (Dall’Ara et al. 2017). The displacement field was then differentiated into a strain field. DVC measurements (i.e. cartesian displacements and principal strains) were carried out using a measurement spatial resolution (nodal spacing, i.e. the distance between two nodes of the DVC registration grid, Fig. 2a) equal to 1.95 mm. Further technical details about the DVC technique can be found in Palanca et al. 2023. Experimental uncertainties for displacement and strain measurements were computed applying the BoneDVC algorithm to two subsequent scans of the unloaded specimens (zero-strain condition) and have been reported in a previous study (Cavazzoni et al. 2023). Displacement uncertainties were computed as the standard deviations of the measurements across DVC grid nodes for the three Cartesian components of the displacement, while strain uncertainties were calculated by considering the standard deviation of the average of the absolute values of the six DVC-derived strain components (SDER). Considering the nodal spacing chosen for this study, the displacement uncertainties were expected to be between 1 and 30 μm. In contrast, SDER was expected to be between 90 and 1030 με, depending on the considered specimen.

### Finite element analysis

The FE modelling pipeline was applied only to the vertebrae not showing any sign of damage and the load step (physio, 3xphysio, or failure) was chosen so that less than 25% of the vertebral body volume exceeded the bone failure strain values (1.04% compression, 0.73% tension) (Bayraktar et al. 2004). Table 1s in the Supplementary Material 1 provides an overview of the modelled and analysed vertebrae. μCT-based FE models were created for sixteen vertebrae: ten healthy vertebrae and six metastatic vertebrae, of which three had lytic lesions and three had mixed lesions.

For one of the healthy vertebrae, FE models were generated also starting from the available clinical CT images. The outputs of these models were compared with the respective μCT based FE models.

In analogy with the experimental analysis, where the DVC measurements were carried out on single vertebral bodies, FE models of each vertebra were also created independently. This approach enabled the matching of the boundary conditions between the FE models and the DVC analyses at regions just caudally than the cranial endplate and cranially than the caudal endplate. This procedure (i.e. match of the boundary conditions within the vertebral body) did not require the modelling of the intervertebral disc, for which geometrical and microstructural properties are not visible in the µCT images.

Because the models were generated from the unloaded µCT scans of each single vertebra, the FE models and DVC data shared the same reference system, enabling the reduction in the errors associated with the process of registering the model with the experimental results. The outer contour of each vertebra was segmented from the µCT images using a semi-automatic segmentation procedure (Mimics v25, Materialise NV, Leuven, Belgium) and a 10-node tetrahedral structural solid mesh was later created (ICEM CFD v19.3, ANSYS Inc.), with an edge length equal to 1 mm (Costa et al. 2019).

The material properties were assigned analogously for the healthy and pathological vertebrae: µCT grey levels were calibrated to obtain equivalent tissue mineral density values using a hydroxyapatite phantom. Then, Bonemat software (Taddei et al. 2007), developed at Rizzoli Orthopaedic Institute, was used to integrate the voxel density over each mesh element. The density (*ρ*_APP_ [g/cm^3^])—elasticity (E [MPa]) relationship was derived from the literature (Morgan et al. 2003; Schileo et al. 2008) (Eq. 1):

1 $$E=4730{\rho }_{\text{APP}}^{1.56}$$

Boundary conditions were retrieved from the DVC data and imposed to the FE model to replicate the experimental loading condition. For each vertebra, the two DVC grid slices included in the vertebral body and closest to the cranial (upperBC) and caudal (lowerBC) endplates were selected (dashed red lines in Fig. 2b). The FE nodes at the extremity of the model were assigned displacements interpolated from the upperBC and lowerBC by using a trilinear interpolation algorithm (Matlab® v2023, MathWorks, Natick, Massachusetts, US) (Fig. 2b). Then, the endplate regions cranially than upperBC and caudally than lowerBC regions were removed (Fig. 2b).

The numerical reaction forces were computed along the axial direction at lowerBC nodes.

The simulations were run in an FE analysis environment (Mechanical APDL v19.3, ANSYS Inc.) to predict the cartesian displacements, the principal strains and the reaction forces. The simulations were performed on a standalone computer (parallel distributed memory over six cores with 64 GB of RAM (Intel(R) Xeon(R) E-2276G CPU 3.80 GHz) and required approximately 1 h.

### Comparing µCT-based FE models versus DVC

To compare the displacements prediction of the FE model against experimental data, the FE nodal displacements were interpolated at the location of the DVC nodes using the shape functions of the FE elements. Two sets of nodes were considered for the validation: (1) all DVC nodes inside the vertebral body; (2) only the DVC nodes inside the bone mineralised tissue (i.e. trabecula, cortical shell, blastic tissue; Fig. 2d). The point-to-point validation was always performed on the central 75% of the modelled vertebra, to be sufficiently far from the nodes used to apply the boundary conditions (Fig. 2c).

### Comparison metrics

The agreement between FE and DVC displacements was tested using linear regression analysis, and the intercept, slope, coefficient of determination (*R*^2^), root mean squared error (RMSE), and percentage RMSE (RMSE%, obtained normalising the RMSE by the higher experimental displacement) were reported. *R*^2^ coefficients of control and metastatic FE models were transformed using Fisher’s r to z transformation, and z tests were performed to assess whether their differences were statistically significant. Among the tested specimens, those showing a statistically significant (*p* < 0.05) correlation between the displacement nodal predictive error (i.e. the difference between FE and DVC displacements) and the corresponding experimental uncertainty values were excluded from the analysis. This prevented that the errors associated with the FE model predictions were affected by the uncertainties of the experimental measurements.

Moreover, maximum and minimum principal strains obtained from the FE models were compared to those extracted in the experiments to identify if the models correctly localised the regions with high deformations. Additionally, DVC and predicted strain distributions were statistically compared through a nonparametric two‐sample Wilcoxon rank test (significance level = 5%) for each analysed vertebra.

Eventually, the agreement between the axial reaction forces predicted by the FE analysis and those measured in the experiments was assessed, and the *R*^2^, RMSE% and maximum error reported.

### Comparing µCT-based versus clinical-CT-based FE models

For one specimen, the same tetrahedral mesh already mapped onto the µCT image was roto-translated to be aligned to a clinical CT image (tube current 200 mA; voltage 120 kVp; voxel dimension 0.24 × 0.24 × 1 mm) of the same specimen acquired in a previous study (Palanca et al. 2021) using a procedure accurately described elsewhere (Garavelli et al. 2022) and densitometrically calibrated using the European Spine Phantom (ESP). The elastic properties of each element were calculated as previously explained from its equivalent bone mineral density value (Morgan et al. 2003). After the CT-based material properties assignment, the FE model was brought back to the µCT reference system registered to the DVC data. Eventually, the model was solved by applying the same boundary condition previously described for the µCT-based model. A point-to-point comparison of the displacements predicted by the two models was performed, and the agreement between the two was quantified in terms of RMSE, RMSE%, *R*^2^ and maximum difference. Moreover, the percentage difference between the two predicted reaction forces was computed.

### Assessment of the error propagation on the strains

Since bone failure is commonly based on maximum strain, and considering that DVC measures displacements, the possibility of propagating the error computed on displacements to a strain error was analysed on one healthy vertebra, following two approaches. On the one hand, a first estimation of the strain error was performed considering the predictive error on displacements (i.e. the difference between FE and DVC displacements) divided by the NS. This enabled the comparison between the obtained order of magnitude of the predictive strain error and the order of magnitude of the experimental strain uncertainty (Cavazzoni et al. 2023). On the other hand, a point-to-point estimation of the strain predictive error was also obtained. More in detail, the point-to-point difference between FE and DVC displacements at all DVC point locations was used to solve a simulation on the hexahedral DVC mesh in the FE environment (Mechanical APDL, Ansys Inc.), enabling to obtain, by derivation, the errors on the strain field. Eventually, the DVC strain uncertainties were subtracted from the obtained strain field at each DVC point to isolate only the portion of the strain error field due to the model.

## Results

Among the analysed vertebrae, one control vertebra was excluded from the validation because it fitted the exclusion criterion (Supplementary Materials 1, Fig. 1s).

### Comparing µCT-based FE models versus DVC

**Fig. 3:**
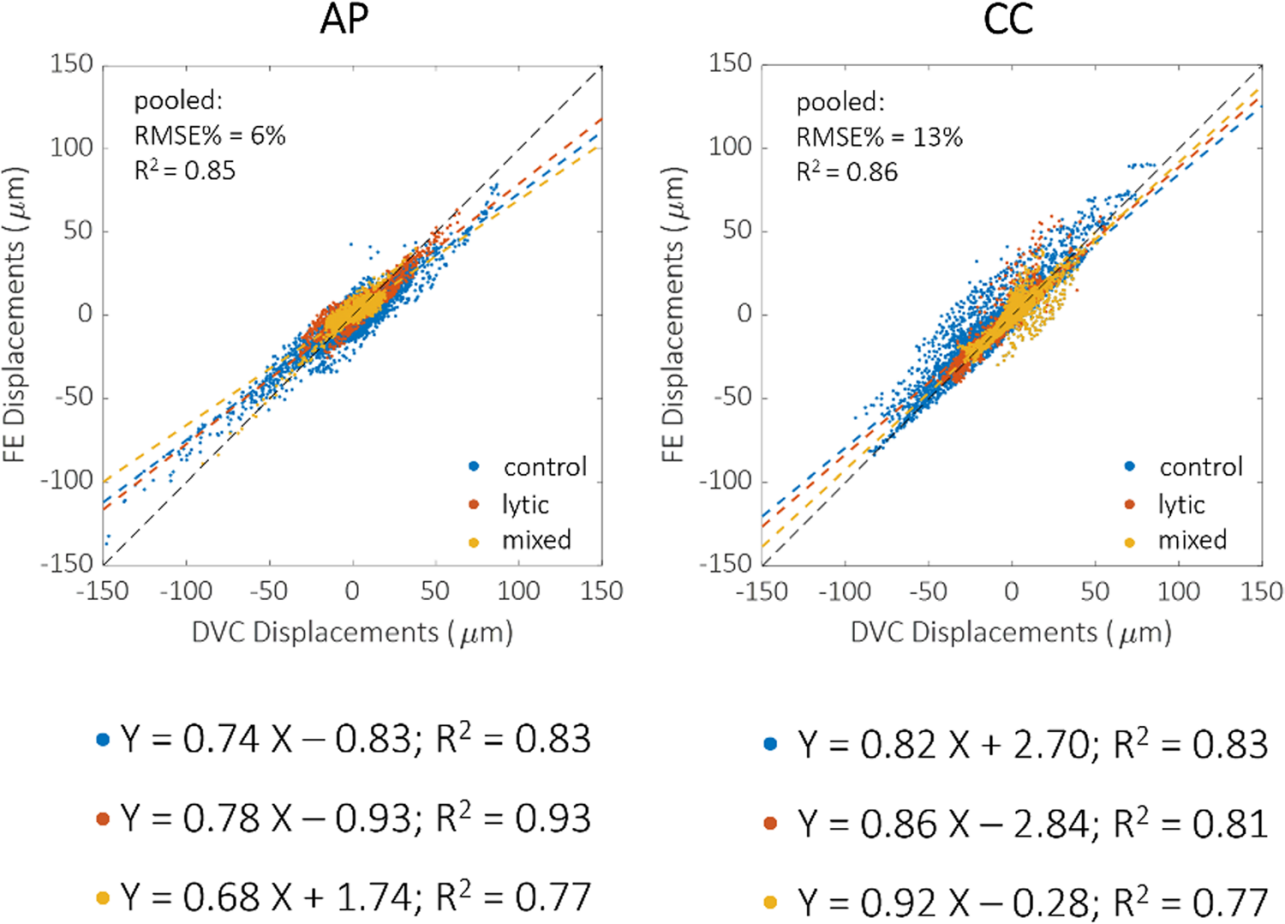
Comparison between DVC (horizontal axis) and FE (vertical axis) displacements on anteroposterior (AP) and craniocaudal (CC) directions, respectively. Control vertebrae are reported in blue, lytic in orange and mixed in yellow. Regression lines and *R*^2^ are also reported for each type of vertebra, while RMSE% is reported for the pooled groups

**Fig. 4:**
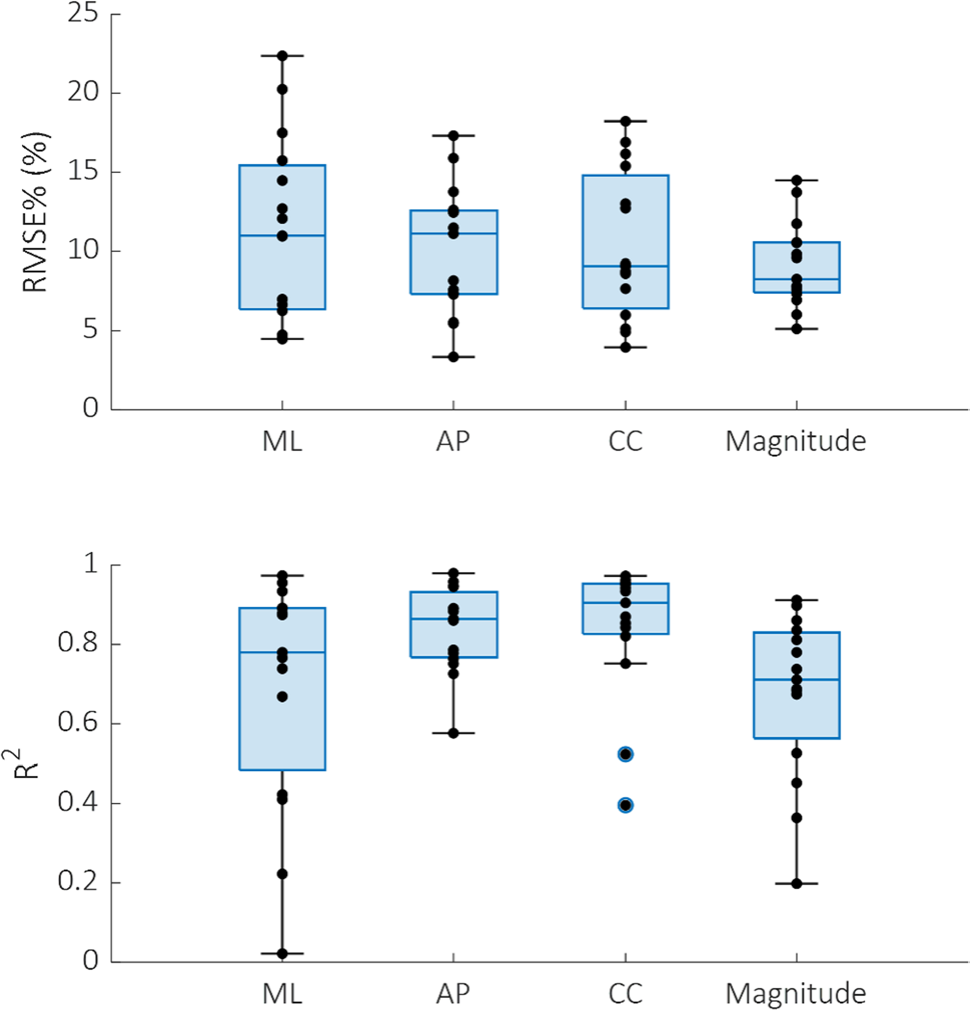
Boxplots reporting displacements RMSE% and *R*^2^ for all the vertebrae analysed. These metrics have been computed on all the DVC nodes (both those within and outside the mineralized bone tissue). Mediolateral (ML), anteroposterior (AP) and craniocaudal (CC) directions and the overall magnitude are reported

The FE model slightly underestimated the experimental displacements in all directions and for all the vertebra types (Fig. 3). However, no specific dependence of the linear regression parameters on the vertebra type was found. The strongest correlation for the displacements along the anterior–posterior direction was found for the lytic vertebrae (*R*^2^ = 0.93, *p* < 0.0001). In contrast, the strongest correlation for the displacements along the craniocaudal direction was found for the healthy ones (*R*^2^ = 0.83, *p* < 0.0001). The correlation coefficients were significantly different, except for the control and the lytic groups in the craniocaudal direction (z-test p-value = 0.053). The RMSE% for the control vertebrae was between 3 and 22%, and the maximum error was lower than 45 μm (1.15 µCT voxel). In contrast, the RMSE% was between 5 and 18% for lesioned vertebrae, and the maximum error was lower than 54 μm (1.38 µCT voxel). For pooled data, the RMSE% was 6% along the anterior–posterior direction and 13% along cranio-caudal direction. The displacements along the mediolateral direction were lower than the voxel size for most of the points. The correlation indexes of each vertebra are also reported in the boxplots in Fig. 4, with no statistically significant differences identified among the directions. In the Supplementary Material 1 comparison between DVC and predicted displacements are shown separately for each vertebra, together with the spatial distributions of the displacement predictions error.

**Fig. 5:**
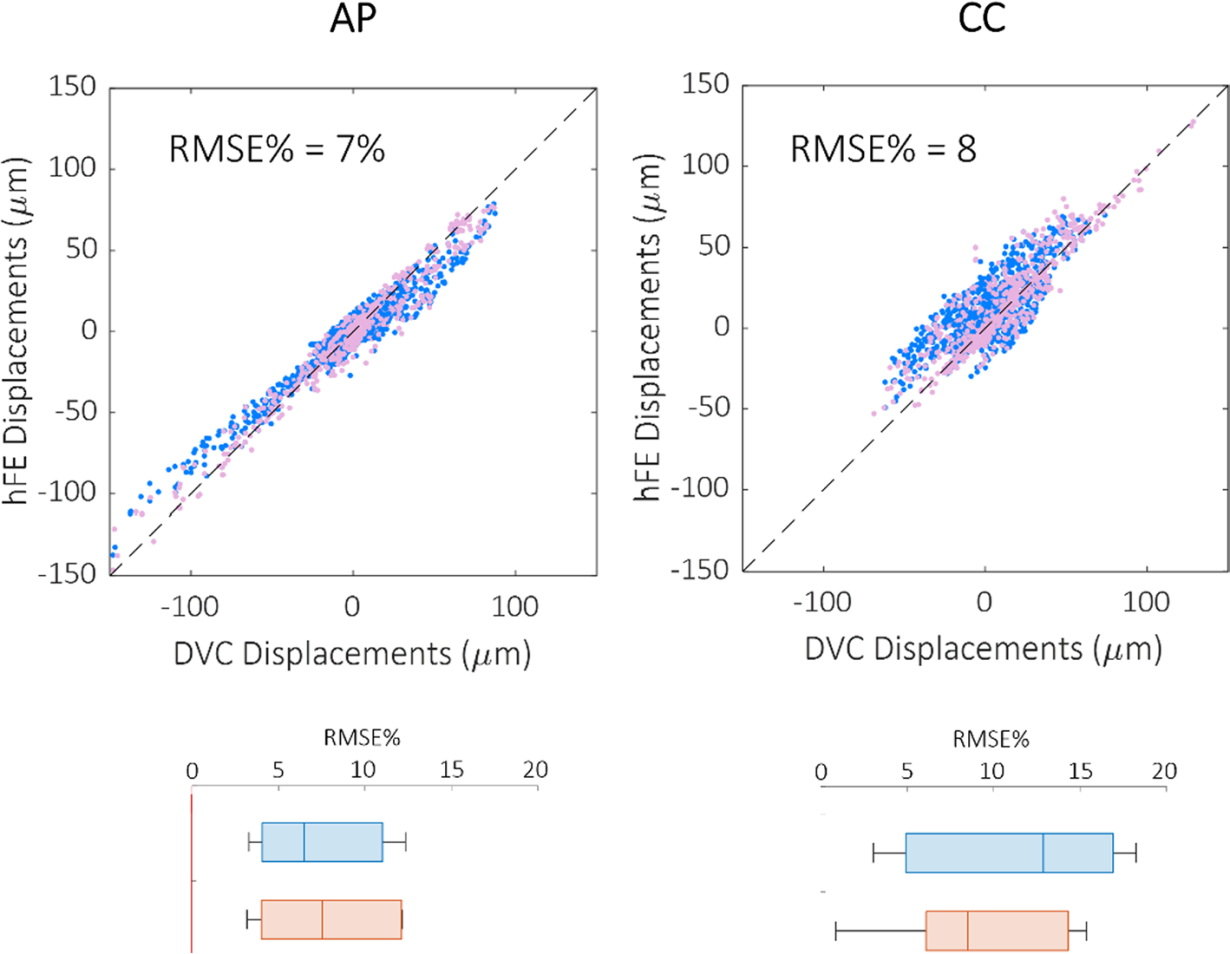
Scatter plots reporting the comparison between DVC (horizontal axis) and FE (vertical axis) displacements on mineralised bone DVC points only, along the anteroposterior (AP) and craniocaudal (CC) directions, respectively. Below each direction, the relative changes in RMSE% due to the restriction of the analysed points are reported (light blue before, pink after the restriction)

Narrowing the analysis to the DVC nodes within the mineralised bone tissue, an evident even if not statistically significant (*p* > 0.05) decrease in the RMSE% was observed for the displacements along the craniocaudal direction*.* In contrast, no considerable differences were observed for the displacements along the anteroposterior direction (Fig. 5). Results along the ML direction were not considered because the displacements along that direction were smaller than the voxel size.

**Fig. 6:**
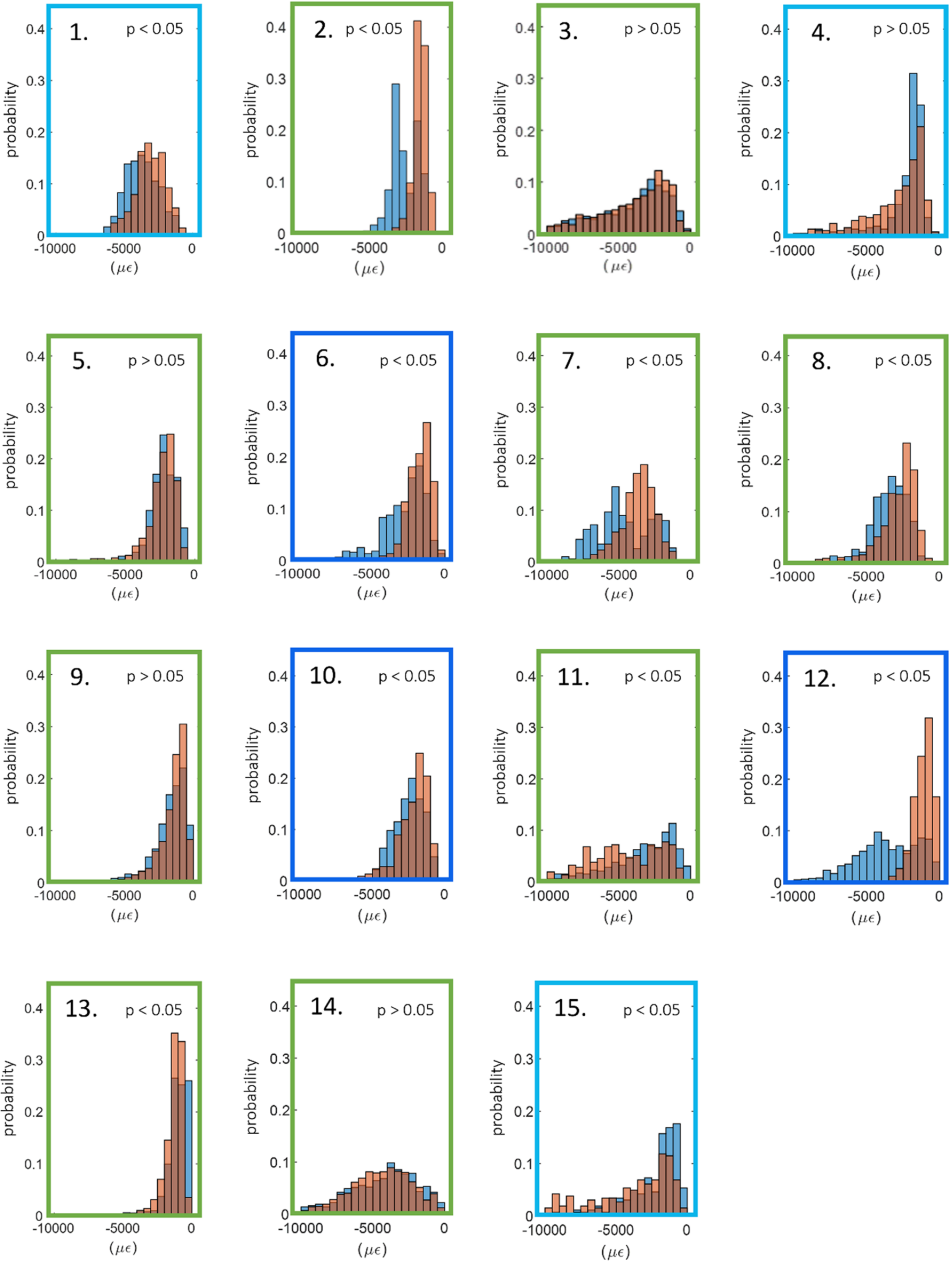
Histograms with the probability distributions of DVC (blue) and computational (orange) minimum principal strains for all the specimen analysed. The colour of the box surrounding each histogram plot refers to the vertebral type, i.e. control vertebrae are highlighted in green, lytic vertebrae in light blue and blastic vertebrae in dark blue

In Fig. 6 the minimum principal strain distributions obtained from the DVC approach or predicted by the FE models have been compared. While in several cases a good overlap between the histograms was observed, statistically significant differences (*p* < 0.05) were identified in ten cases (all the three mixed vertebrae, two of the three lytic vertebrae and five of the nine control vertebrae).

**Fig. 7:**
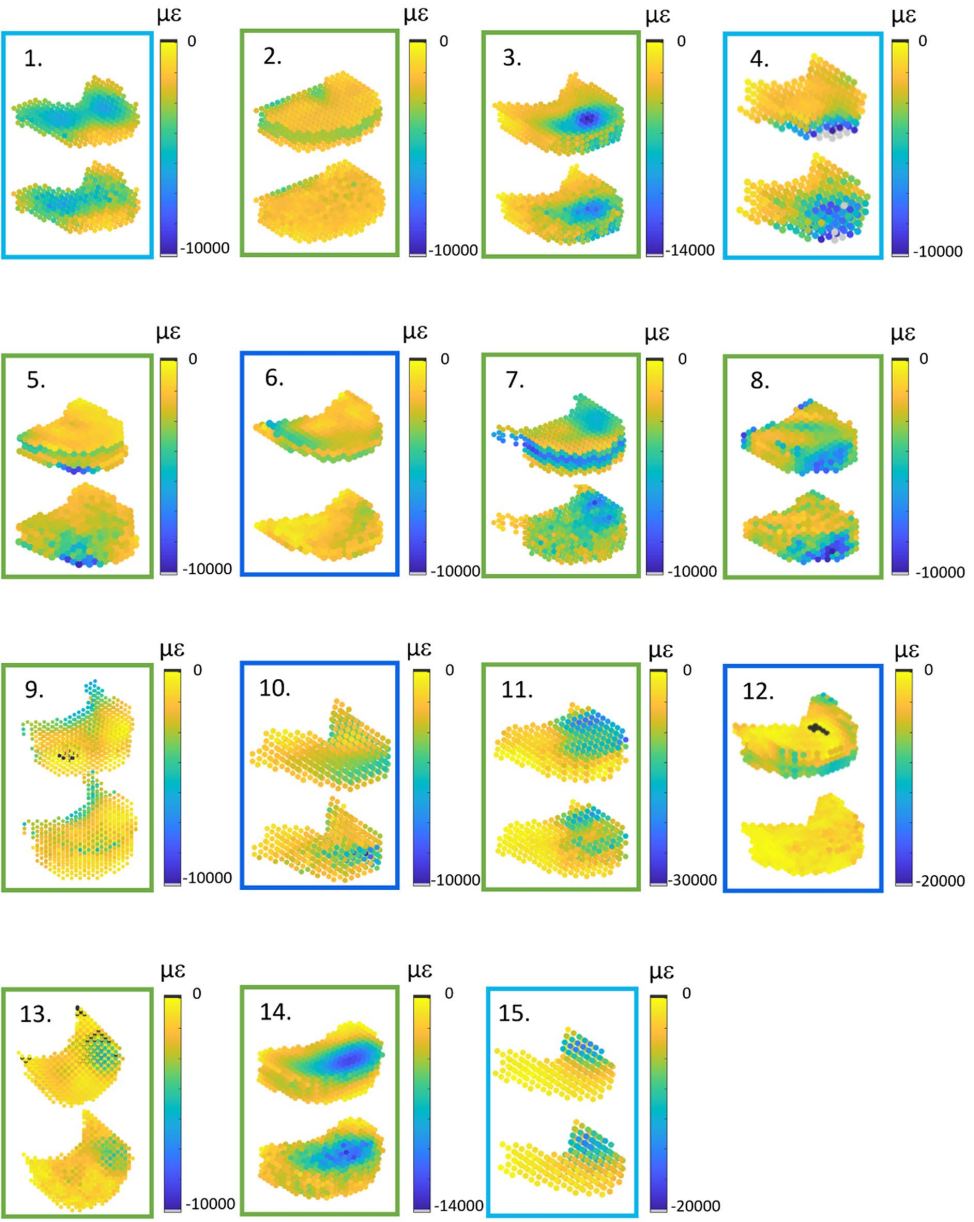
Spatial distributions of experimental (top) and computational (bottom) eminimum principal strains for all the analysed vertebral bodies. The colour of the box surrounding each histogram plot refers to the vertebral type, i.e. control vertebrae are highlighted in green, lytic vertebrae in light blue and blastic vertebrae in dark blue

**Fig. 8:**
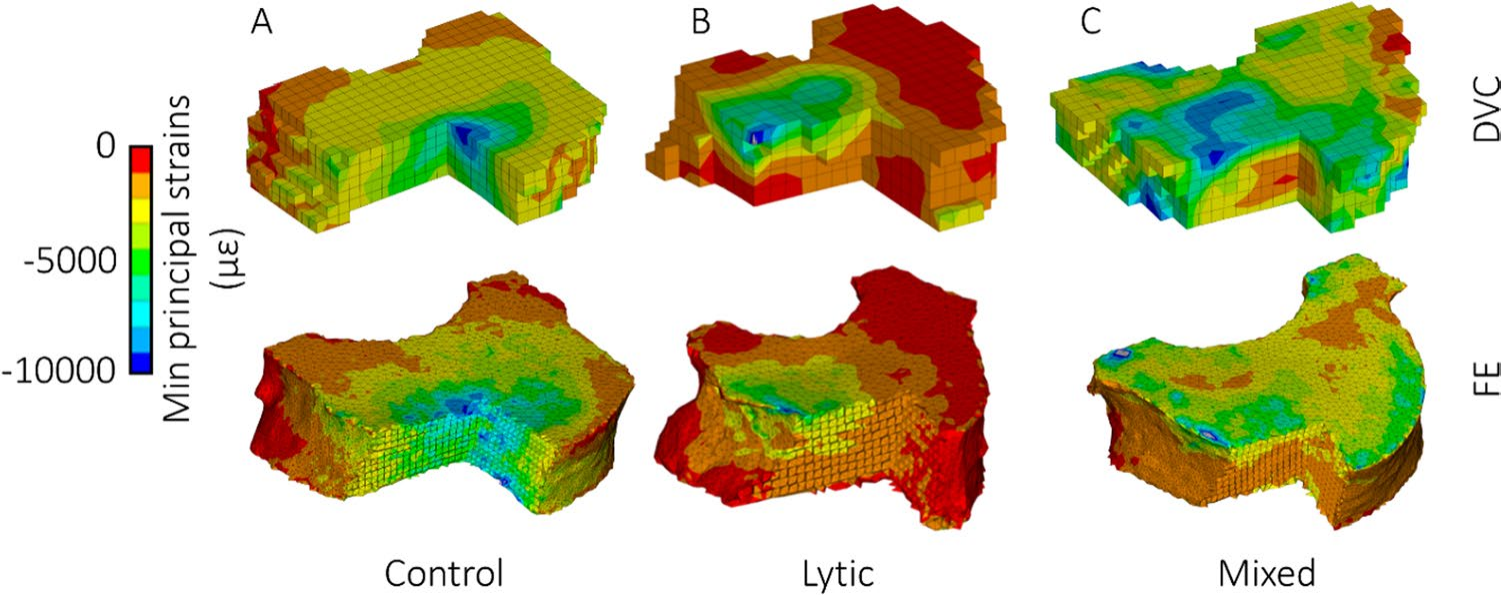
Qualitative comparison between DVC and FE minimum principal strains. Three different vertebrae are reported as examples for the control (**A**), lytic (**B**) and mixed (**C**) vertebrae; for each one, the upper contour plot refers to the DVC hexahedral grid, while the lower refers to the FE tetrahedral mesh. To show the quality of the comparison also within the vertebral body, all the vertebrae are reported in the figure after sectioning a portion of them

A qualitative comparison of DVC and FE strains is also reported in Fig. 7 for all the analysed vertebrae. In most cases, the regions with high strains observed from the DVC measurements were well identified by the FE models for control vertebrae and vertebrae with lytic lesions. On the other hand, FE models of the vertebrae with mixed metastases showed a lower ability to predict the regions with high strains (vertebrae 6, 10 and 12). Figure 8 displays with greater details the minimum principal strain distribution of one control, one lytic and one mixed metastatic vertebra as an example. Comparison between DVC and predicted maximum principal strains are provided in the Supplementary Material 1.

**Fig. 9:**
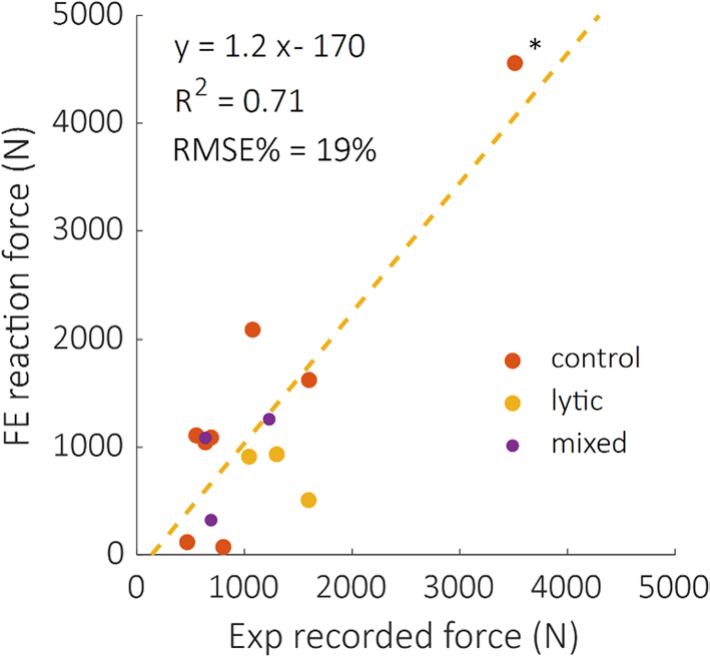
Comparison between computational and experimentally recorded reaction forces, divided according to their healthy status. The regression line (yellow dotted line) of the pooled group is also reported. *R*^2^ decreases to 0.2 if the high force value (*) is not considered

Lastly, a fair correlation was found between the axial reaction forces predicted by the FE analysis and those measured during the experimental tests, with *R*^2^ = 0.71, RMSE = 19% and maximum error equal to 1368 N (Fig. 9).

### Comparing µCT-based versus clinical-CT-based FE models

**Fig. 10:**
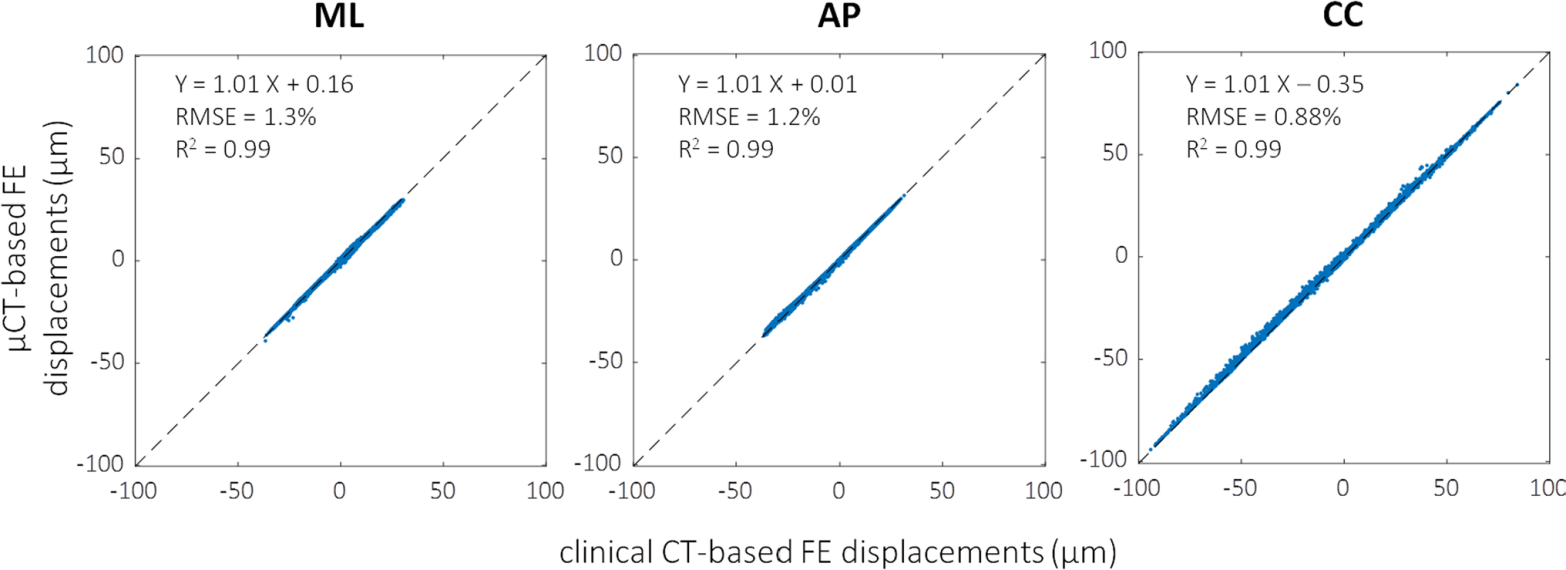
Correlations between displacement fields predicted by clinical-CT- (horizontal axis) and µCT-based (vertical axis) models. Regression lines, *R*^2^ and RMSE% are reported

A strong agreement between the displacements field obtained from the µCT-based and the clinical-CT-based models was found, with RMSE lower than 0.75 µm (RMSE% < 1.3%) and *R*^2^ higher than 0.99. The maximum difference among all the nodes and considering the three cartesian directions was 6 µm (Fig. 10). In terms of predicted reaction forces, the difference between μCT-based and clinical-CT-based models settled at around 1%, specifically 2088 N in the former case and 2119 N in the latter.

### Assessment of the error propagation on the strains

**Fig. 11:**
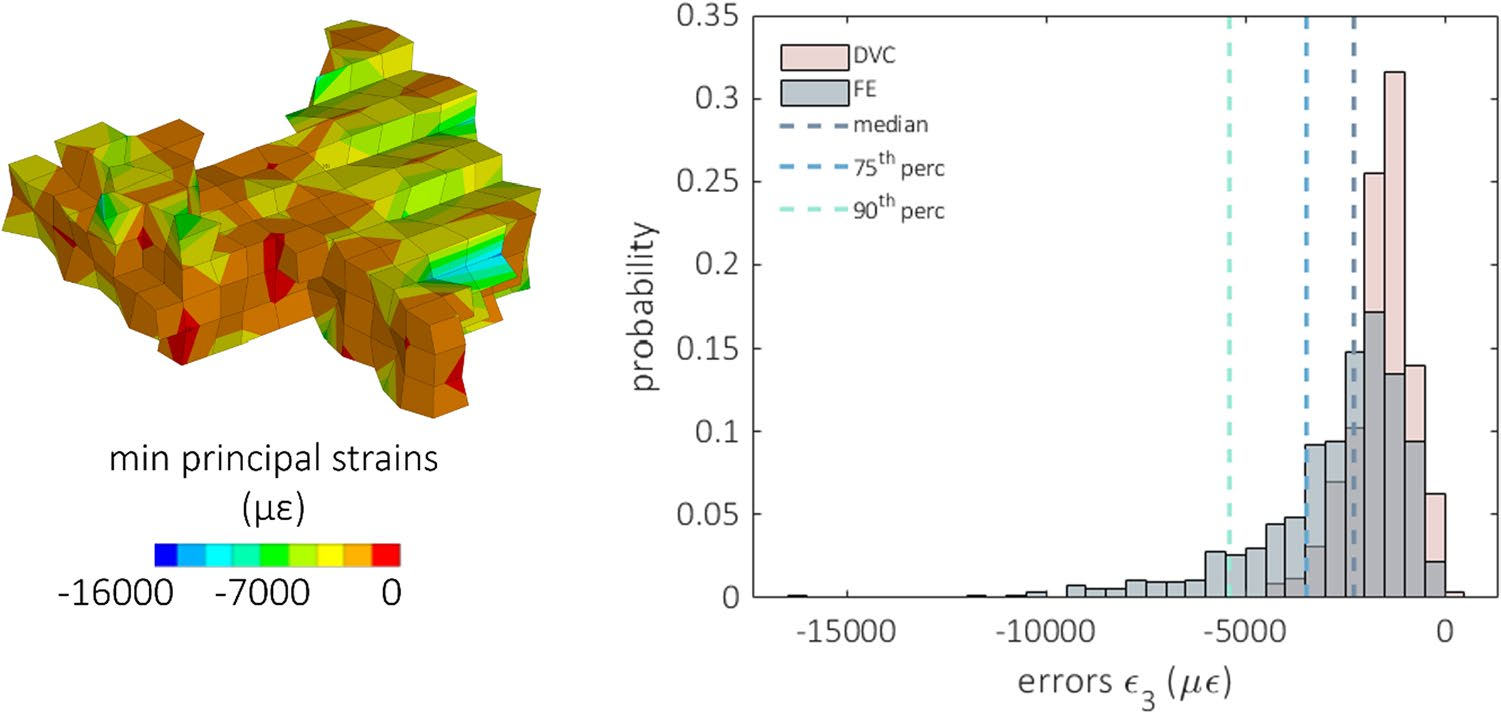
On the left, the contour plot of the minimum principal strain is reported due to the derivation of the displacement error. The strain field is obtained by superimposing the displacement error at each node of the hexahedral DVC grid. To show the comparison also within the vertebral body, the vertebra is reported in the figure after sectioning the anterior right portion of it, exposing the inner part. The histograms comparing the predictive error (grey) on the minimum principal strain and the corresponding experimental uncertainties (pink) computed on the same points are compared on the right. Median, 75th and 90th percentile of the predictive errors are also reported

According to the first approach adopted to estimate the propagation of the predictive error on the strains, the estimation of the magnitude of the strain error computed on one single vertebra resulted in the range 1000–25000 µε (taking into account minimum e maximum predictive displacement errors), while the experimental zero-strain uncertainties were in the order of 1000 µε, at worst an order of magnitude less. Instead, following the second approach, when the error on the displacements was superimposed to the DVC grid nodes on the same vertebra and derived through the FE method, the median of the error on the strain turned out to be 3000 µε with a range of 0–16000 µε (Fig. 11).

## Discussion

Compared to state-of-the-art DVC measurements, this study assessed the accuracy of subject-specific FE models of radiologically healthy and metastatic vertebral bodies with experimentally matched BCs in predicting internal displacements and strains.

Good agreement between DVC and FE displacement fields, both for healthy (*R*^2^ = 0.69 ÷ 0.83, RMSE% = 3 ÷ 22%, max error < 45 μm) and metastatic (*R*^2^ = 0.64 ÷ 0.93, RMSE% = 5 ÷ 18%, max error < 54 μm) vertebrae was found. The moderately better results obtained for FE displacements predictions on healthy and lytic vertebrae could depend on the adopted density-elasticity relationship for modelling vertebral bone mechanical response, which had been developed for healthy bone, and might fail in correctly capturing a blastic/mixed lesion real mechanical behaviour. Furthermore, when minimum principal strains were considered, no statistically significant differences were identified between DVC and predicted strain distributions for 5 vertebrae. Furthermore, the models could qualitatively identify the regions that experimentally showed highest strain for healthy and lytic vertebrae. However, the propagation of displacement error to strain errors yielded average errors of around three thousand microstrains. While these values are high, the magnitude is comparable to the accuracy of the DVC method estimated with zero-strain studies (around 1000 µε) (Cavazzoni et al. 2023). Thus, concerning the FE models accuracy in predicting strain, we can only conclude that this study represents a comparison exercise between models and experiments rather than a proper validation study for the models, since the experimental errors are not negligible compared to the computational errors. This hampers the possibility to quantitatively compare strains which would be the biomechanically critical variable for predicting the local bone failure. Since DVC accuracy is linked to the spatial resolution of the µCT images, doing a meaningful validation on strain predictions would require nanoCT images with a resolution of few micrometres. However, this would limit the analysis to a much smaller field of view, preventing the comparison at the whole vertebra scale.

Satisfactory but not excellent agreement was found in the reaction forces (*R*^2^ = 0.71, RMSE% = 19%), corroborating the density-elasticity law employed. These values are slightly lower than those reported in literature for the single vertebral specimen (*R*^2^ = 0.78–0.95 (Imai et al. 2006; Gustafson et al. 2017; Stadelmann et al. 2020)), but higher than the correlations obtained with DVC applied to a multivertebral set up (*R*^2^ = 0.09) (Hussein et al. 2018). Nevertheless, as the good agreement is mainly due to one specimen with high predicted and measured force, further analyses should be done to increase the range of failure loads and generalise the findings. Moreover, further research should be done to understand if the constitutive model (density to elasticity relationship) should be adjusted to consider the presence of the metastatic lesions.

The presented results in terms of FE displacements prediction of DVC measurements agreed with those reported by Palanca et al. 2022. There, µFE models of porcine vertebrae with and without mechanically induced lesions were developed and experimental displacements were applied as BCs, supporting the implementation performed in this study, to be eventually compared against DVC (RMSE% = 1.01–14.51, *R*^2^ = 0.65–1.00, slope = 0.77–1.19). The lower correlation found in this study compared to Palanca et al. 2022 could be explained by considering that this study tested human vertebrae, some of them with lytic or mixed metastasis, and this aspect increased the difficulty in the modelling of the material properties. Jackman et al. 2016 compared the prediction of FE models of human vertebrae against DVC measurements with an experimental set-up similar to that used in this study. Their findings for the compression tests with experimentally matched boundary conditions showed a median error in displacements in the range of 20–80% (average = 49%). Computing the same value for the presented data, the resulting range is 13–109% (average = 39%), highlighting a comparable fitting of the experimental data. DVC techniques have also been used to validate FE models of the scapular bone, analysing both the displacements (Kusins et al. 2019) and the strain fields (Kusins et al. 2020), respectively, with point-to-point and averaged comparisons. This study achieved moderately less accurate displacement field predictions (*R*^2^ = 0.40–0.98, slope = 0.43–1.32 in this study versus *R*^2^ = 0.79–1.00, slope = 0.87–1.09 in Kusins et al. 2019), motivated by the presence of the lesions and different anatomical sites. Also, the correct identification of the regions which experimentally showed the highest strain concentration by the FE model was confirmed in Kusins et al. 2020.

The excellent correlation found between µCT-based and clinical CT-based FE model predictions is an important outcome, as CT-based FE models could be created from clinical images. We acknowledge that the validation of such models against ex vivo experiments based on the biomechanical variables of interest, i.e. strains or failure load, is a required first step to enhance model credibility, but it should be followed by a clinical validation step too (Aldieri et al. 2023). Yet, the obtained agreement lays the foundations to the possible future adoption of clinical CT for subject-specific models-based predictions.

It is necessary to highlight some of the limitations of this work. Firstly, the comparison was made only for vertebrae that did not show visible signs of failure at the time of the loaded scan. This is because the developed models were linear elastic and, therefore, intrinsically unable to predict deformations beyond the elastic regime correctly. Nevertheless, we cannot exclude the presence of potential local damage in the trabecular network in region with high experimentally measured strains, which could have affected the interpretation of the obtained outcomes. Further improvements in the computational modelling pipeline would be needed to analyse the post-yield behaviour of the vertebra quantitatively through the inclusion of plasticity and damage, for instance. Secondly, the endplate regions were excluded from the models, preventing the application of more physiological boundary conditions. However, endplate regions are known to be of major interest for the vertebrae failure mechanisms (Palanca et al. 2023). Although it is fair to reproduce controlled experiments computationally in a validation study like the here presented one, this criticality could be overcome by extending the DVC measurements to the adjacent intervertebral discs (Tavana et al. 2020), reducing the higher uncertainties at the border where less information is available from the images, and interpolating the boundary conditions in the model at the endplates. However, DVC measurements on soft and hard tissues together are challenging and need further development (Dall’Ara and Tozzi 2022). Similarly, also the removal of the posterior elements to fit the specimens into the experimental setup prevented the possibility to observe a physiological mechanical response of the vertebrae, which can be considered reasonable in the context of a validation study like the here presented one. Thirdly, the same constitutive laws were employed for all the analysed vertebrae. While this choice may have introduced some inaccuracies, it was aligned with the findings of other authors who observed similarities in the mechanical properties of trabecular bone with and without lesions (Nazarian et al. 2008; Stadelmann et al. 2020). This assumption holds when metastatic lesions can be characterised as low-density bone tissue, as in the case of lytic lesions, whilst it might not be accurate enough for blastic/mixed lesions, which have been shown to exhibit lower mineralisation and marginally inferior mechanical properties than healthy bone even if they have been shown to present similar local indentation properties (Stadelmann et al. 2020). If evident signs of blastic metastatic lesions are detected, other constitutive models may be used to better reproduce the real mechanical behaviour of that kind of tissue. This fact can also explain the lower agreement between FE and DVC strain fields found for mixed metastatic vertebrae, reported in Fig. 7. Lastly, a limited number of vertebrae was analysed and should be increased in future validation studies. After the exclusion of the vertebrae not satisfying the requirements, only six metastatic vertebrae remained, three presenting a lytic condition and three presenting a mixed metastatic condition, respectively, in opposition to nine healthy vertebrae analysed.

In conclusion, the combination of the experimental DVC technique and the FE modelling technique presented here has enabled the development of a promising pipeline for the validation of in silico predictors of vertebral strength. It has also highlighted some criticalities which would need to be tackled in order to bring FE models to predict vertebral fracture. Two possible approaches could be followed to strengthen the validation outcome. On the one side, the use of higher resolution imaging techniques could provide a DVC accuracy sufficient to validate also the strain predictions of the FE models. However, imaging methods able to achieve this level of resolution cannot currently be applied to a whole vertebra but only to a portion of it, acquiring only some trabeculae at a time. On the other side, a more viable strategy could be an extensive tests campaign where the numerical failure load would be validated against experimental data, similarly to the work presented by Stadelmann et al. 2020, increasing the number of specimens to provide evidence of the accuracy of FE models on a different level. This would also allow to assess the influence on the failure load predictions of modelling choices such as the inclusion of plasticity or the constitutive models adopted for healthy and metastatic bone.

## Supplementary Information

Below is the link to the electronic supplementary material.

Supplementary file1 (PDF 4678 KB)

Supplementary file2 (DOCX 168 KB)

## Data Availability

Displacement and strain data (both numerical and experimental) are available at https://figshare.com/s/93bf4ab3ae3319325fdf.
