# Supplementary material for: Comparing the predictions of CT-based subject-specific finite element models of human metastatic vertebrae with digital volume correlation measurements
Source: Biomech Model Mechanobiol. 2025 Apr 19;24(3):1017–30. doi: 10.1007/s10237-025-01950-x (PMC12162702; doi:10.1007/s10237-025-01950-x)

Supplementary Materials 1

For each one of the analysed vertebrae the following information are reported:

- Scatter plots between DVC (horizontal axis) and FE (vertical axis) displacements
- Spatial distribution of the prediction error
- Distribution of the principal strains for both DVC and FE data

ID	Specimen	Vertebra	Type	Load step analysed	To be removed
1<br>2	770	L2	Lytic	--	X
		L3	Control	physio	
3<br>4	771	T11	Control	physio	
		T12	Mixed	--	X
5<br>6<br>7<br>8	772	T6	Lytic	failure	
		T10	Control	physio	
		T11	Mixed	physio	
		L3	Control	3x physio	
9<br>10	775	T10	Control	physio	
		L1	Control	3x physio	
11<br>12<br>13<br>14	780	T6	Mixed	3x physio	
		T7	Control	3x physio	
		T12	Mixed	3x physio	
		L1	Control	3x physio	
15	769	T11	Lytic	physio	
16	781	T11	Control	physio	
17	782	T5	Lytic	physio	
18	785	T7	Control	--	X (after comparison against the DVC uncertainty)

Table 1s. Specimen information. For each vertebra the identification number (ID), the specimen from which it has been exctracted, the vertebral level, the vertebral type and the load step at which it has been analysed have been reported. Additionally, vertebrae removed from the analysis have been highligthed in the last column

2 - Specimen 770 L3

- Fracture in the other vertebra at the failure step
- Control vertebra

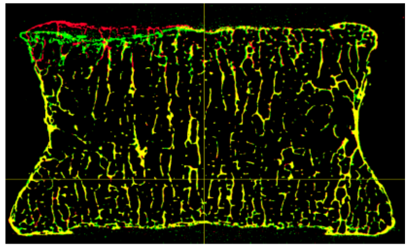

Displacements Correlations

ML

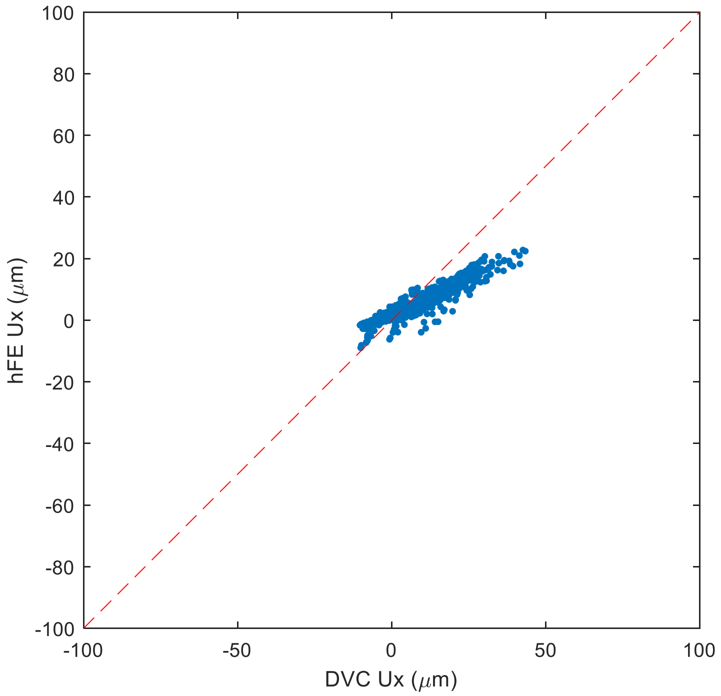

y = 0,46 x + 1,36
R^2 = 0,88
RMSE = 2 µm
RMSE% = 4 %

AP

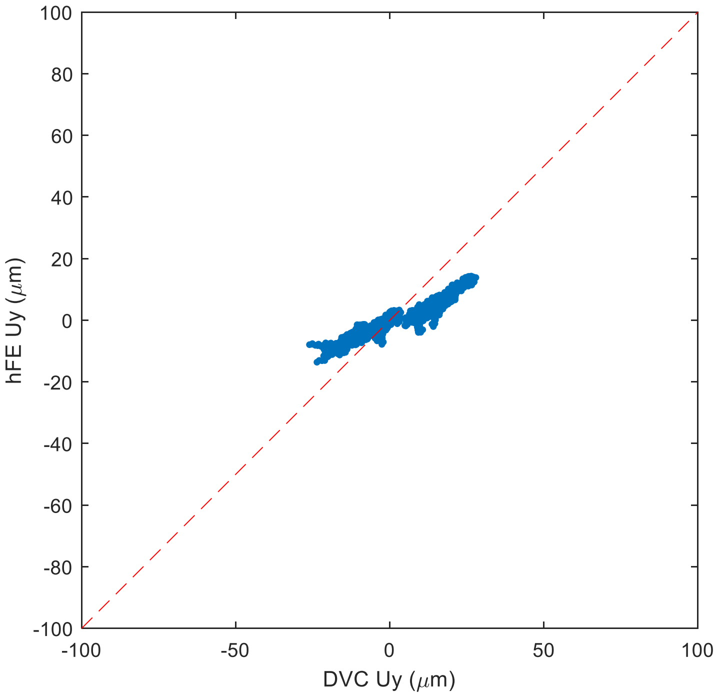

y = 0,43 x – 0,60
R^2 = 0,89
RMSE = 2 µm
RMSE% = 8 %

CC

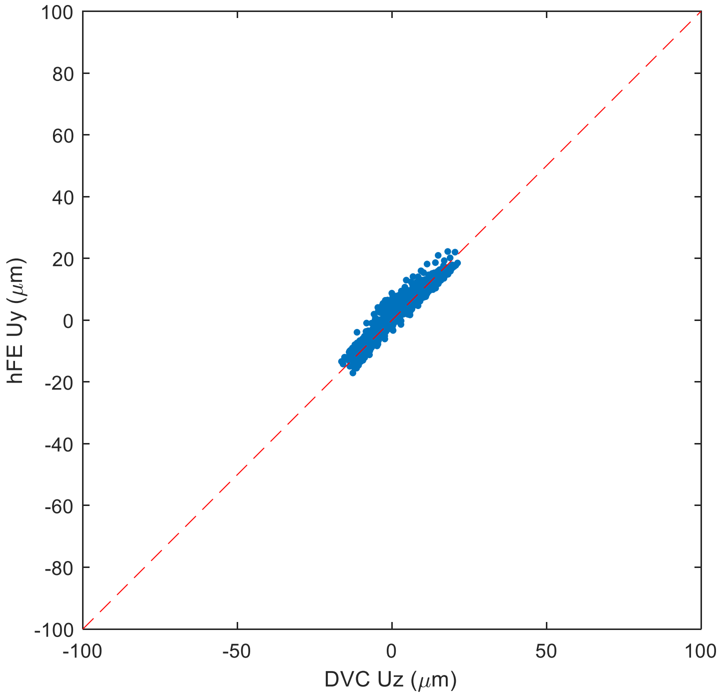

y = 0,90 x + 1,42
R^2 = 0,87
RMSE = 3 µm
RMSE% = 5 %

Spatial distribution of the errors

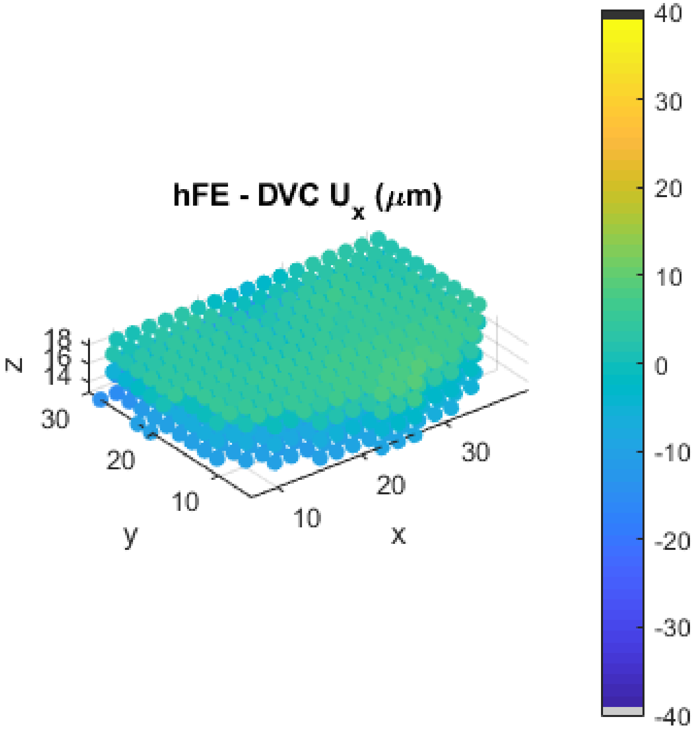

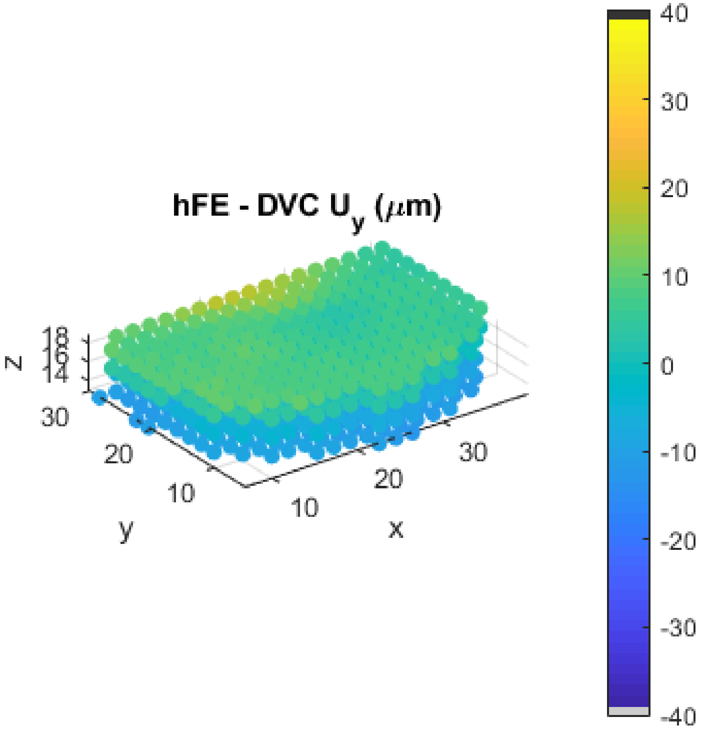

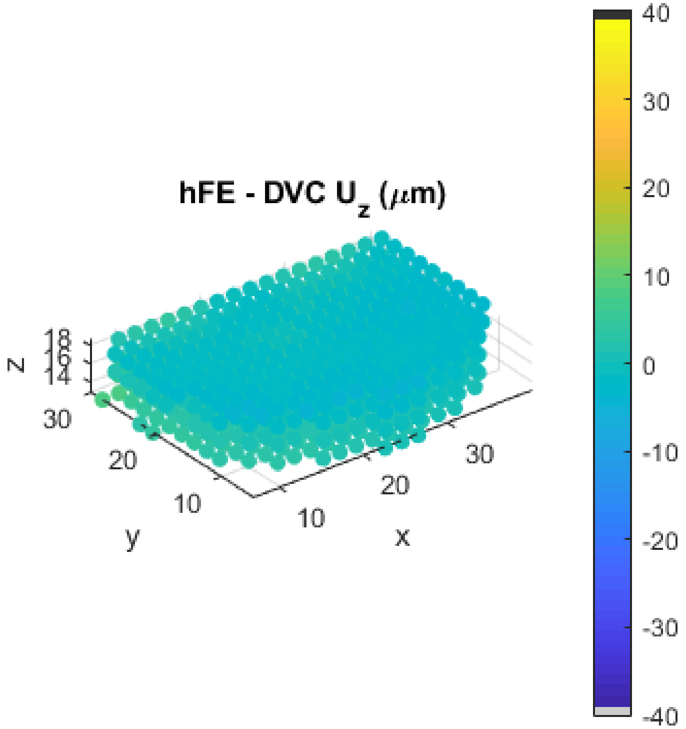

Qualitative comparison of the strains

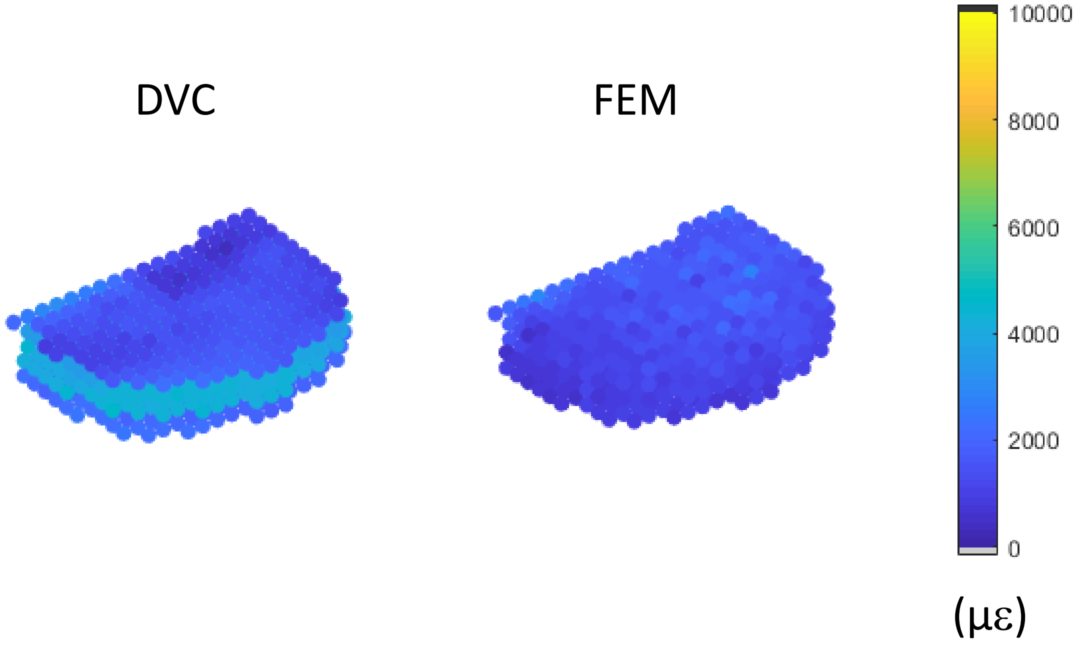

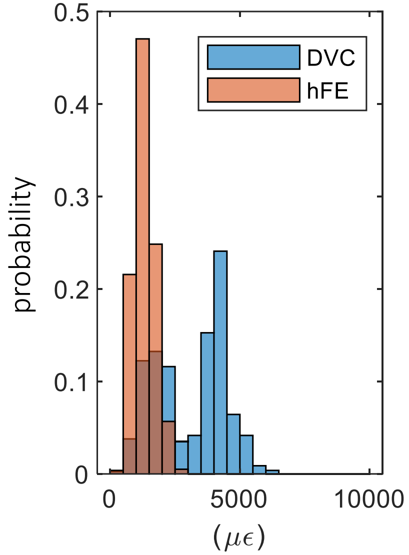

Maximum principal strains

Minimum principal strains

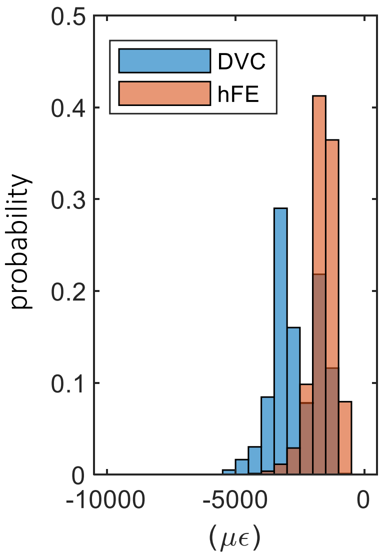

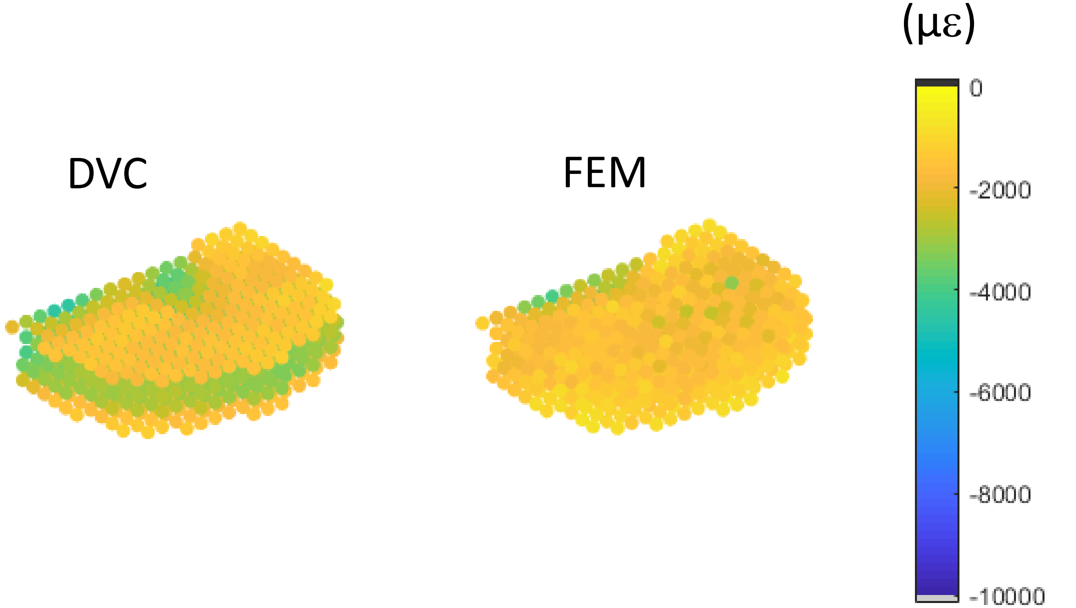

3 - Specimen 771 t11

- Fracture in the other vertebra at the failure step
- Control vertebra

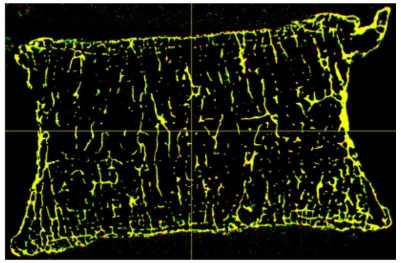

Displacements Correlations

ML

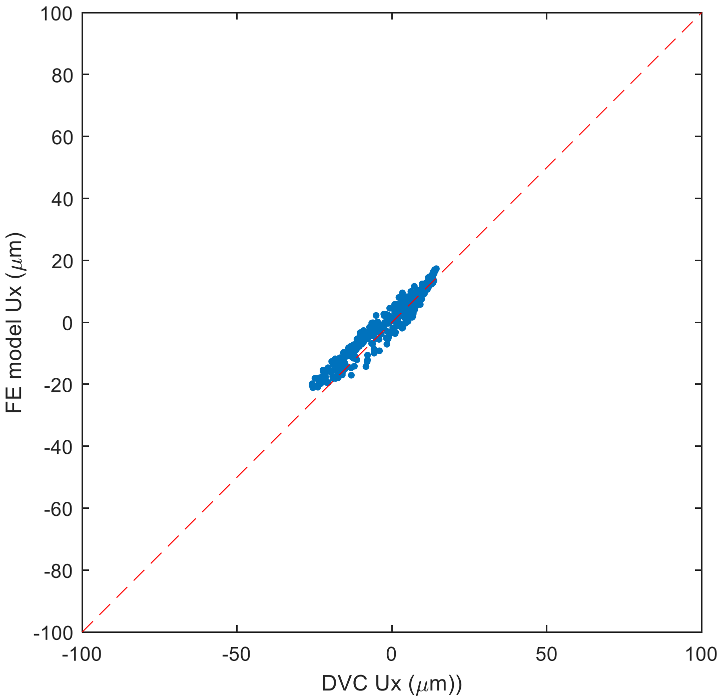

y = 0,89 x + 1,70
R^2 = 0,95
RMSE = 2 µm
RMSE% = 7 %

AP

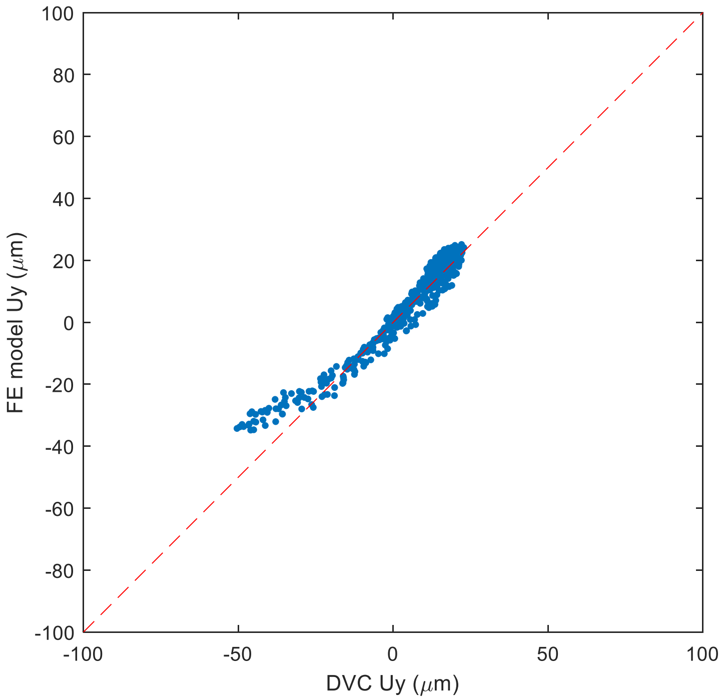

y = 0,90 x + 1,05
R^2 = 0,94
RMSE = 4 µm
RMSE% = 7 %

CC

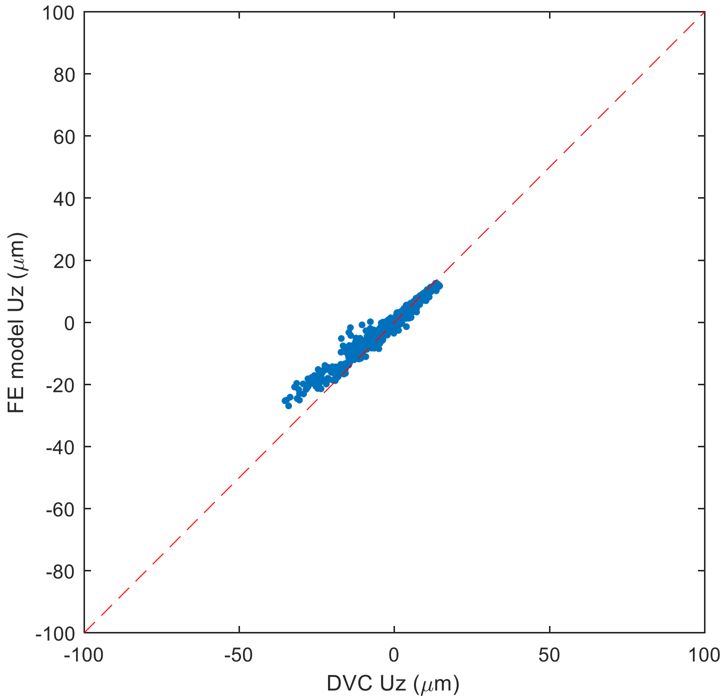

y = 0,84 x + 0,55
R^2 = 0,97
RMSE = 2 µm
RMSE% = 4 %

Spatial distribution of the errors

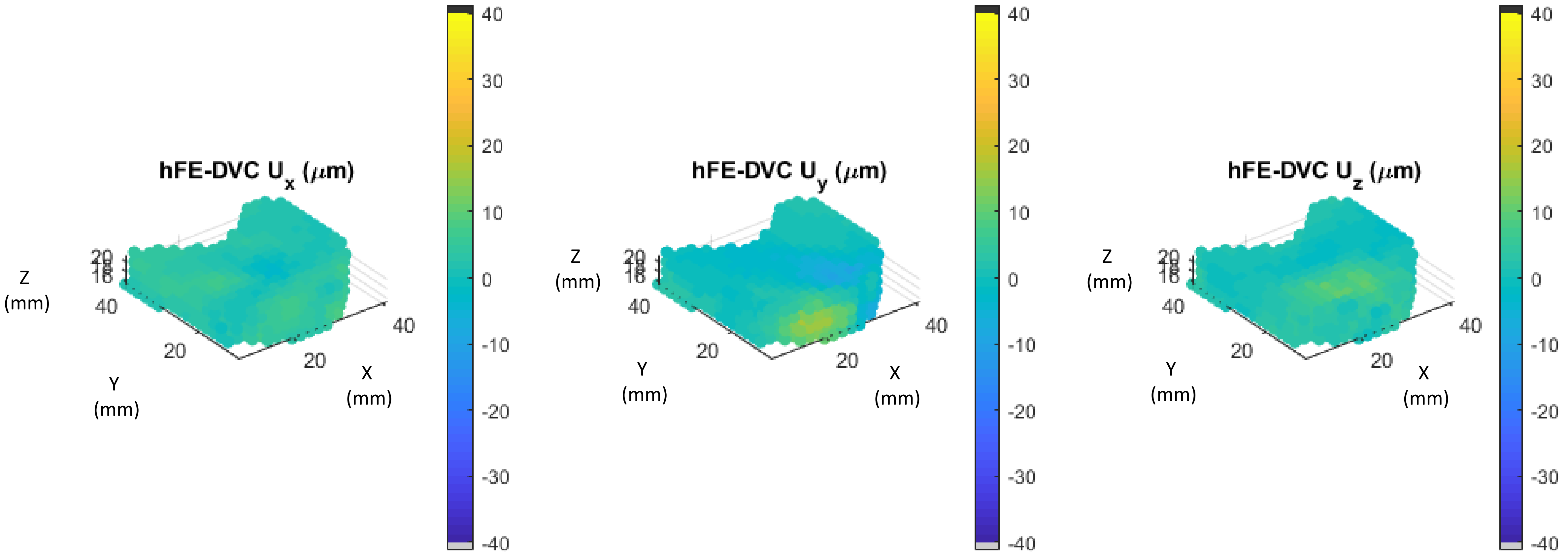

Qualitative comparison of the strains

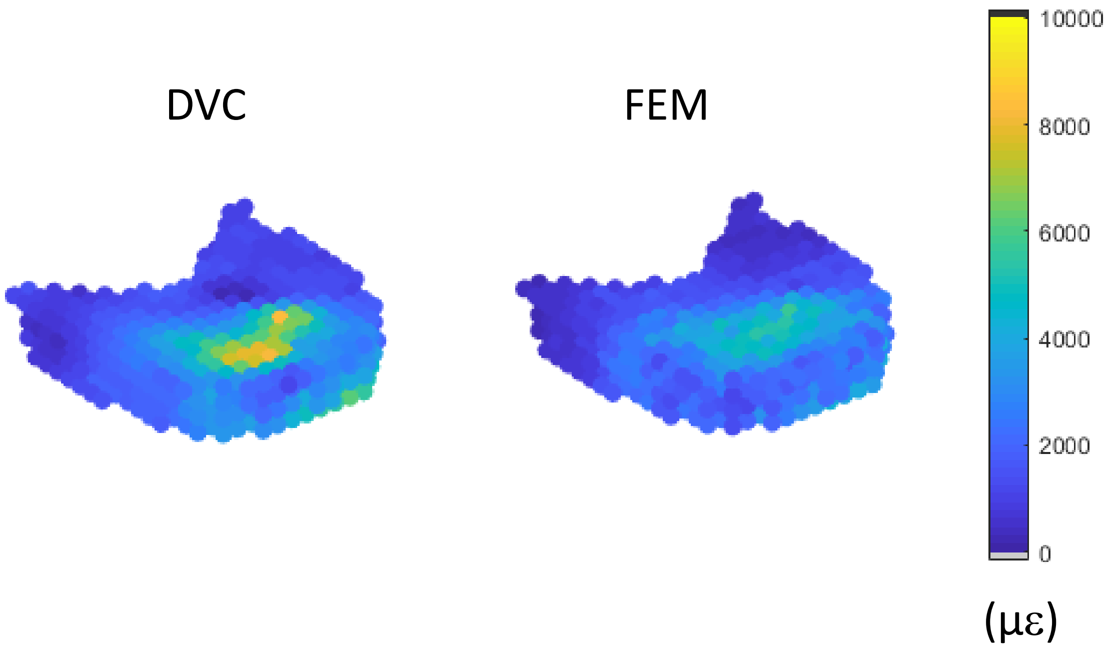

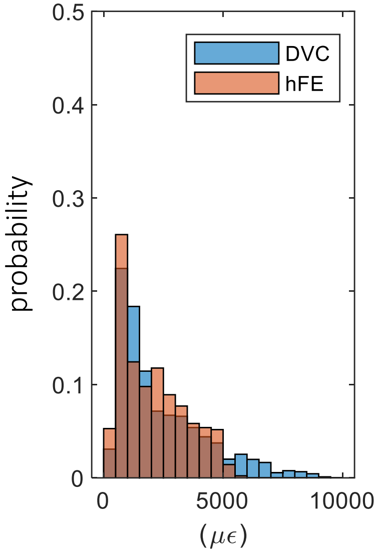

Maximum principal strains

Minimum principal strains

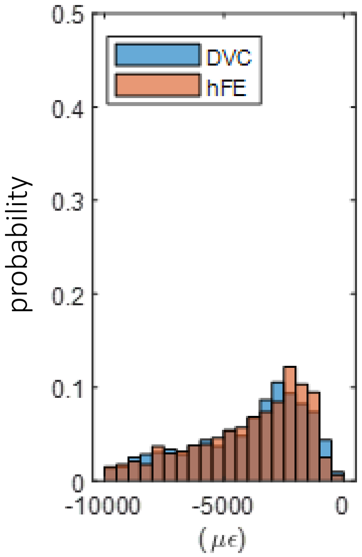

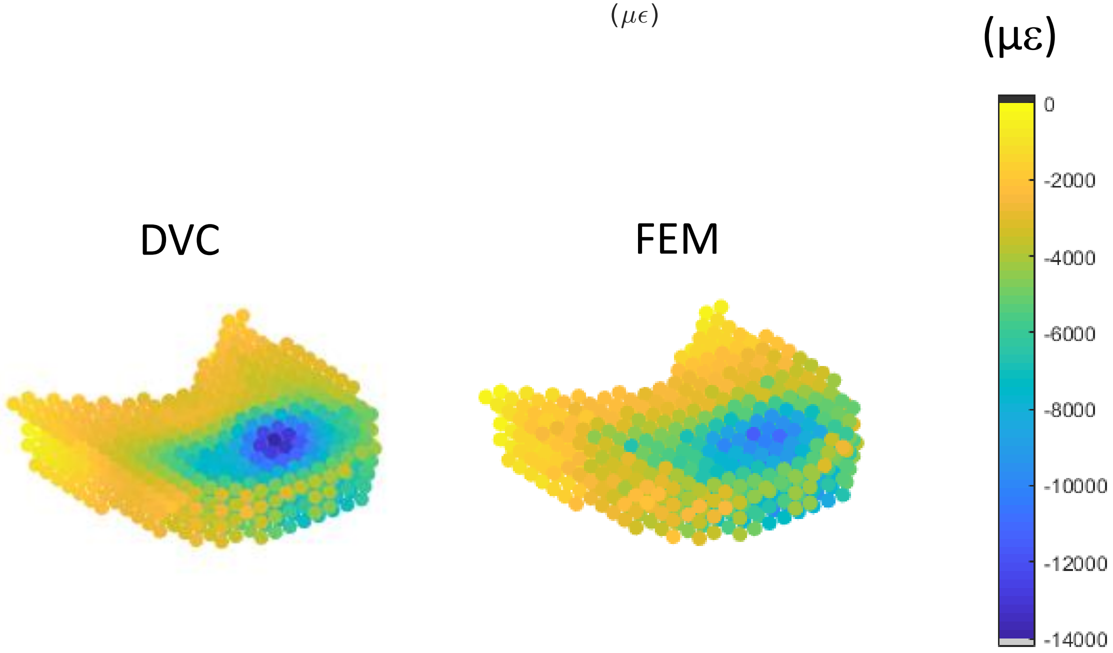

5 - Specimen 772 t6

- Fracture in this vertebra at the failure step
- Metastatic vertebra (lytic)

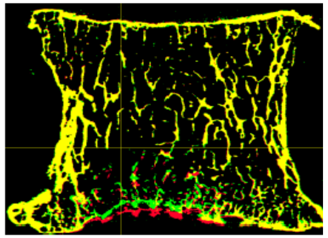

Displacements Correlations

ML

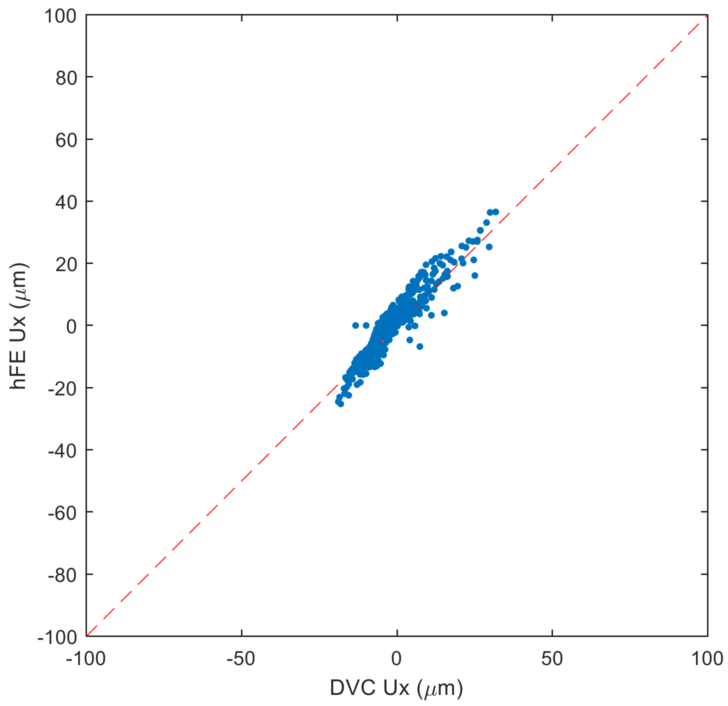

y = 1,06 x + 1,66
R^2 = 0,89
RMSE = 3 μm
RMSE% = 11 %

AP

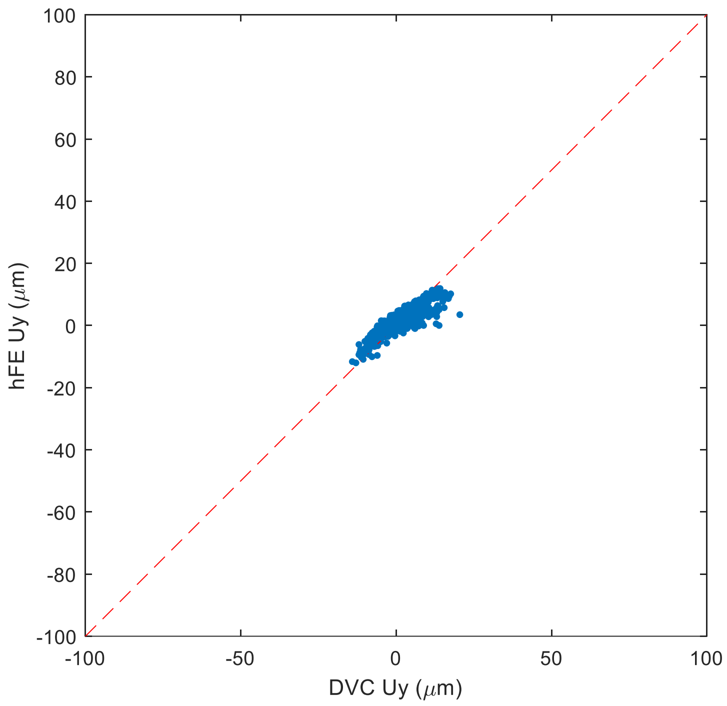

y = 0,64 x + 0,72
R^2 = 0,79
RMSE = 2 μm
RMSE% = 12 %

CC

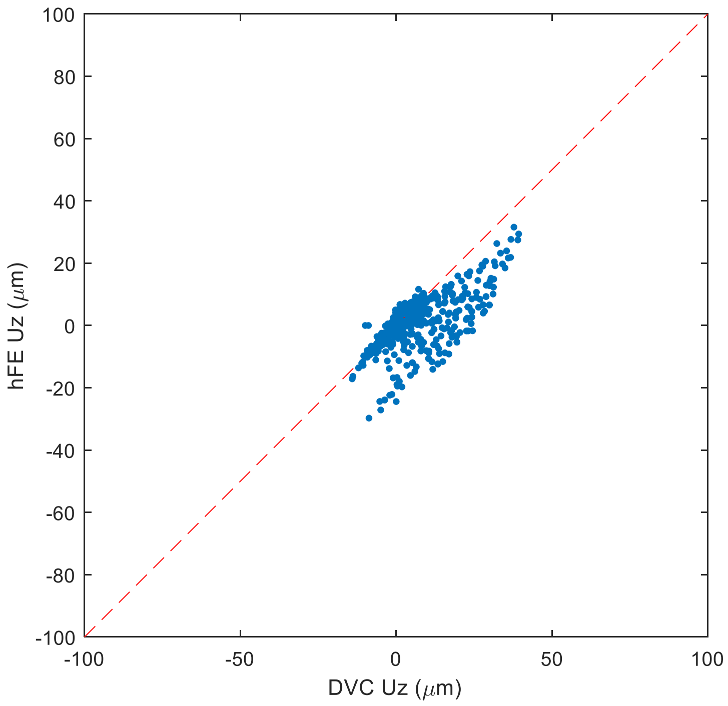

y = 0,53 x – 2,66
R^2 = 0,40
RMSE = 7 μm
RMSE% = 17 %

Spatial distribution of the errors

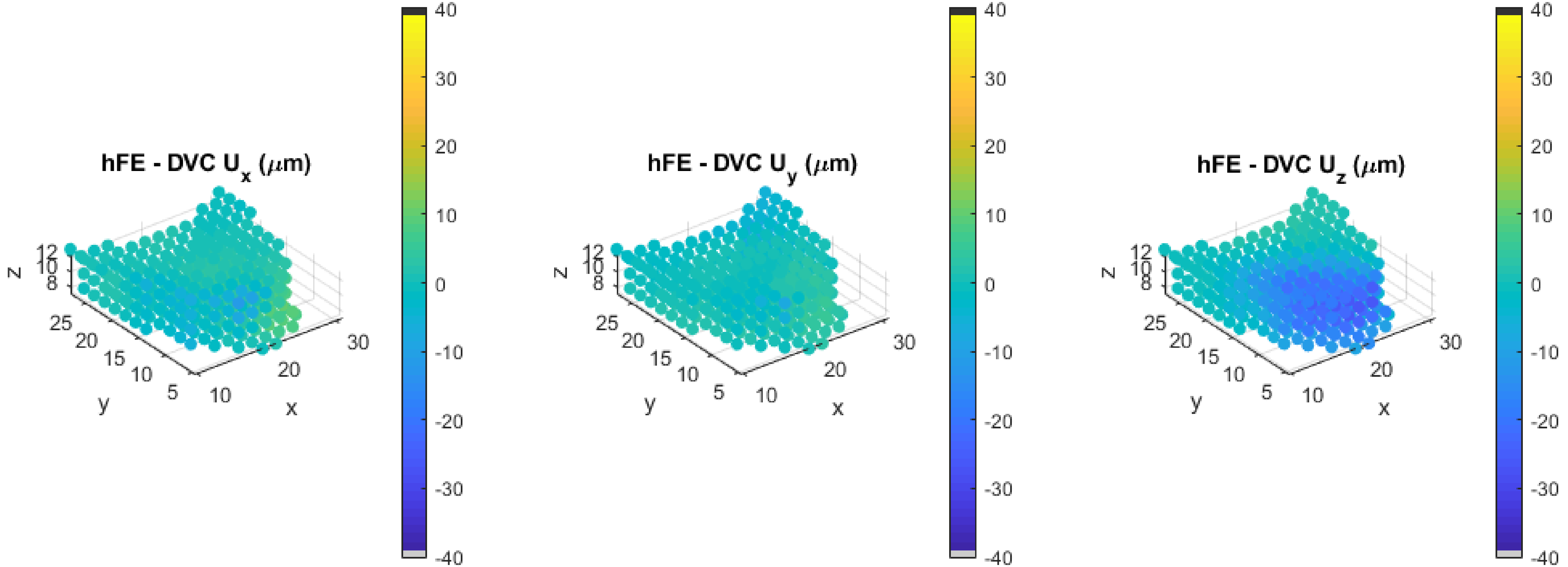

Qualitative comparison of the strains

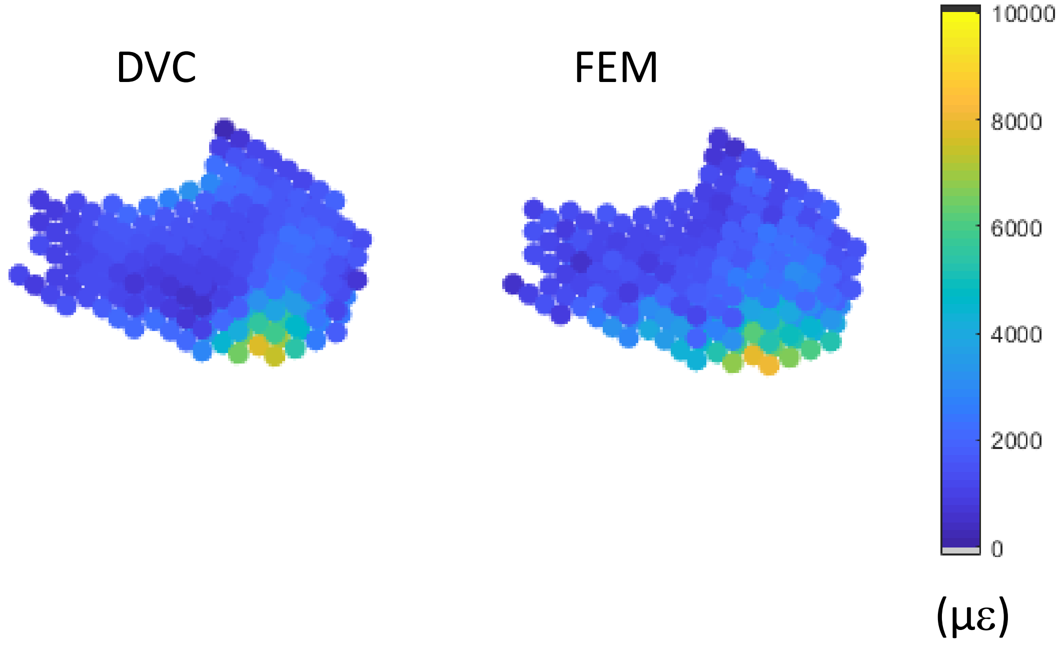

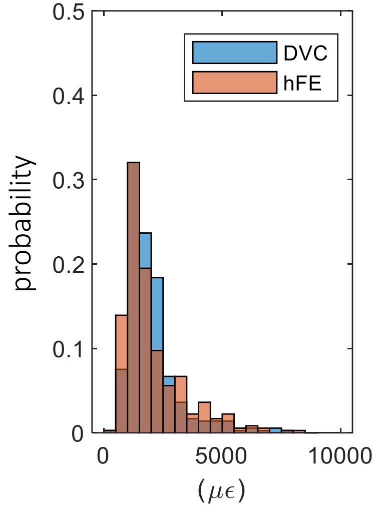

Maximum principal strains

Minimum principal strains

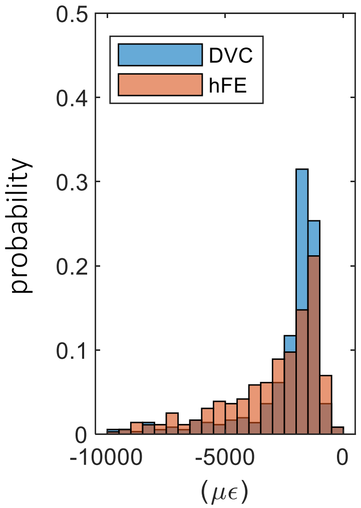

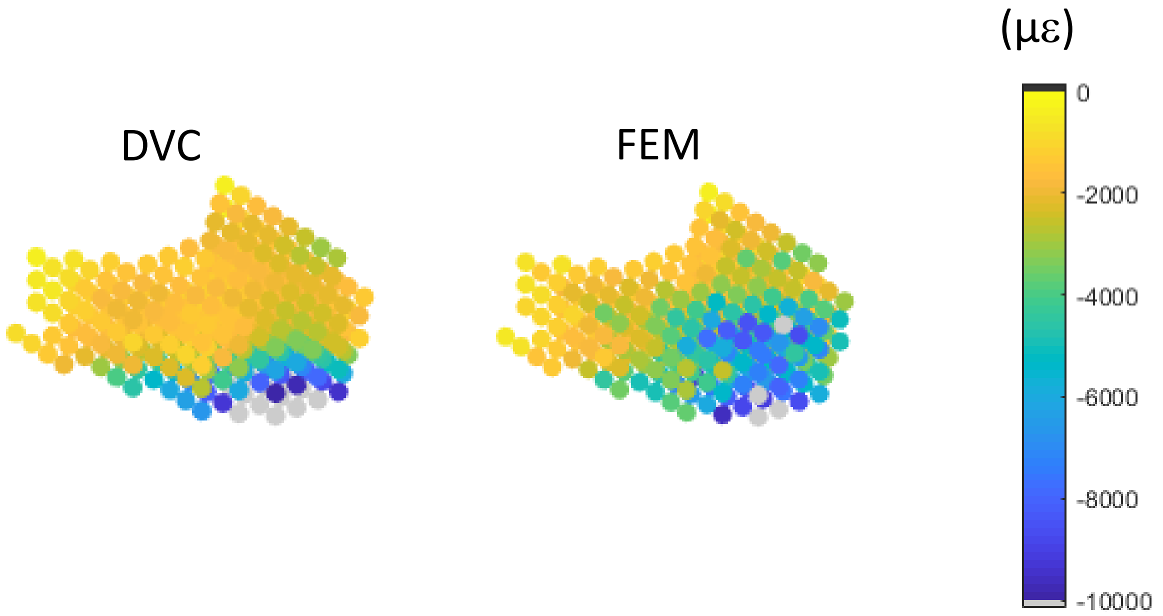

6 - Specimen 772 t10

- Fracture in the other vertebra at the failure step
- Control vertebra

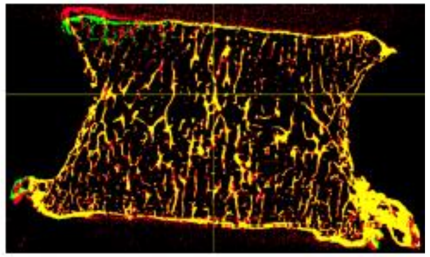

Displacements Correlations

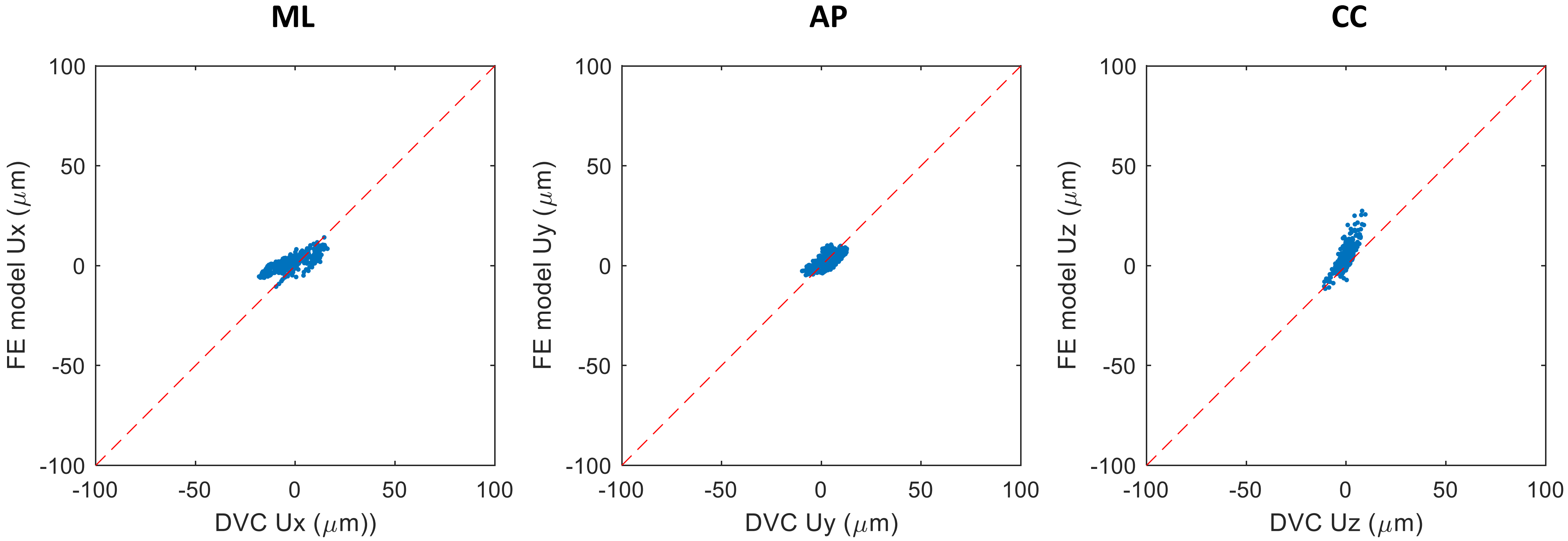

y = 0,55 x + 2,95
R^2 = 0,74
RMSE = 3,08 µm
RMSE% = 14 %

y = 0,56 x + 0,52
R^2 = 0,58
RMSE = 2,64 µm
RMSE% = 16 %

y = 1,32 x + 3,84
R^2 = 0,75
RMSE = 3,76 µm
RMSE% = 16 %

Spatial distribution of the errors

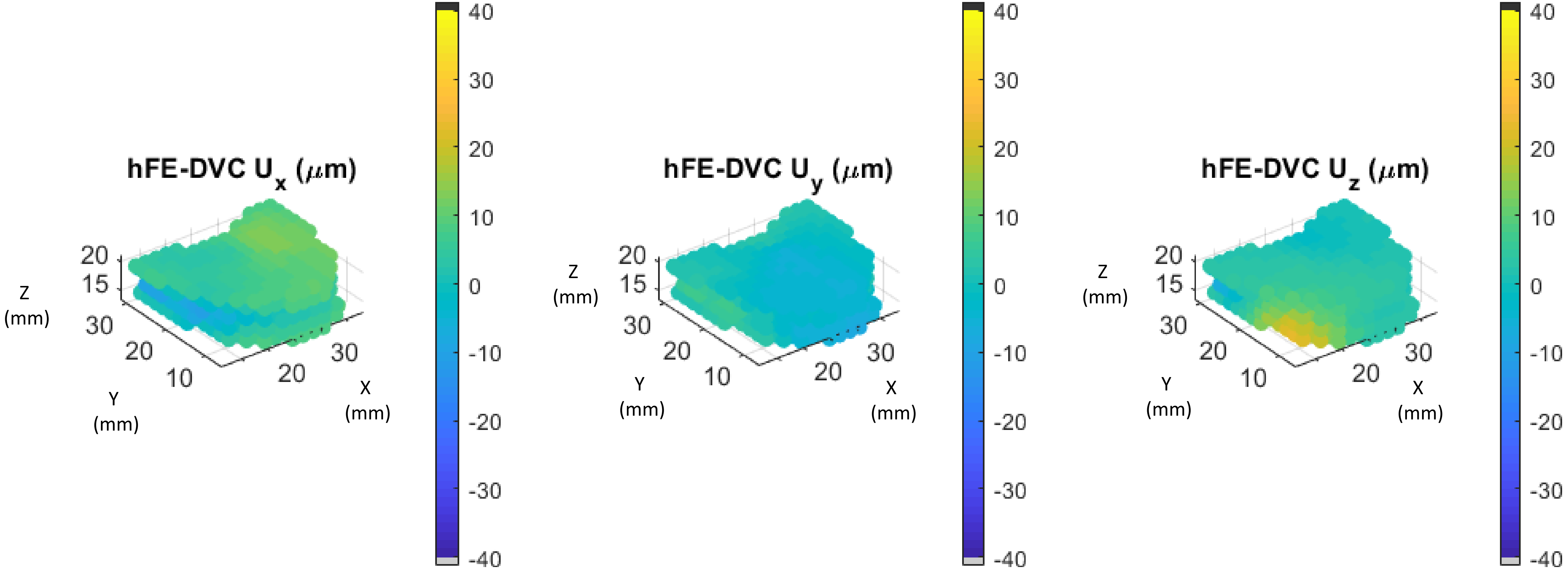

Qualitative comparison of the strains

DVC FEM

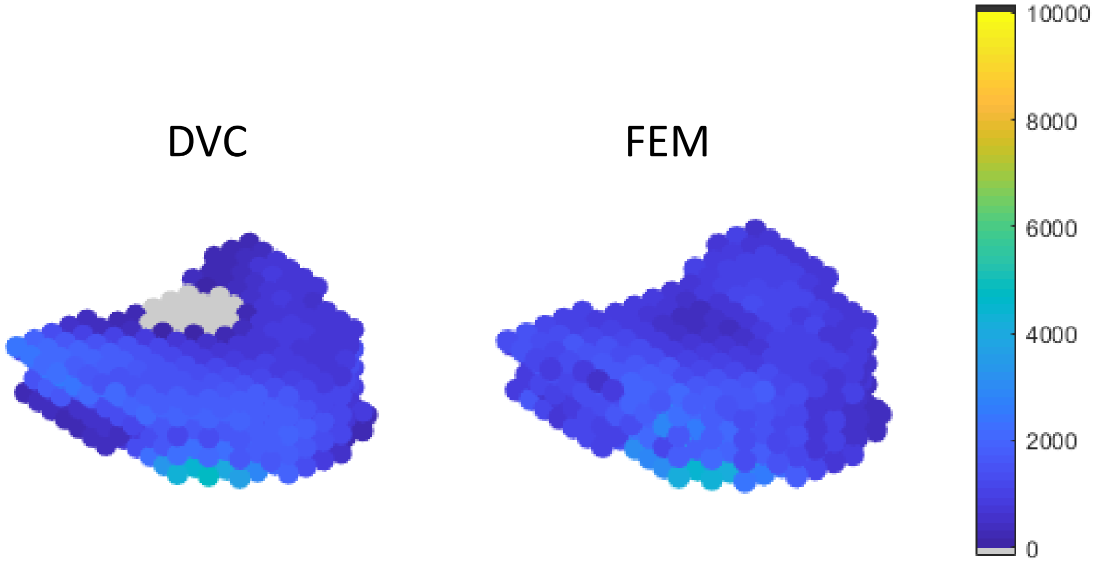

(με)

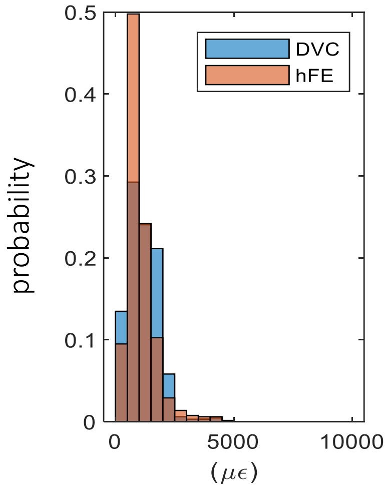

Maximum principal strains

Minimum principal strains

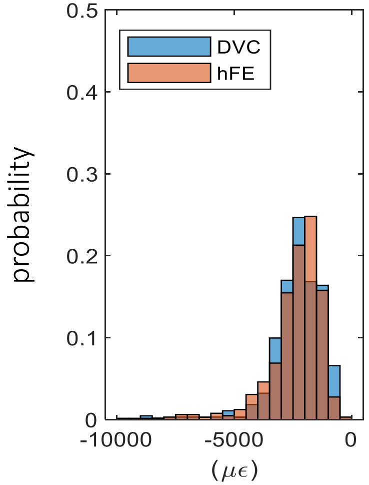

DVC FEM

(με)

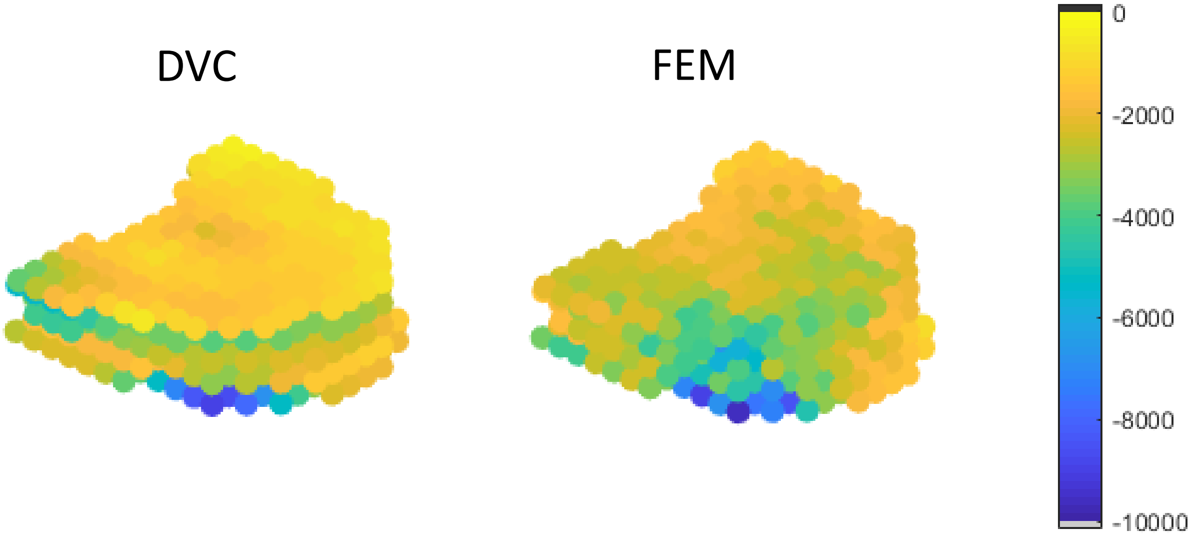

7 - Specimen 772 t11

- Fracture in this vertebra at the failure step
- Metastatic vertebra (mixed)

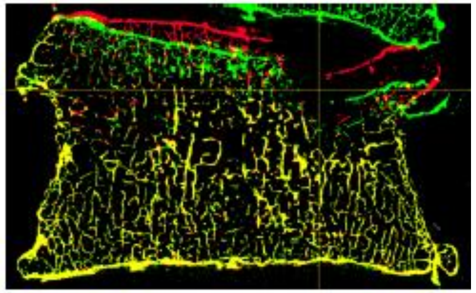

Displacements Correlations

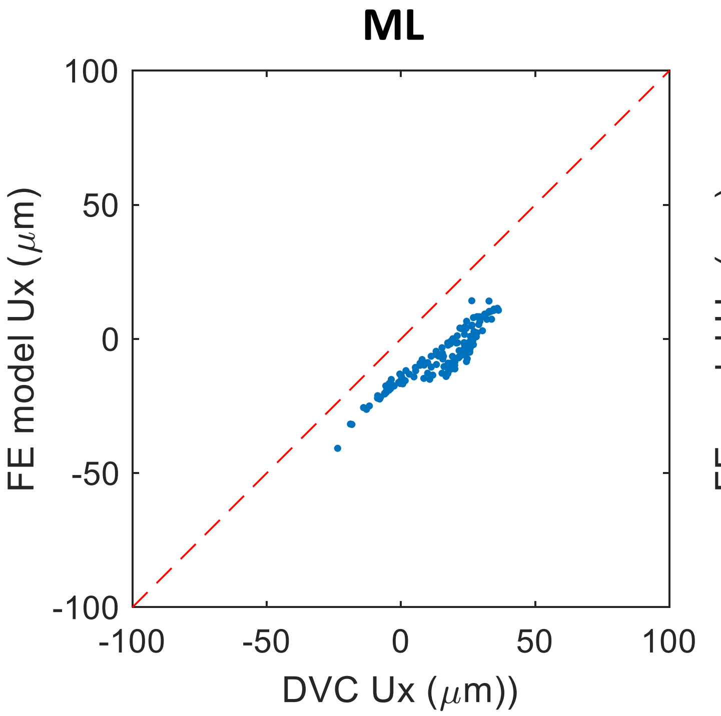

y = 0,63 x – 12,40
R^2 = 0,77
RMSE = 5,78 μm
RMSE% = 11 %

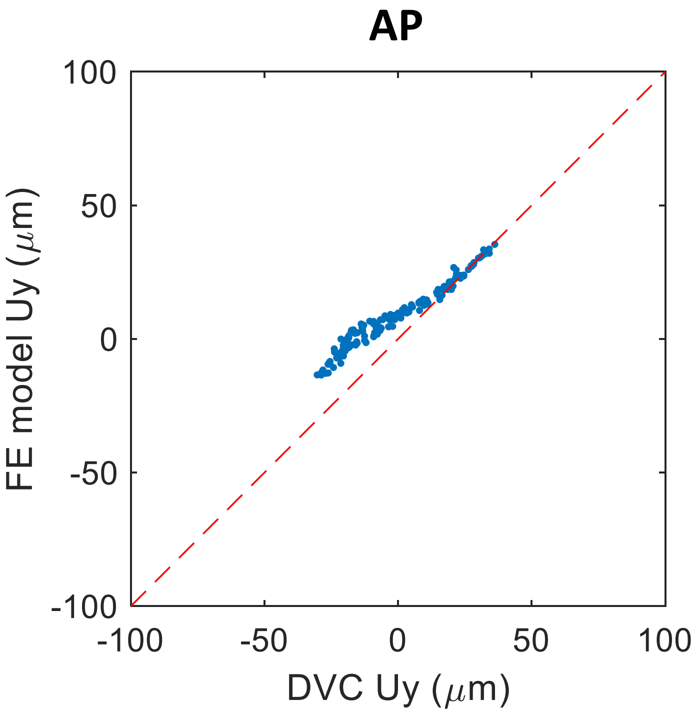

y = 0,69 x + 7,50
R^2 = 0,95
RMSE = 3,11 μm
RMSE% = 5 %

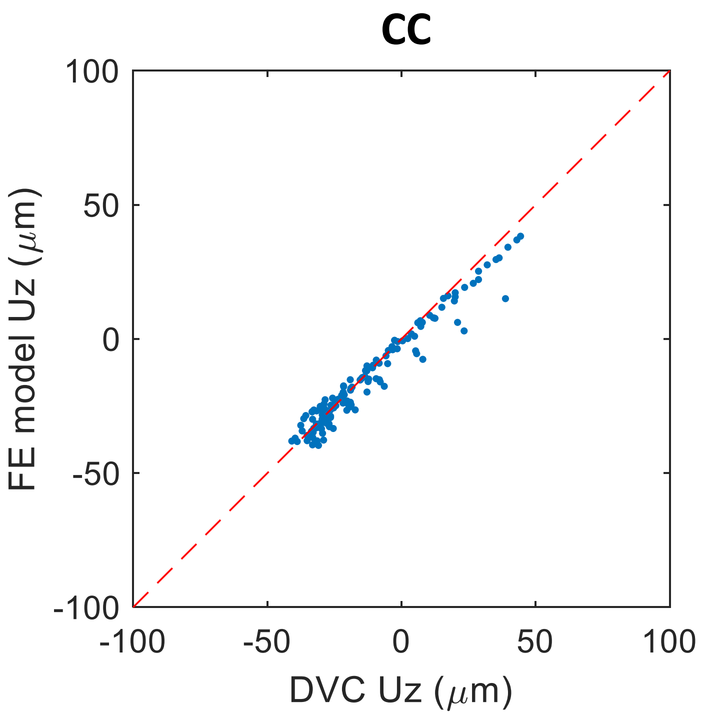

y = 0,91 x – 1,87
R^2 = 0,95
RMSE = 4,15 μm
RMSE% = 9 %

Spatial distribution of the errors

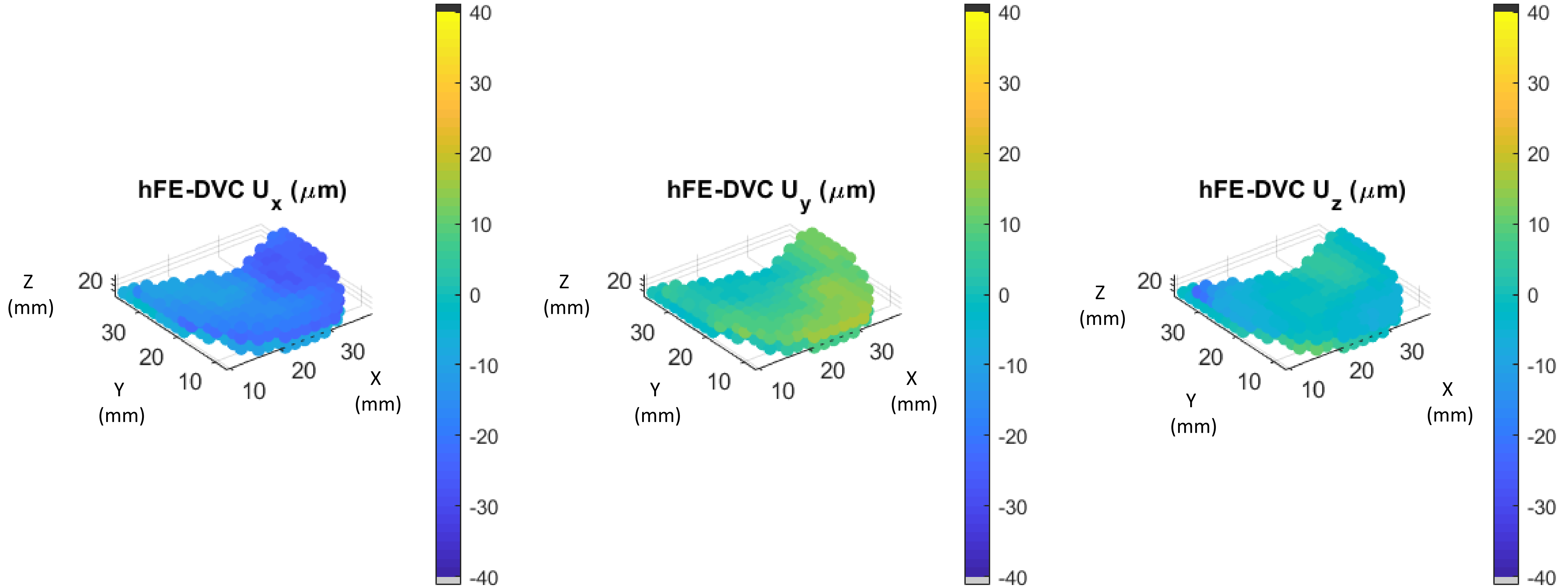

Qualitative comparison of the strains

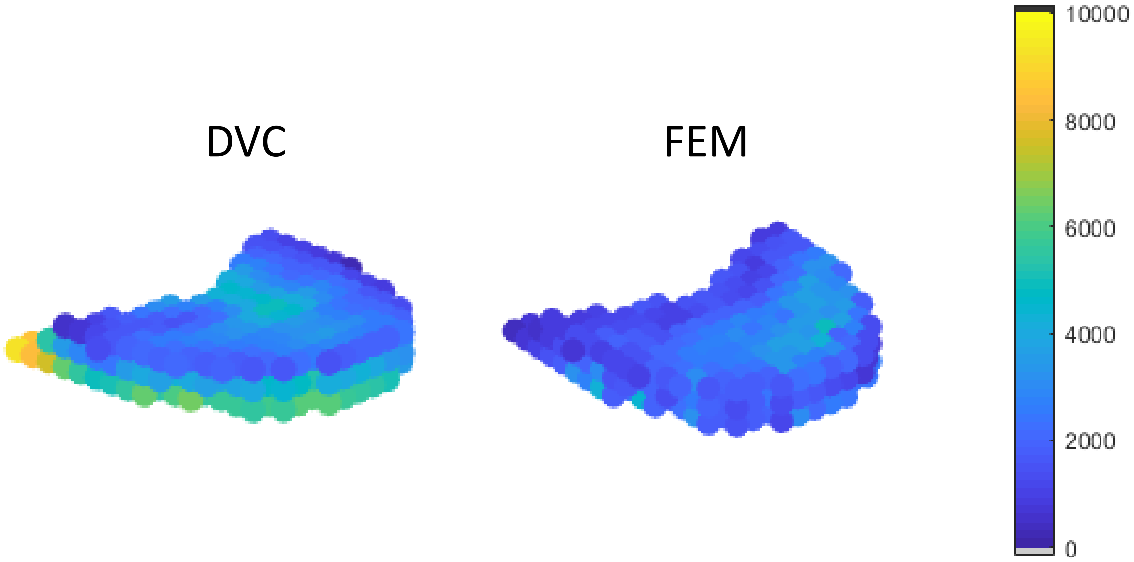

(με)

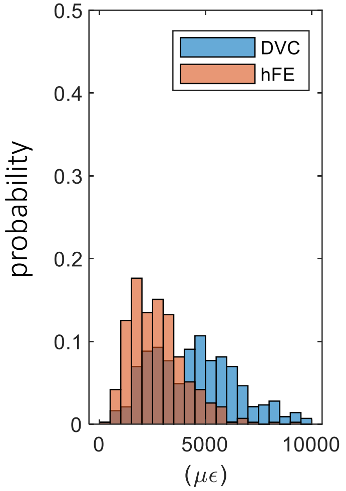

Maximum principal strains

(με)

Minimum principal strains

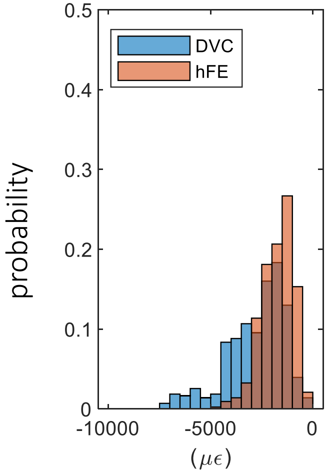

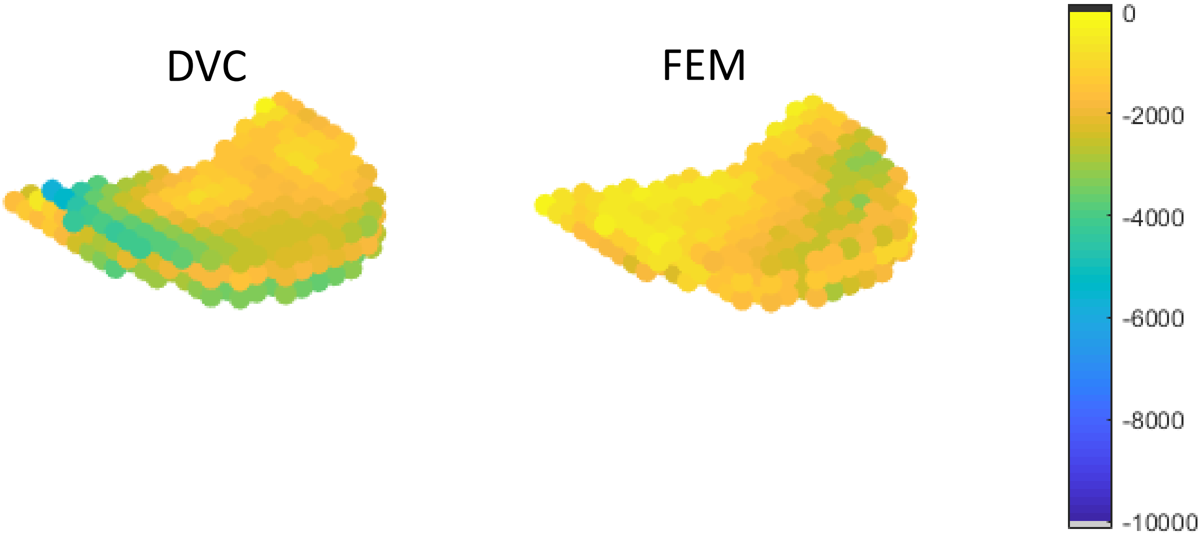

8 - Specimen 772 L3

- Fracture in the other vertebra at the failure step
- Control vertebra

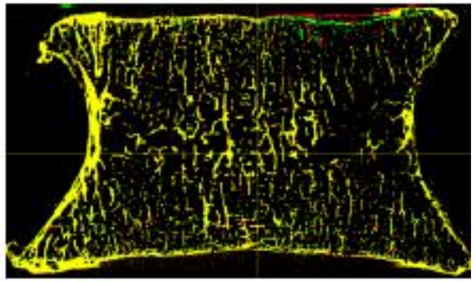

Displacements Correlations

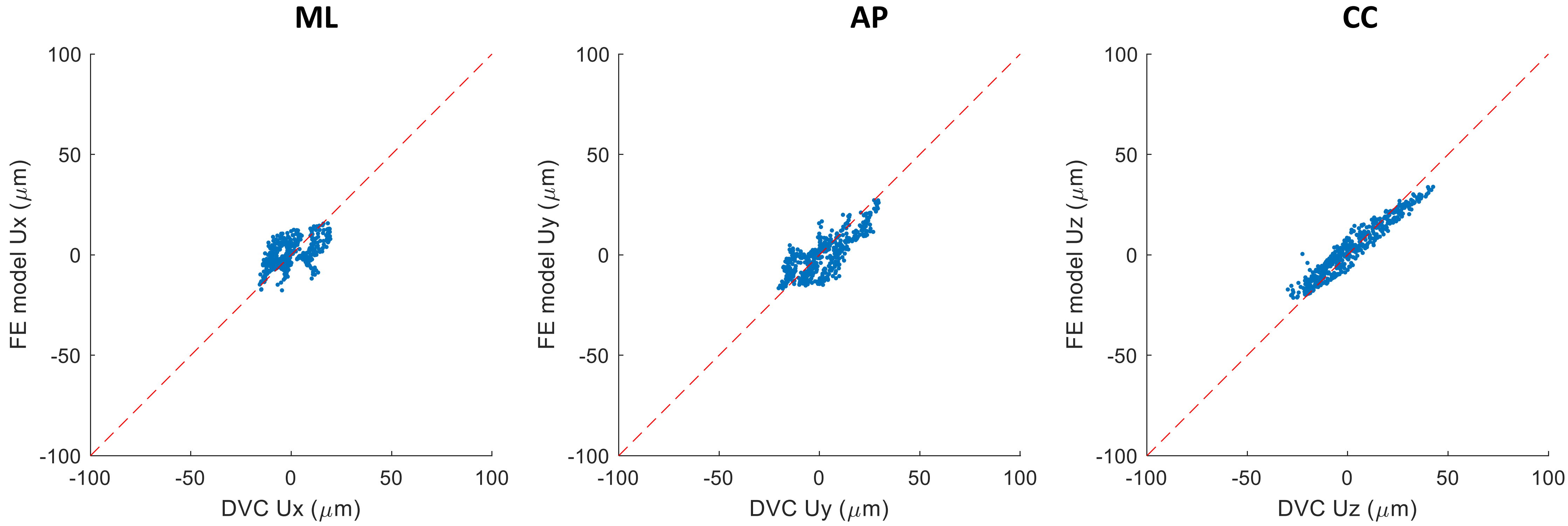

y = 0,42 x – 0,21
R^2 = 0,42
RMSE = 5,4 µm
RMSE% = 22 %

y = 0,66 x – 1,35
R^2 = 0,76
RMSE = 5,5 µm
RMSE% = 13 %

y = 0,85 x + 1,13
R^2 = 0,93
RMSE = 3,9 µm
RMSE% = 8 %

Spatial distribution of the errors

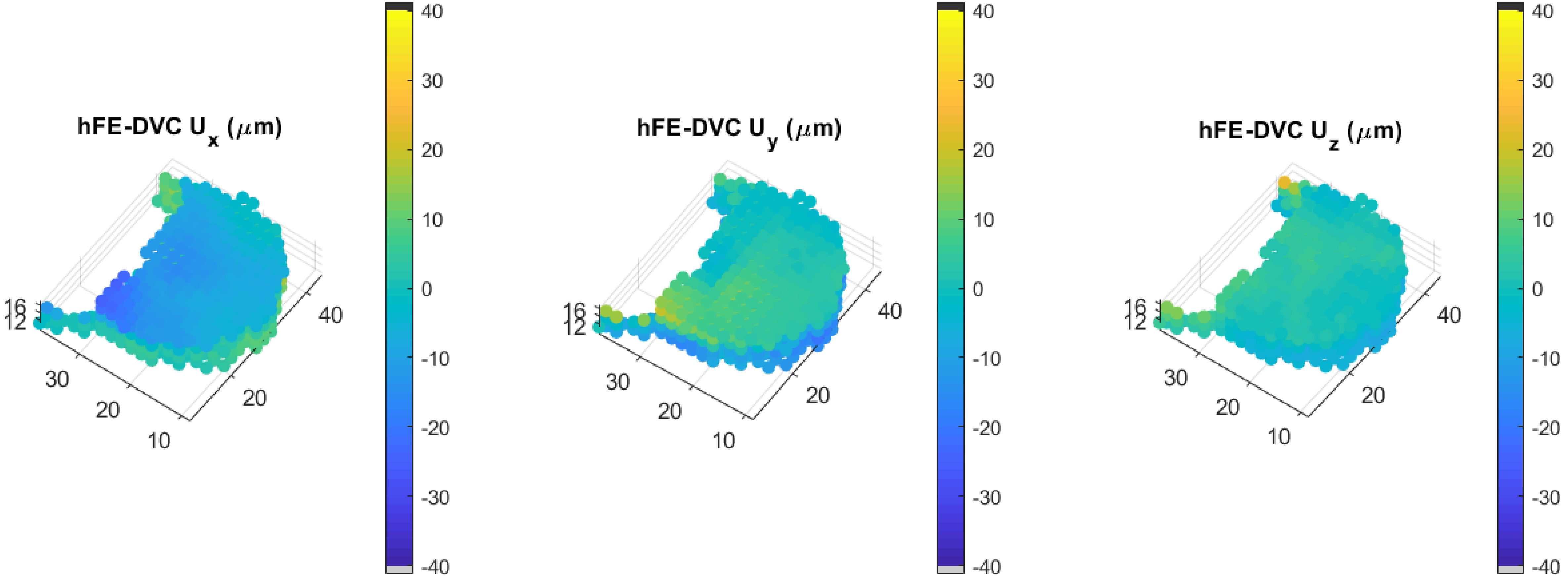

Qualitative comparison of the strains

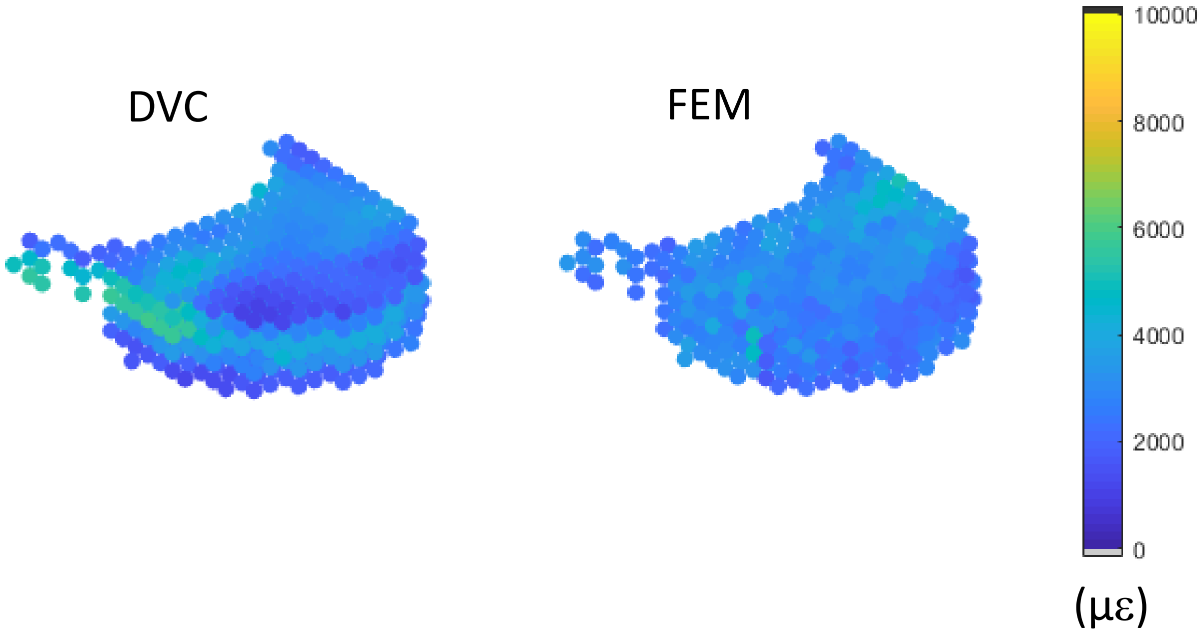

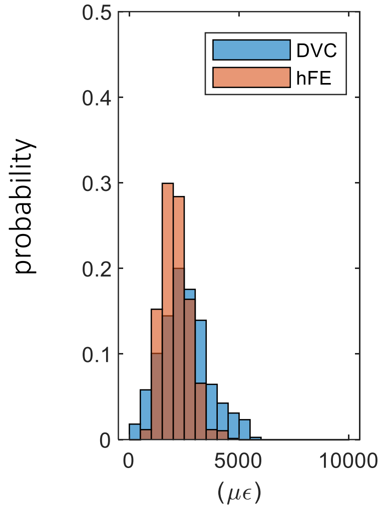

Maximum principal strains

Minimum principal strains

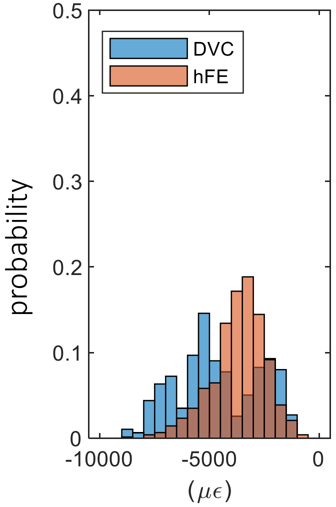

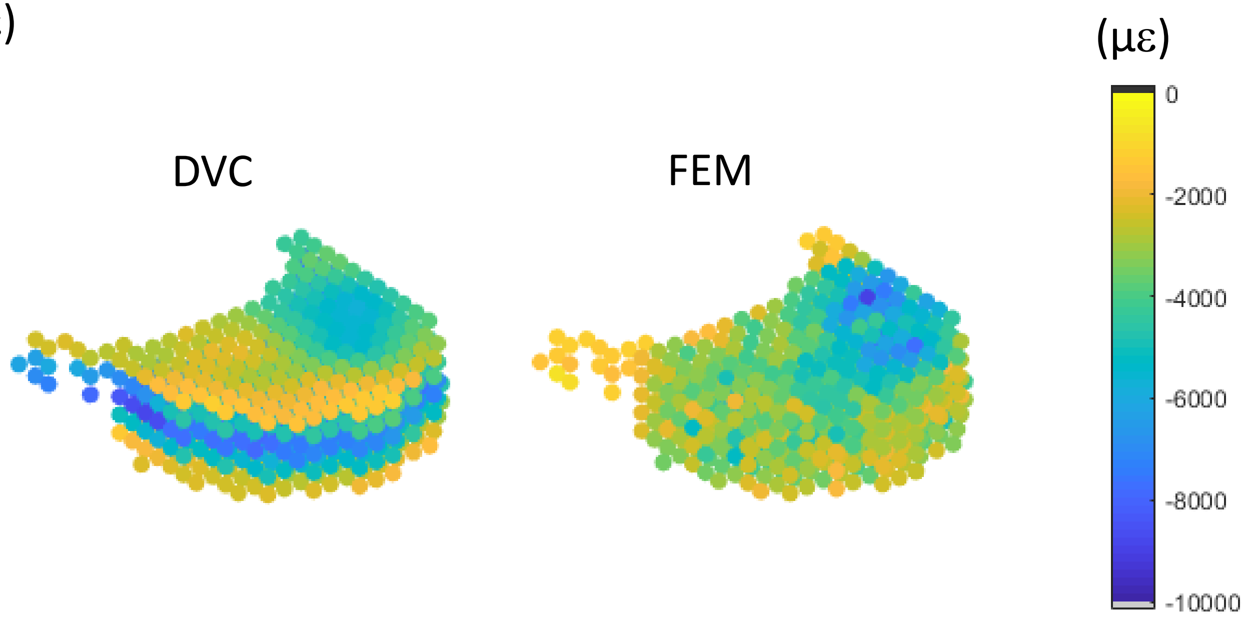

9 - Specimen 775 t10

- Fracture in the other vertebra at the failure step
- Control vertebra

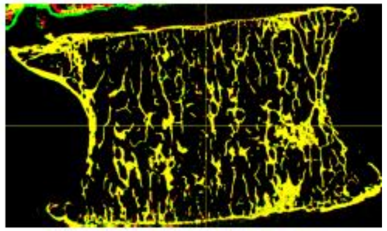

Displacements Correlations

ML

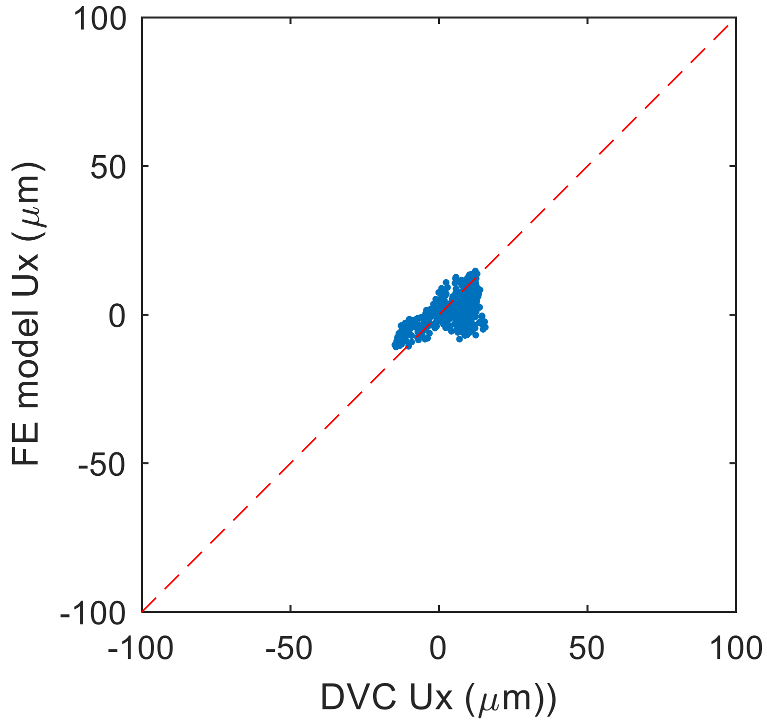

y = 0,44 x – 1,00
R^2 = 0,41
RMSE = 4,3 μm
RMSE% = 20 %

AP

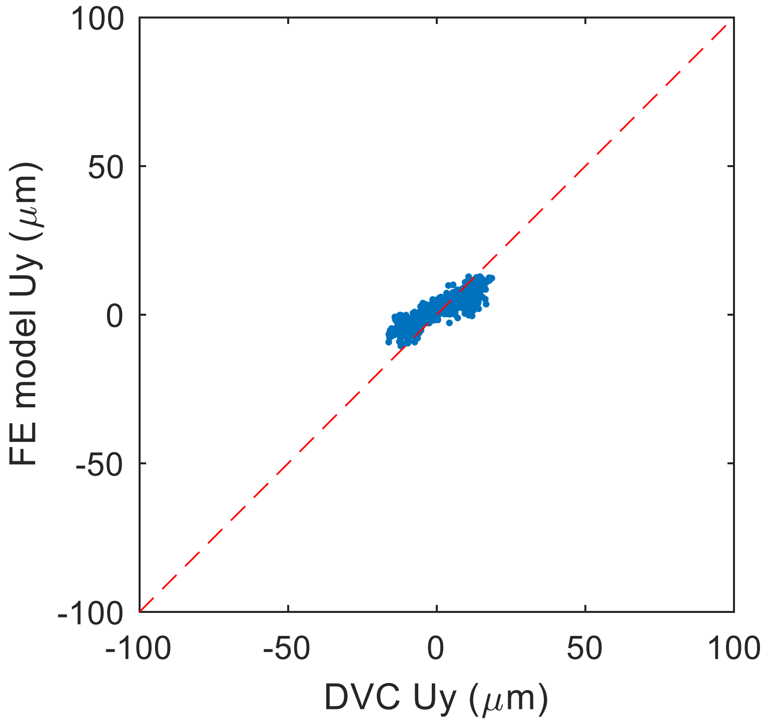

y = 0,49 x + 1,31
R^2 = 0,75
RMSE = 2,4 μm
RMSE% = 12 %

CC

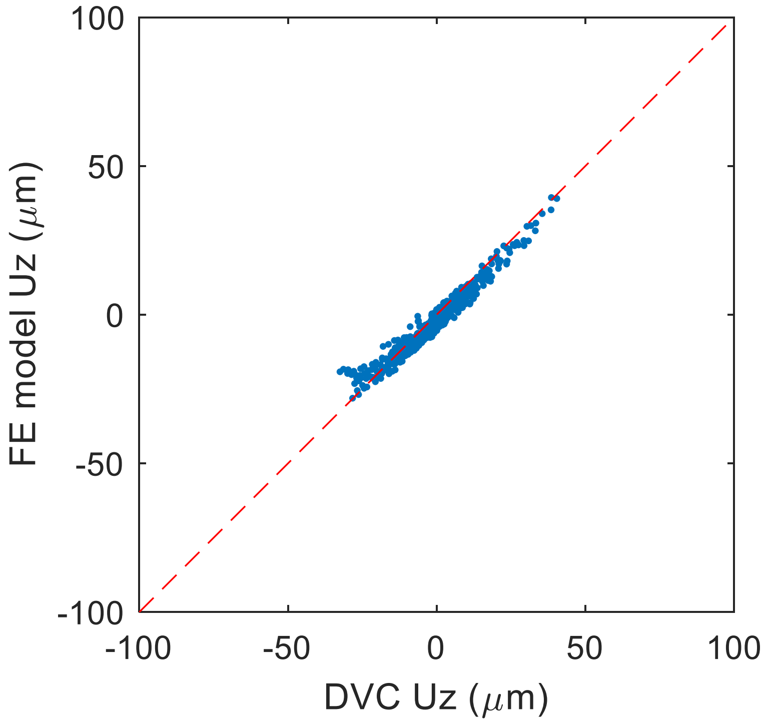

y = 0,85 x – 1,60
R^2 = 0,95
RMSE = 2,4 μm
RMSE% = 6 %

Spatial distribution of the errors

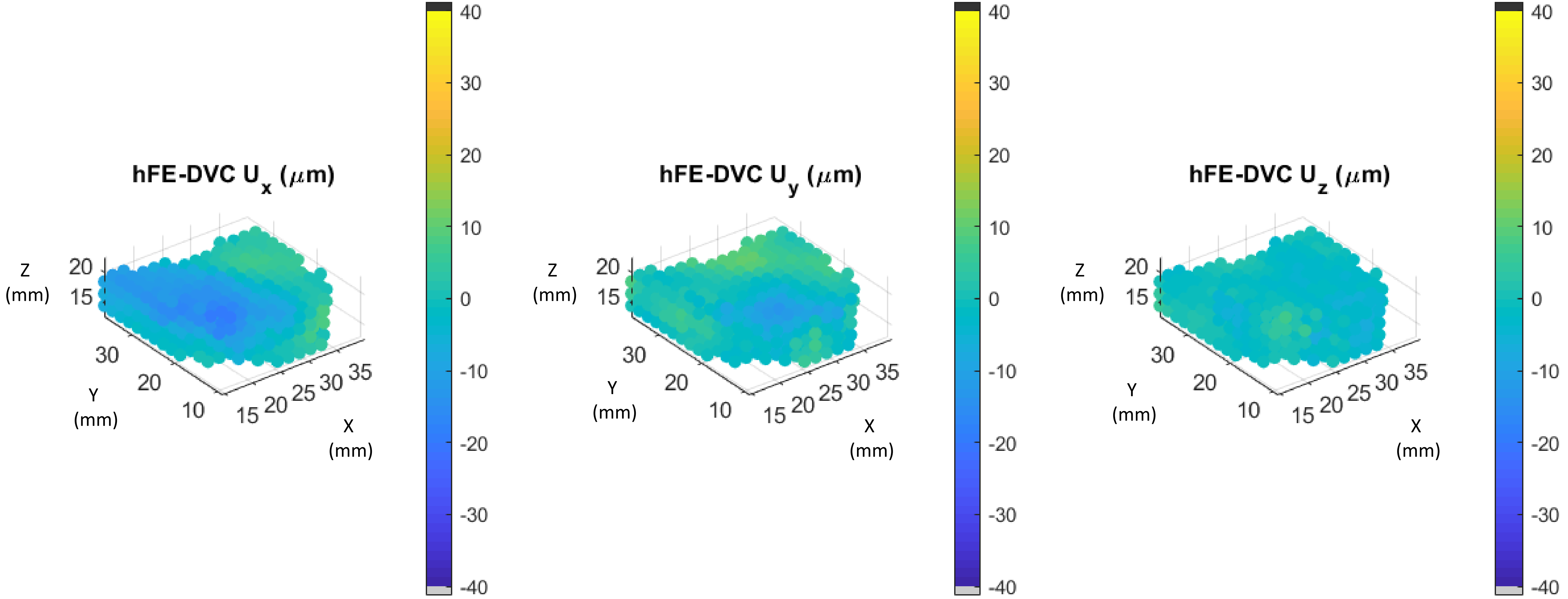

Qualitative comparison of the strains

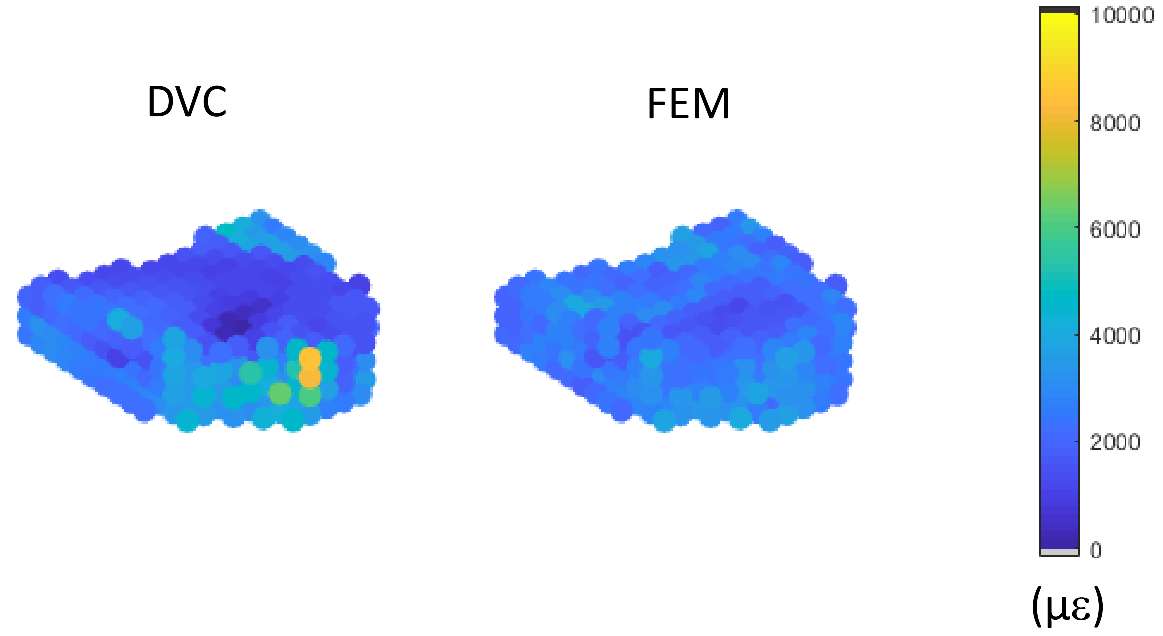

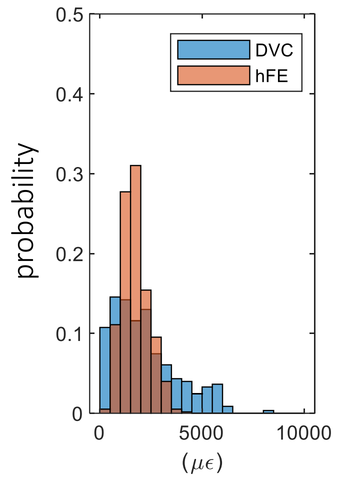

Maximum principal strains

Minimum principal strains

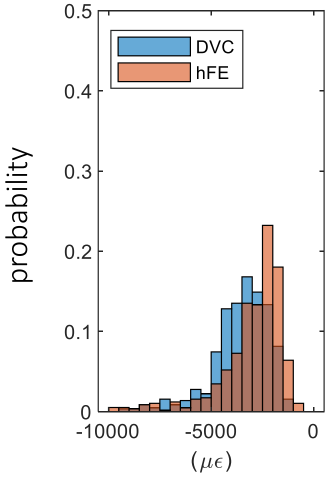

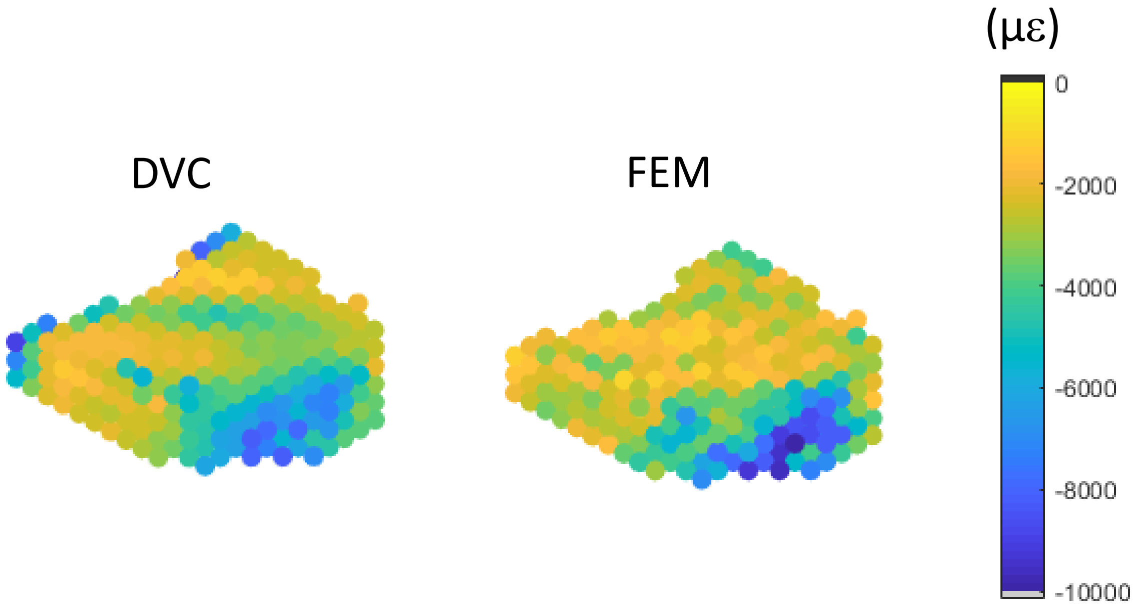

10 - Specimen 775 L1

- Fracture in this vertebra at the failure step
- Control vertebra

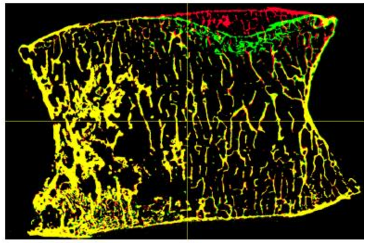

Displacements Correlations

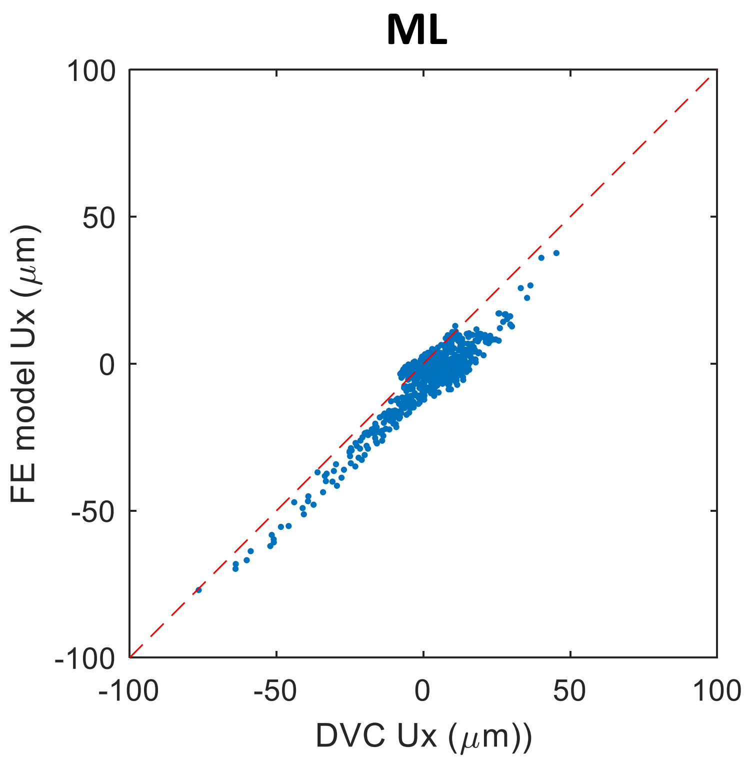

y = 0,91 x – 6,25
R^2 = 0,87
RMSE = 5,1 µm
RMSE% = 6 %

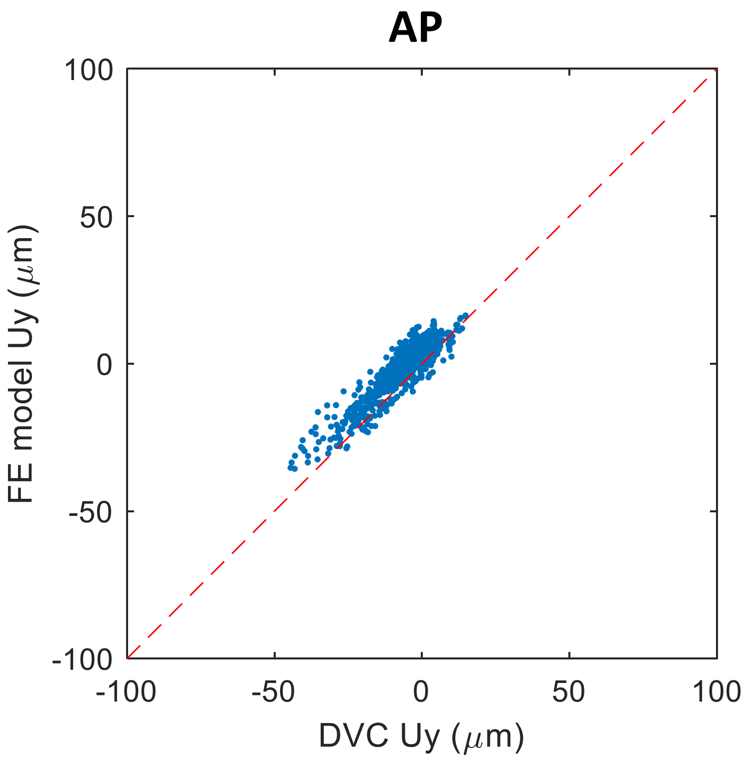

y = 0,94 x + 5,26
R^2 = 0,86
RMSE = 4,4 µm
RMSE% = 8 %

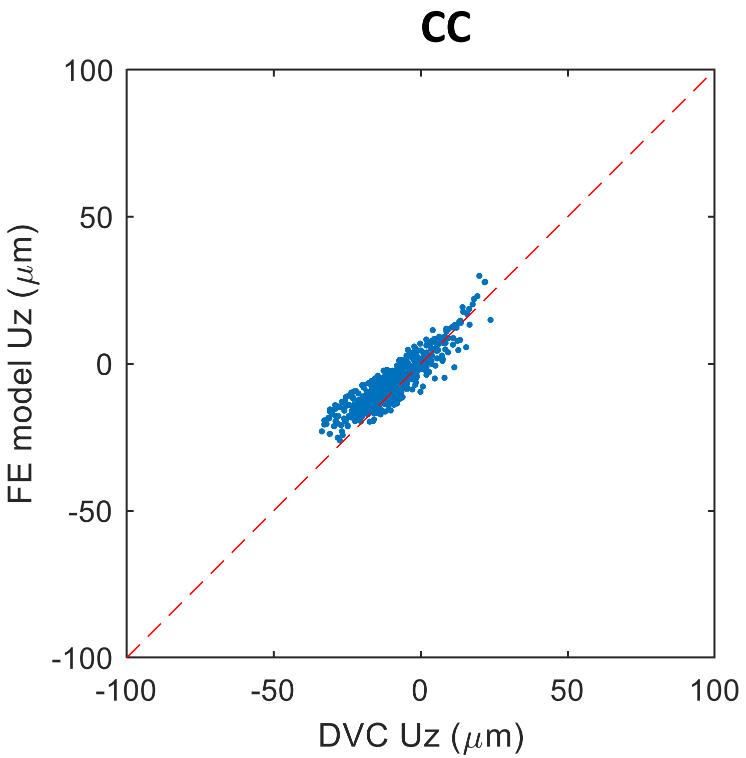

y = 0,75 x + 1,29
R^2 = 0,85
RMSE = 3,6 µm
RMSE% = 7 %

Spatial distribution of the errors

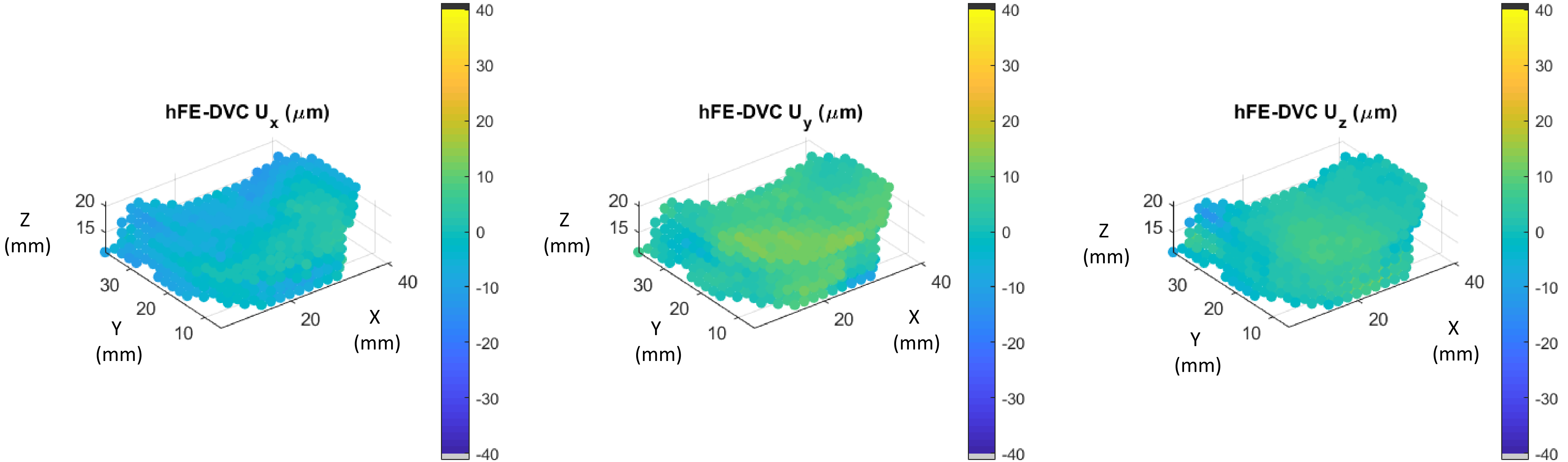

Qualitative comparison of the strains

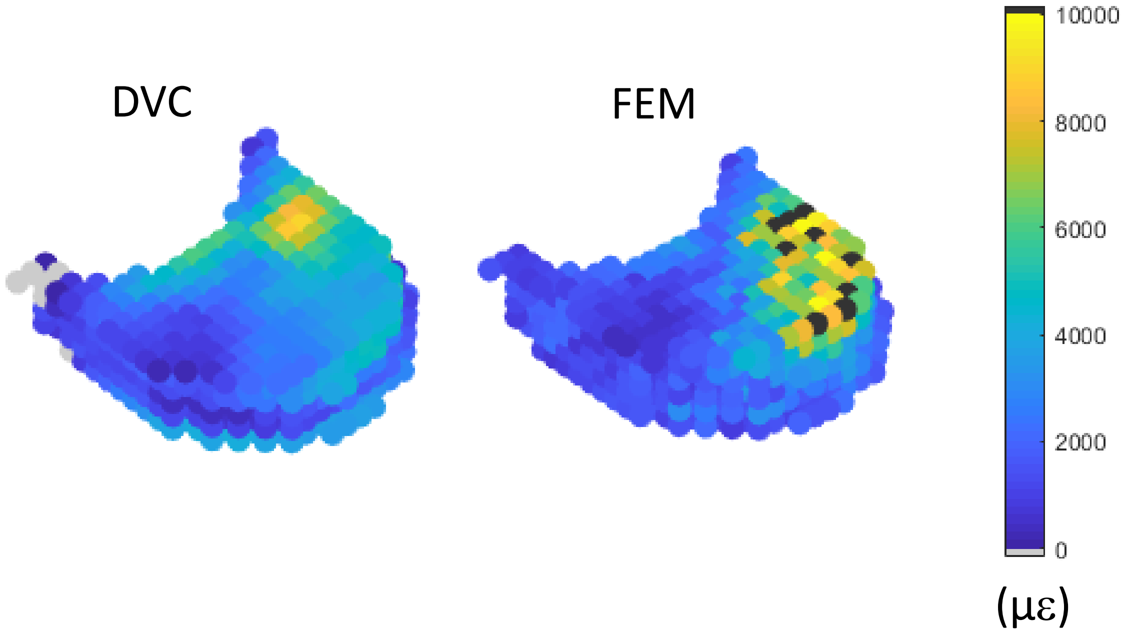

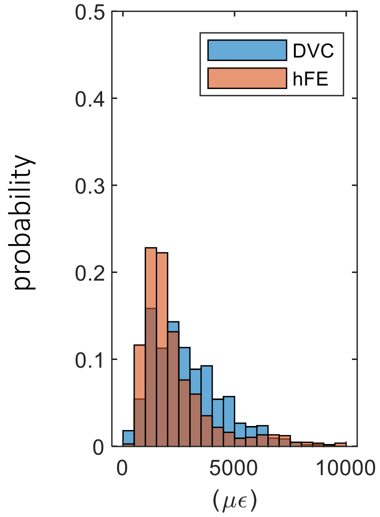

Maximum principal strains

Minimum principal strains

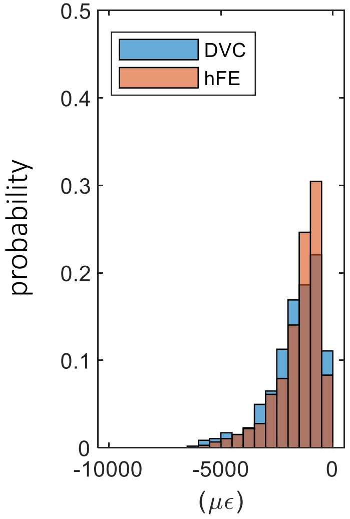

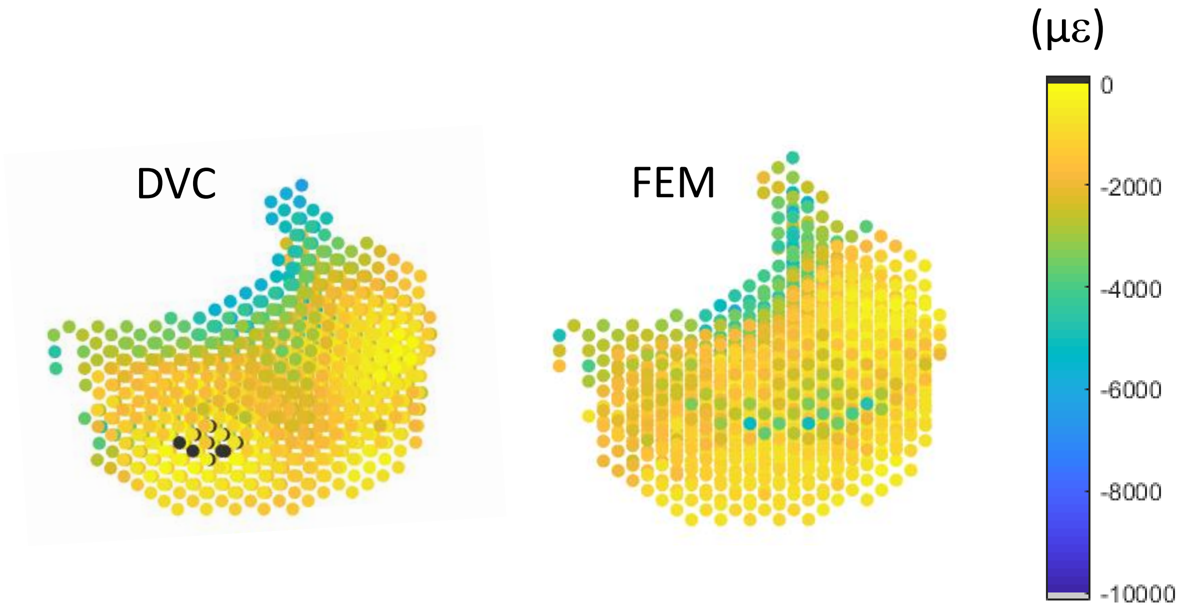

11 - Specimen 780 t6

- Fracture in the other vertebra at the failure step
- Metastatic vertebra (mixed)

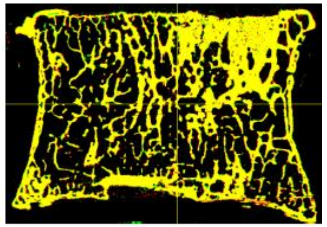

Displacements Correlations

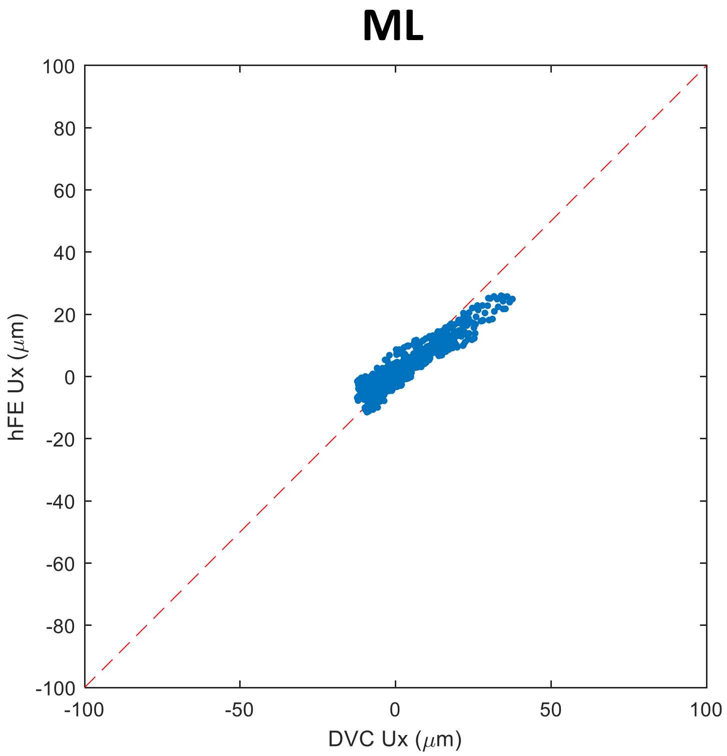

y = 0,60 x + 1,76
R^2 = 0,89
RMSE = 2 µm
RMSE% = 6 %

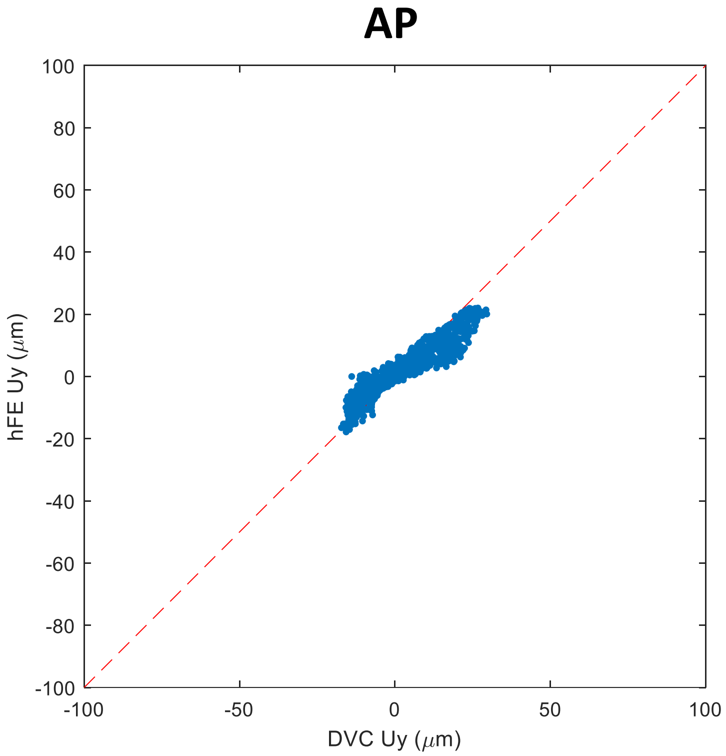

y = 0,65 x + 0,92
R^2 = 0,86
RMSE = 3 µm
RMSE% = 11 %

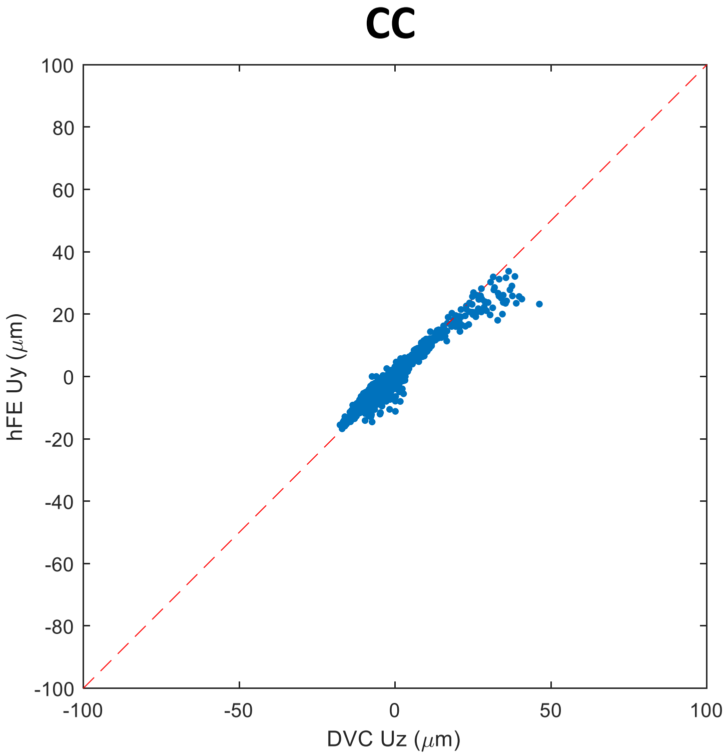

y = 0,84 x + 0,39
R^2 = 0,95
RMSE = 2 µm
RMSE% = 5 %

Spatial distribution of the errors

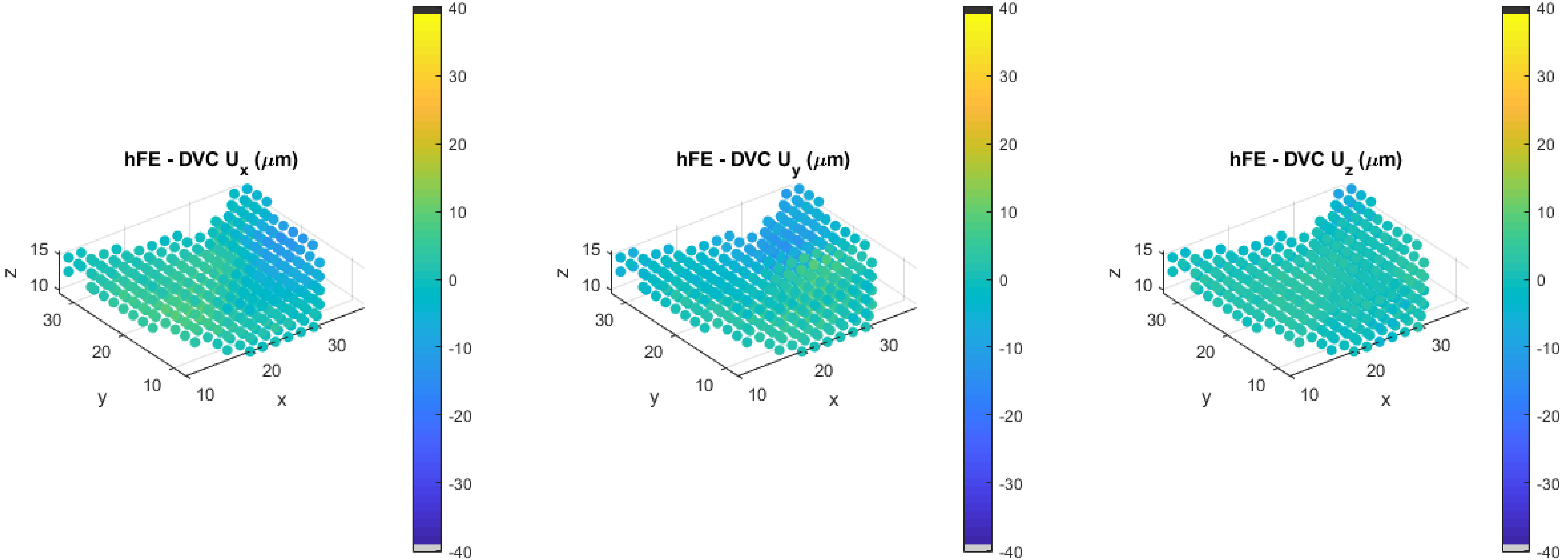

Qualitative comparison of the strains

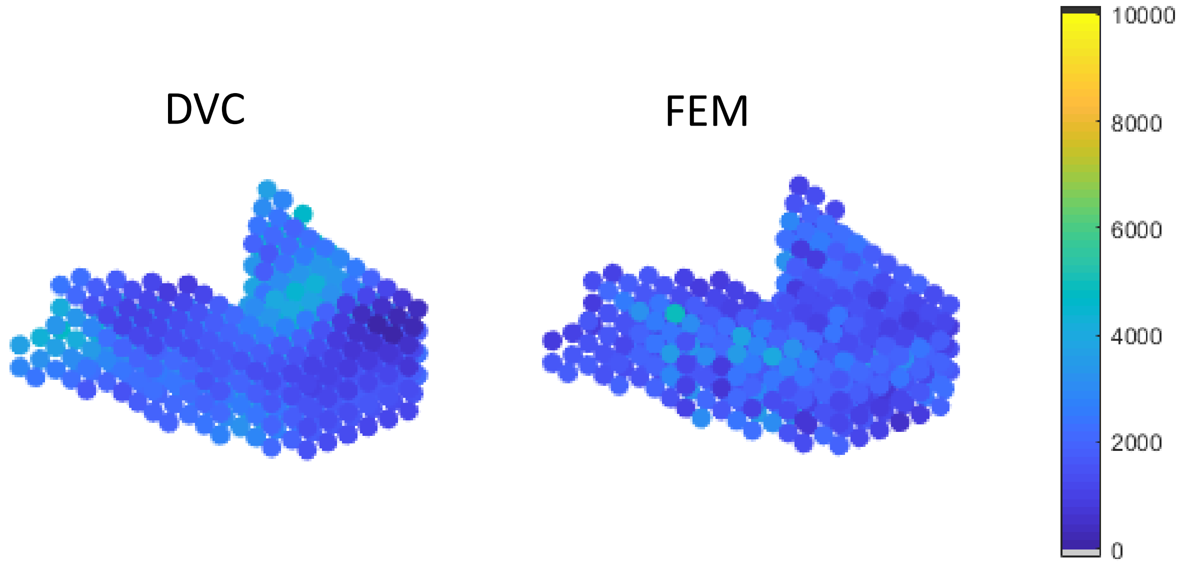

(με)

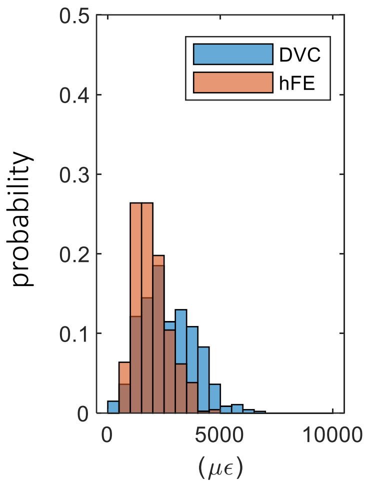

Maximum principal strains

Minimum principal strains

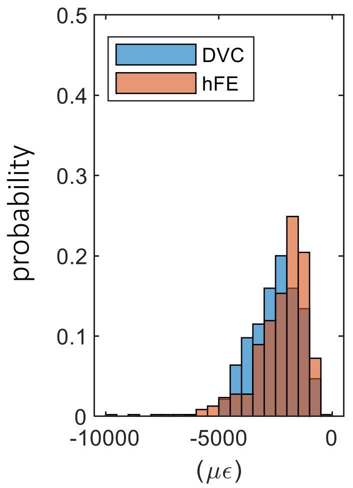

(με)

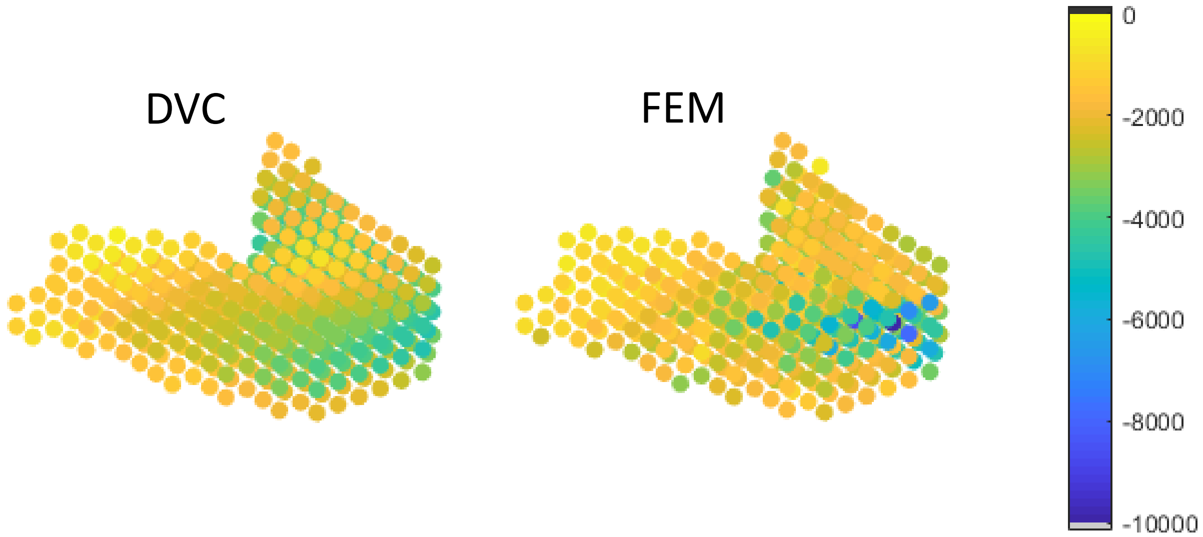

12 - Specimen 780 t7

- Fracture in this vertebra at the failure step
- Control vertebra

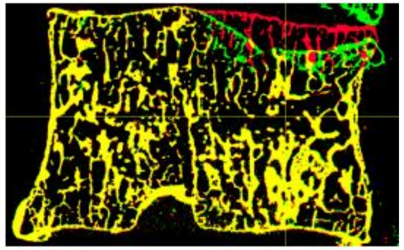

Displacements Correlations

ML

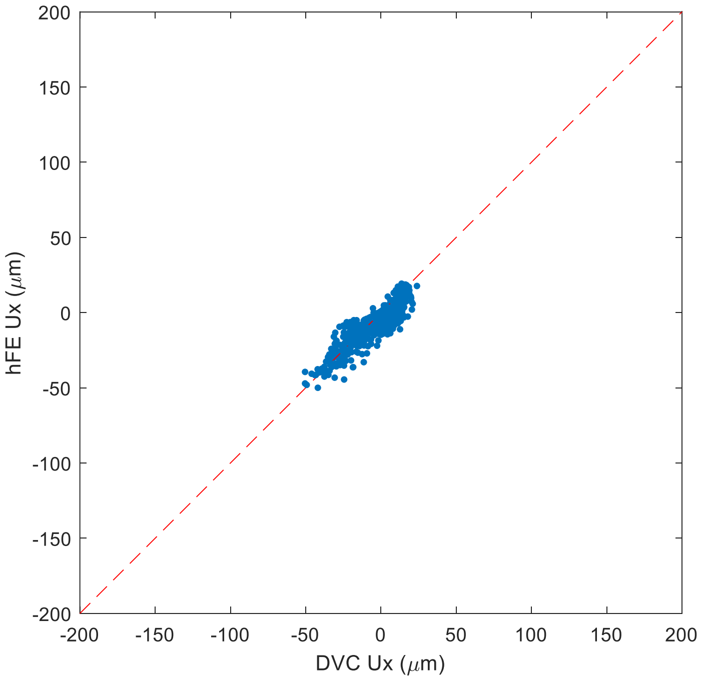

$y = 0{,}73\,x – 4{,}82$
$R^2 = 0{,}78$
RMSE = 5 μm
RMSE% = 12 %

AP

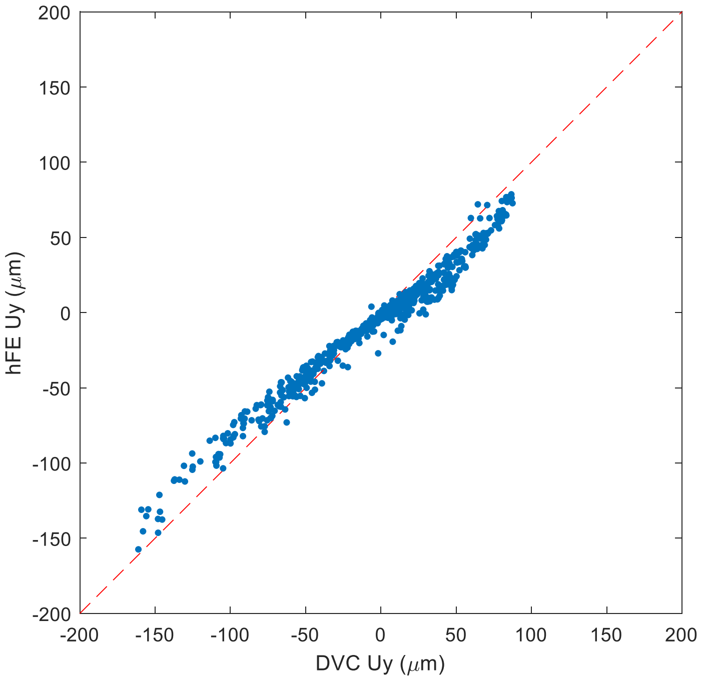

$y = 0{,}77\,x – 4{,}34$
$R^2 = 0{,}98$
RMSE = 5 μm
RMSE% = 3 %

CC

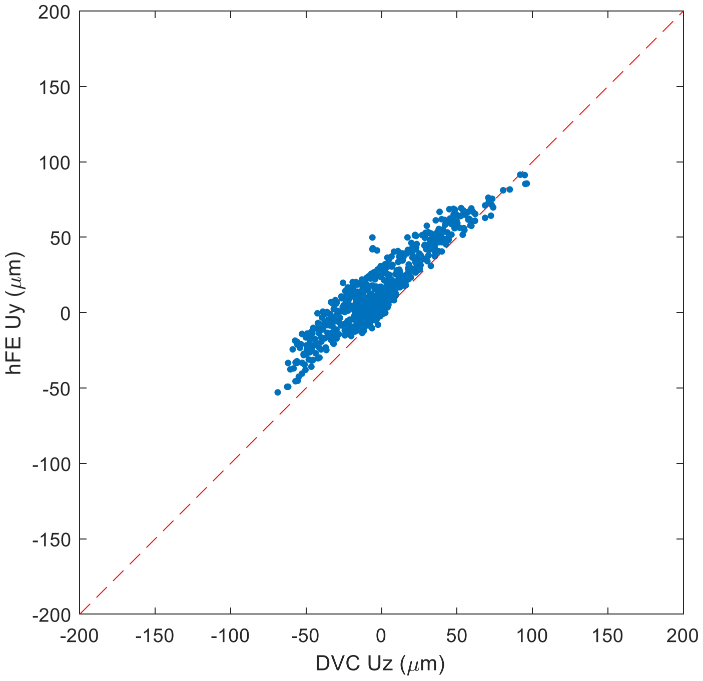

$y = 0{,}82\,x + 15{,}05$
$R^2 = 0{,}82$
RMSE = 9 μm
RMSE% = 13 %

Spatial distribution of the errors

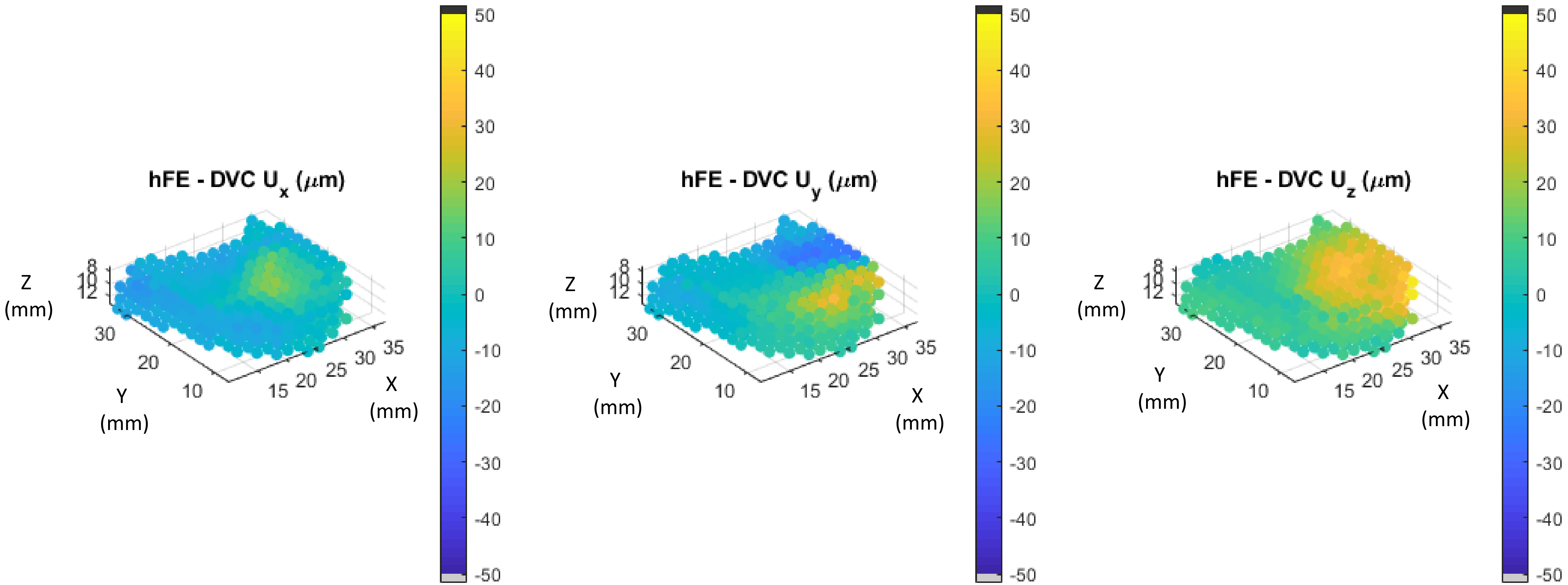

Qualitative comparison of the strains

DVC FEM

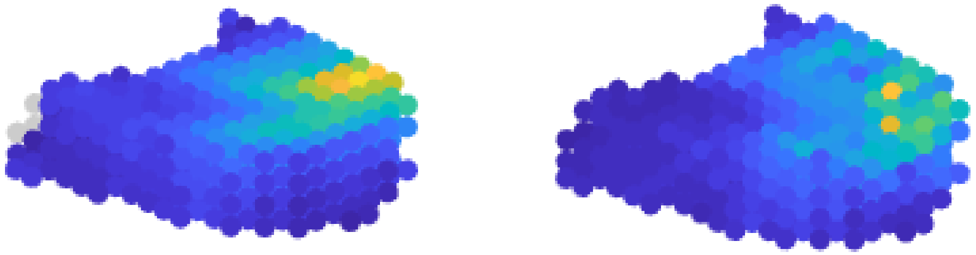

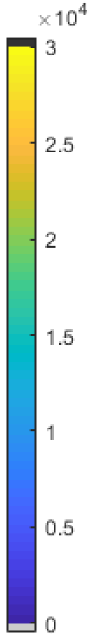

(με)

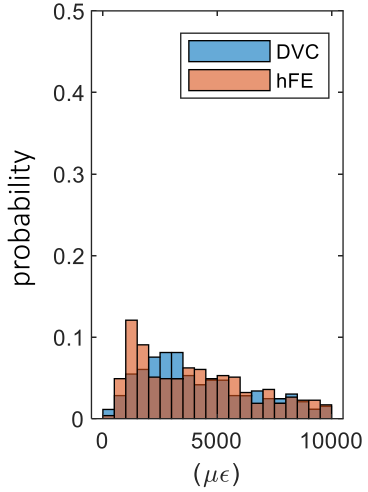

Maximum principal strains

(με)

Minimum principal strains

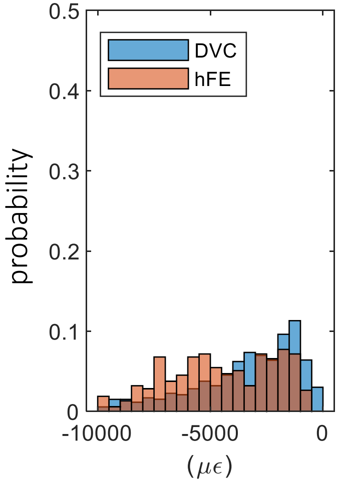

DVC FEM

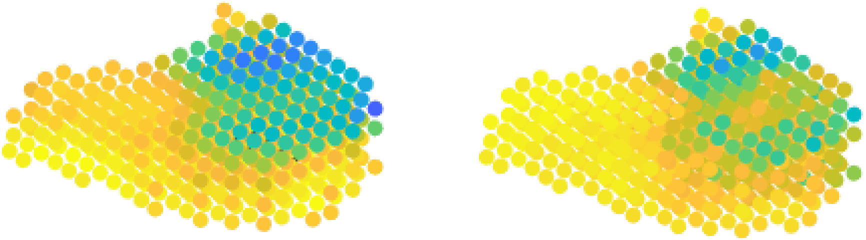

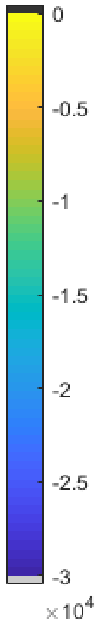

13 - Specimen 780 t12

- Fracture in this vertebra at the failure step
- Metastatic vertebra (mixed)

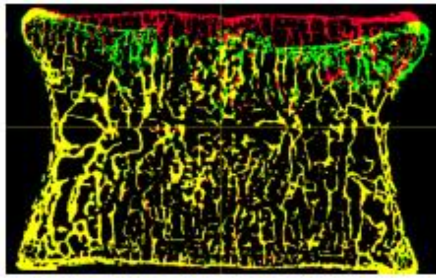

Displacements Correlations

ML

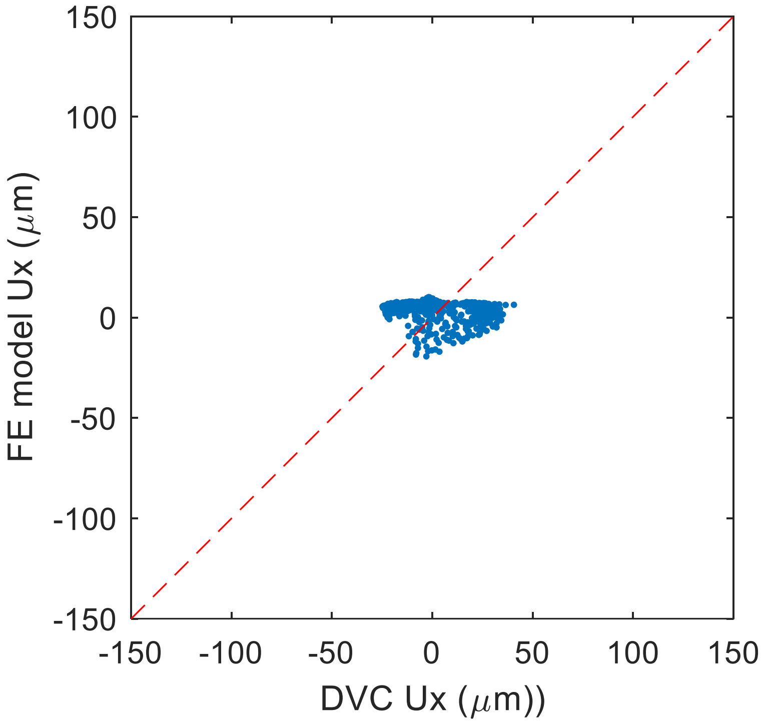

$y = 0{,}05\ x + 2{,}87$
$R^2 = 0{,}02$
RMSE = 6,4 μm
RMSE% = 16 %

AP

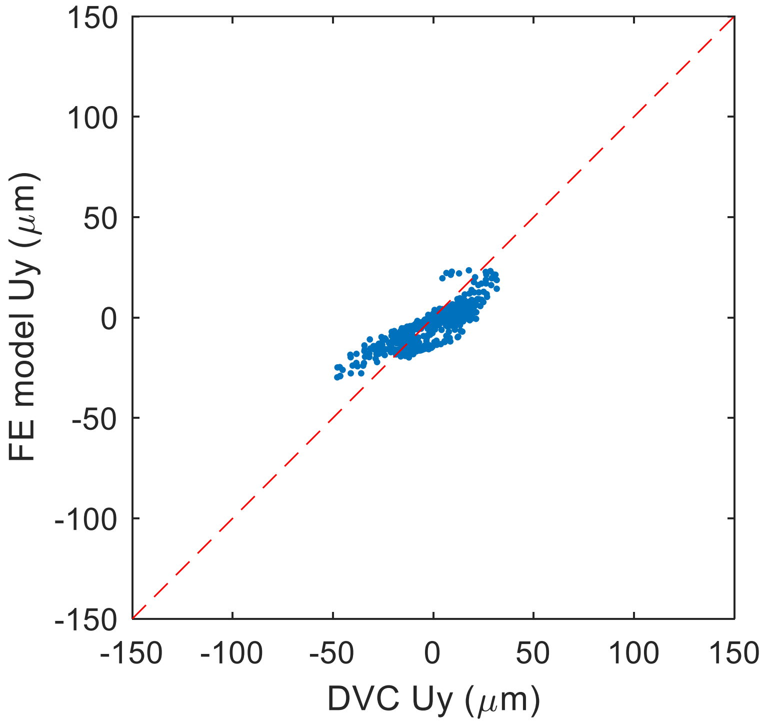

$y = 0{,}70\ x – 3{,}57$
$R^2 = 0{,}78$
RMSE = 7,0 μm
RMSE% = 11 %

CC

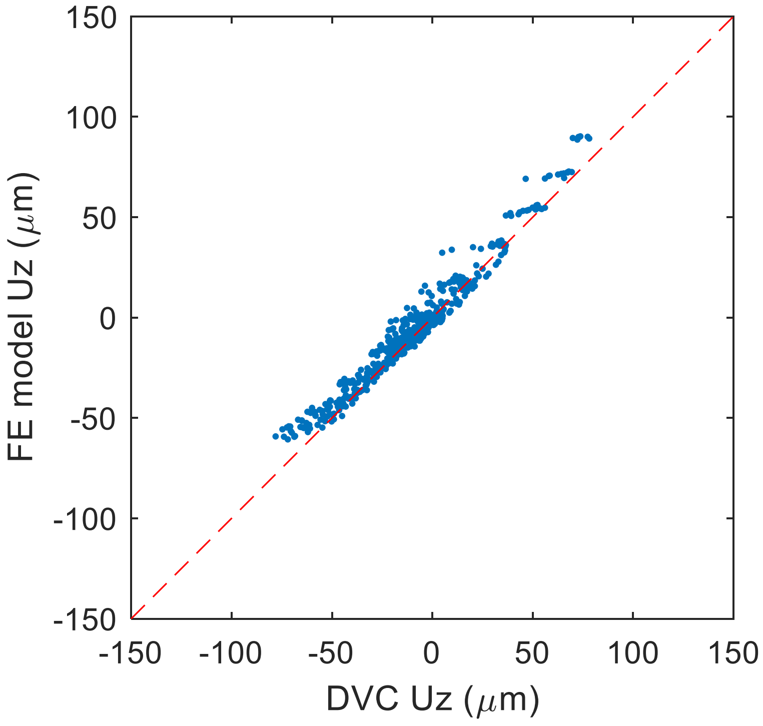

$y = 0{,}91\ x + 5{,}23$
$R^2 = 0{,}90$
RMSE = 8,6 μm
RMSE% = 9 %

Spatial distribution of the errors

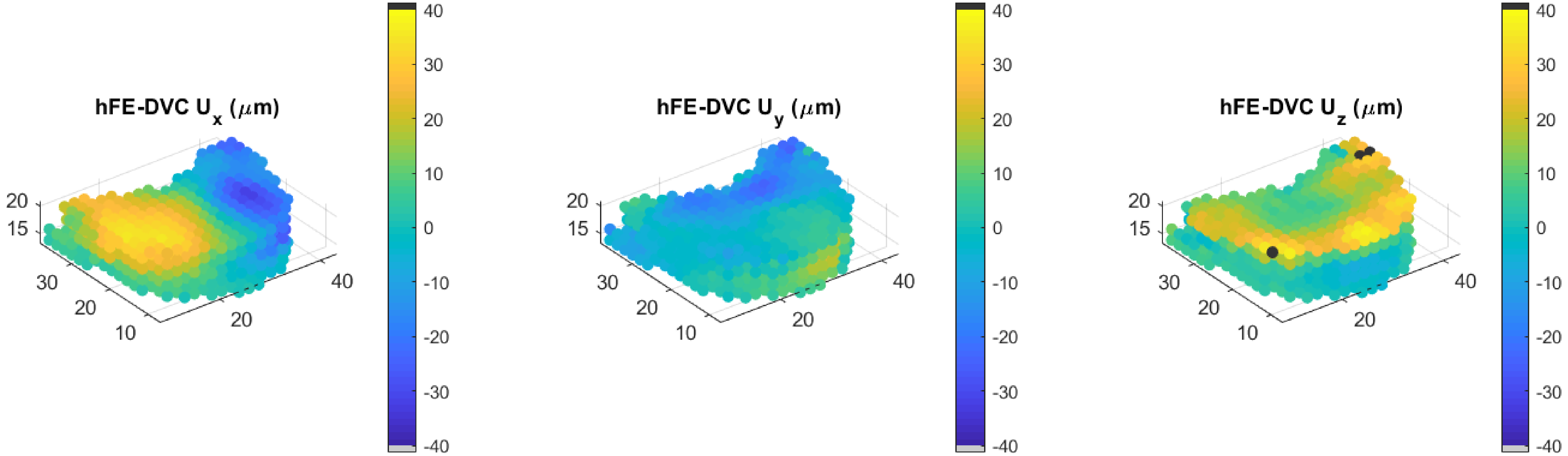

Qualitative comparison of the strains

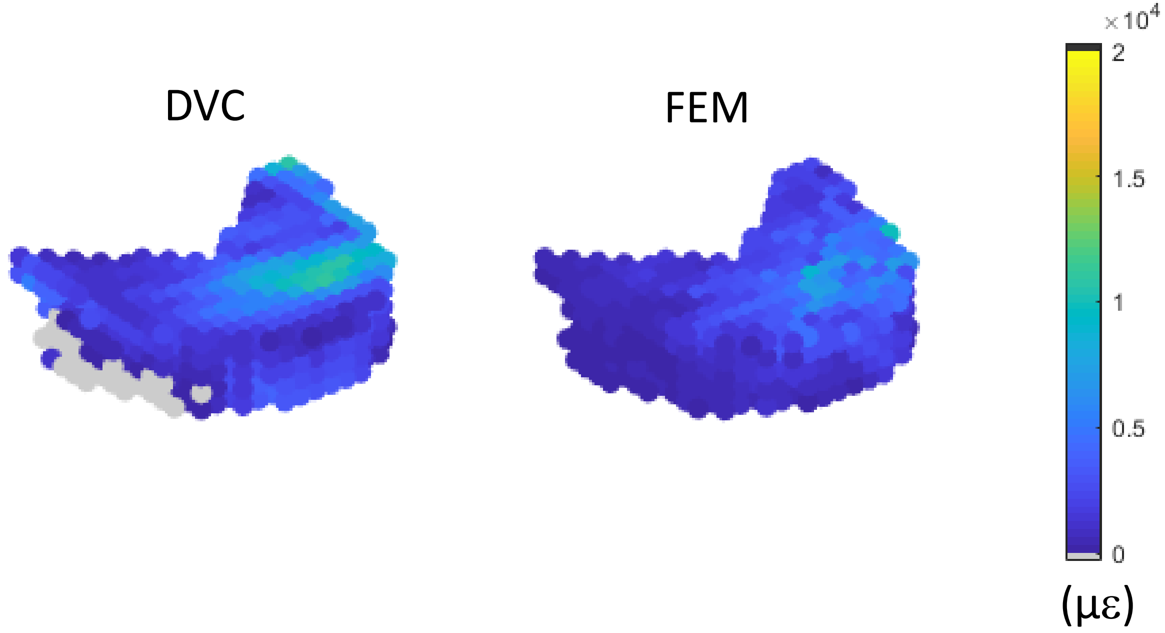

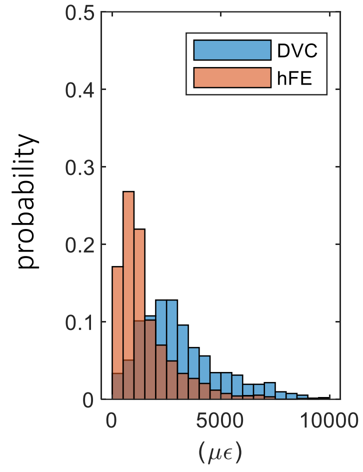

Maximum principal strains

Minimum principal strains

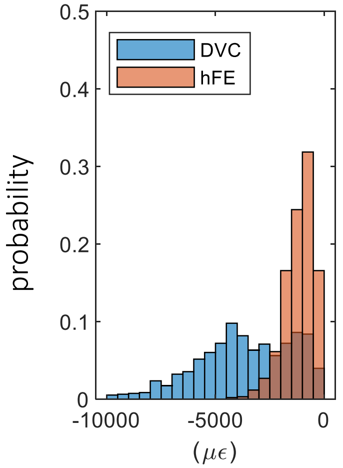

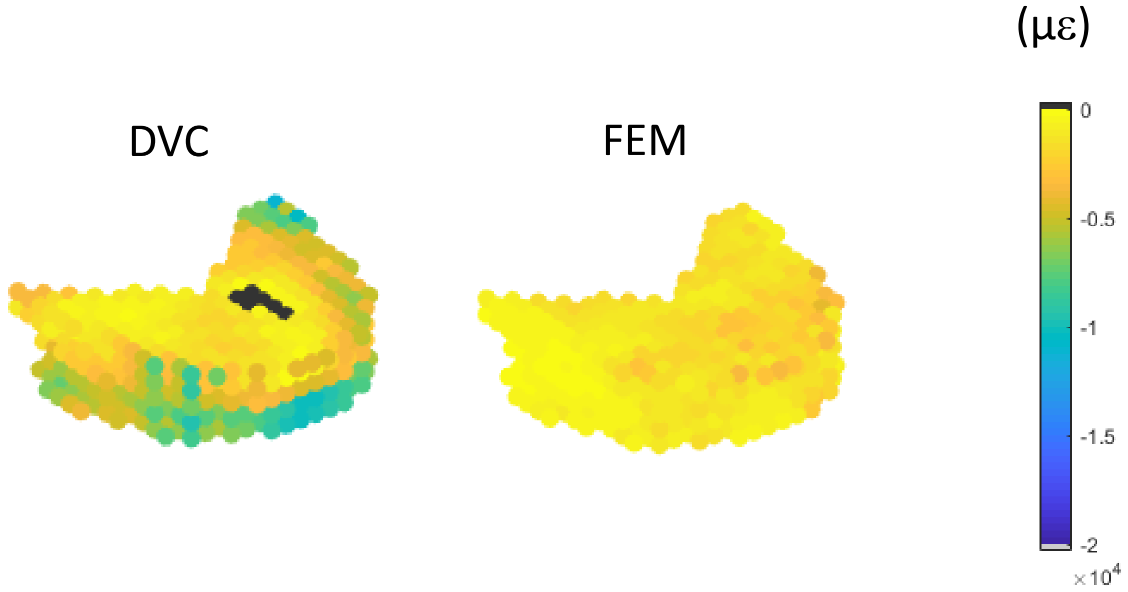

14 - Specimen 780 L1

- Fracture in the other vertebra at the failure step
- Control vertebra

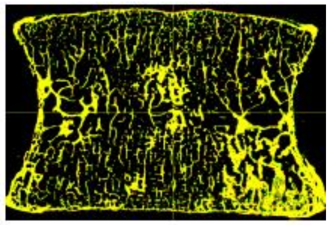

Displacements Correlations

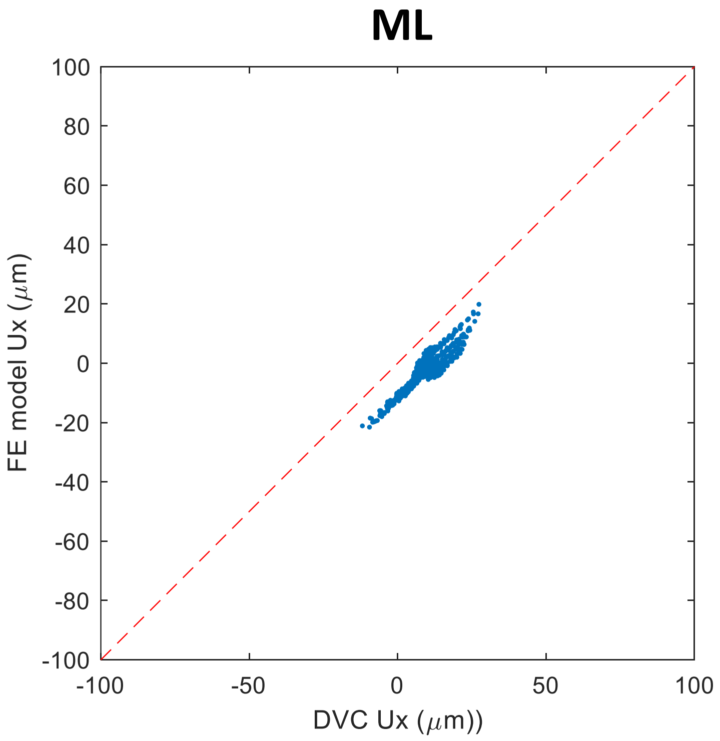

y = 0,82 x – 9,71
R^2 = 0,67
RMSE = 3,48 μm
RMSE% = 12 %

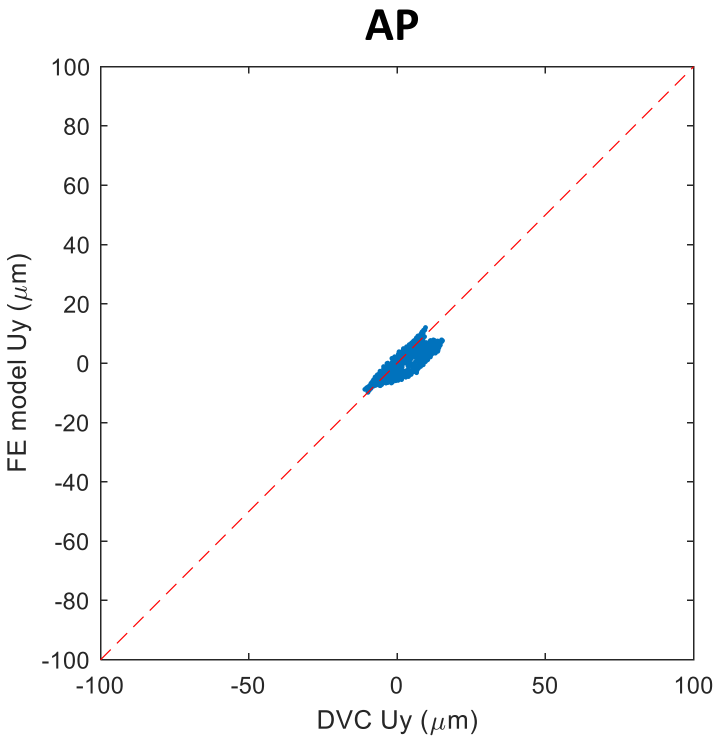

y = 0,72 x – 0,97
R^2 = 0,73
RMSE = 2,82 μm
RMSE% = 17 %

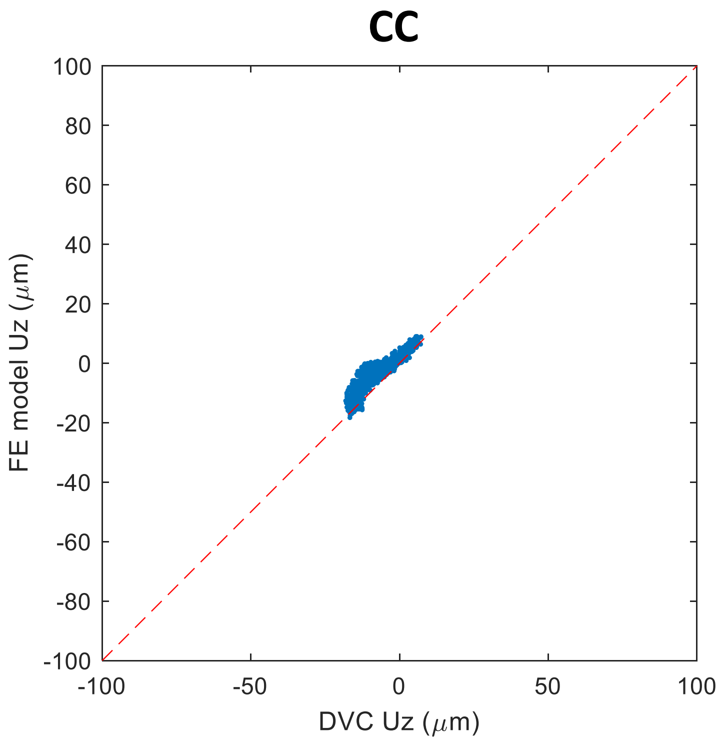

y = 0,95 x + 2,56
R^2 = 0,84
RMSE = 2,92 μm
RMSE% = 15 %

Spatial distribution of the errors

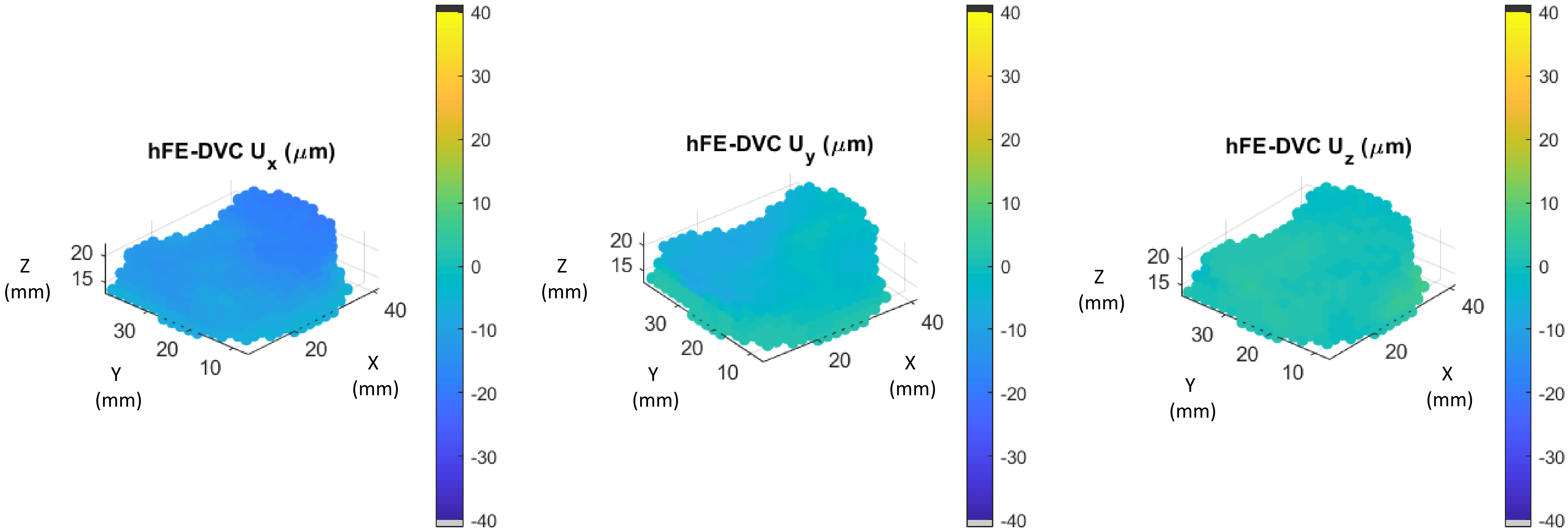

Qualitative comparison of the strains

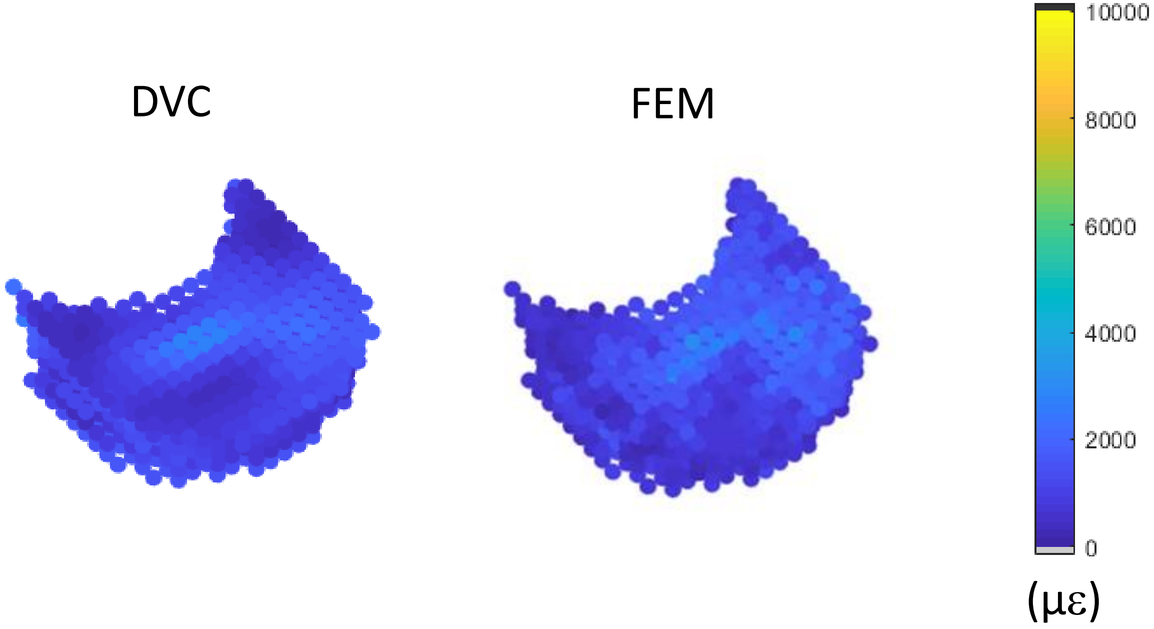

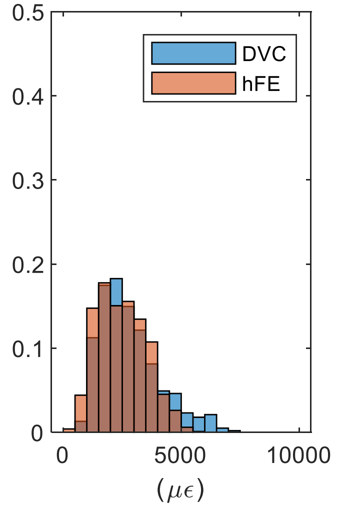

Maximum principal strains

Minimum principal strains

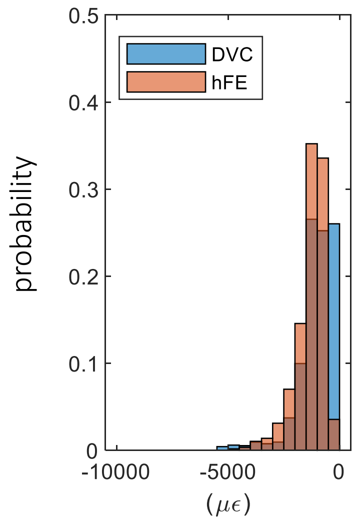

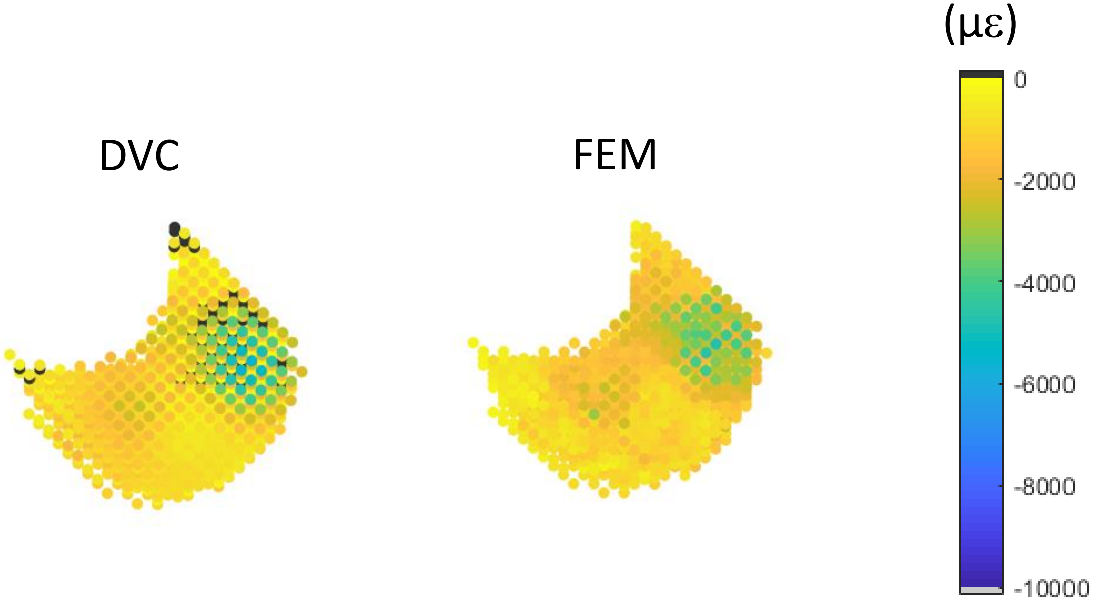

15 - Specimen 769 t11

- Fracture in the other vertebra at the failure step
- Metastatic vertebra (lytic)

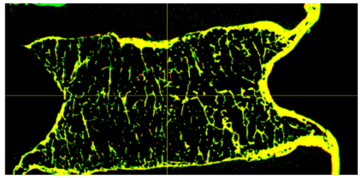

Displacements Correlations

ML

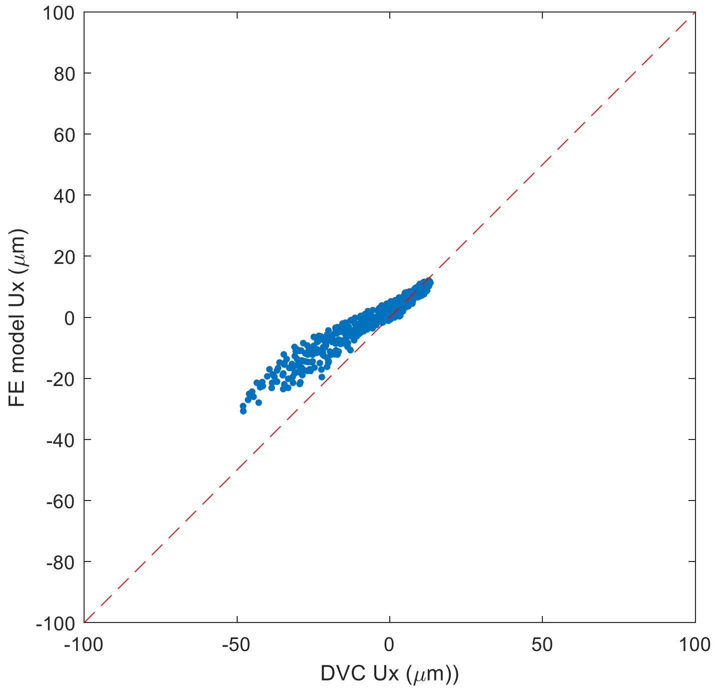

y = 0,59 x + 2,46
R^2 = 0,93
RMSE = 2 µm
RMSE% = 5 %

AP

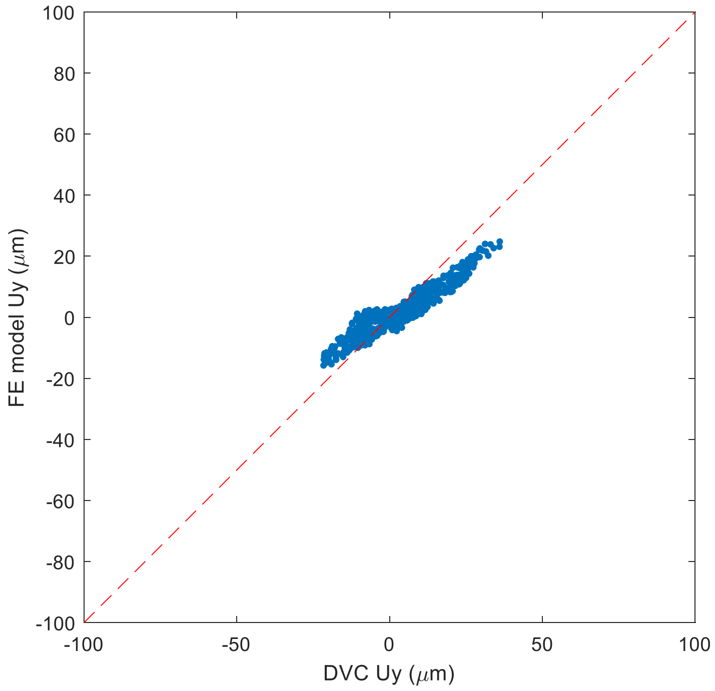

y = 0,59 x – 0,06
R^2 = 0,88
RMSE = 3 µm
RMSE% = 7 %

CC

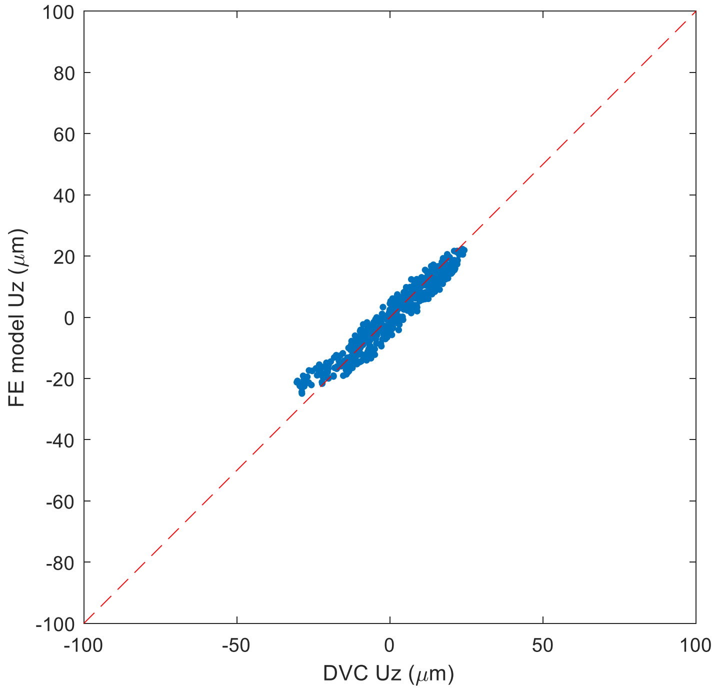

y = 0,87 x + 0,08
R^2 = 0,94
RMSE = 3 µm
RMSE% = 9 %

Spatial distribution of the errors

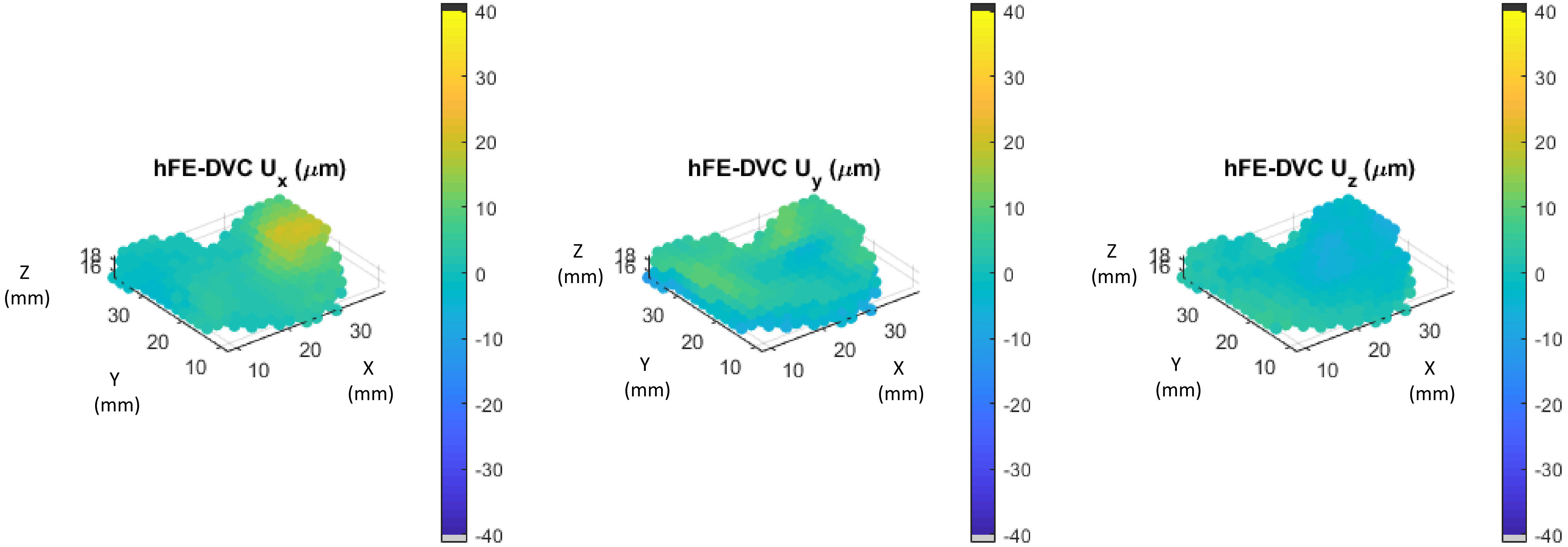

Qualitative comparison of the strains

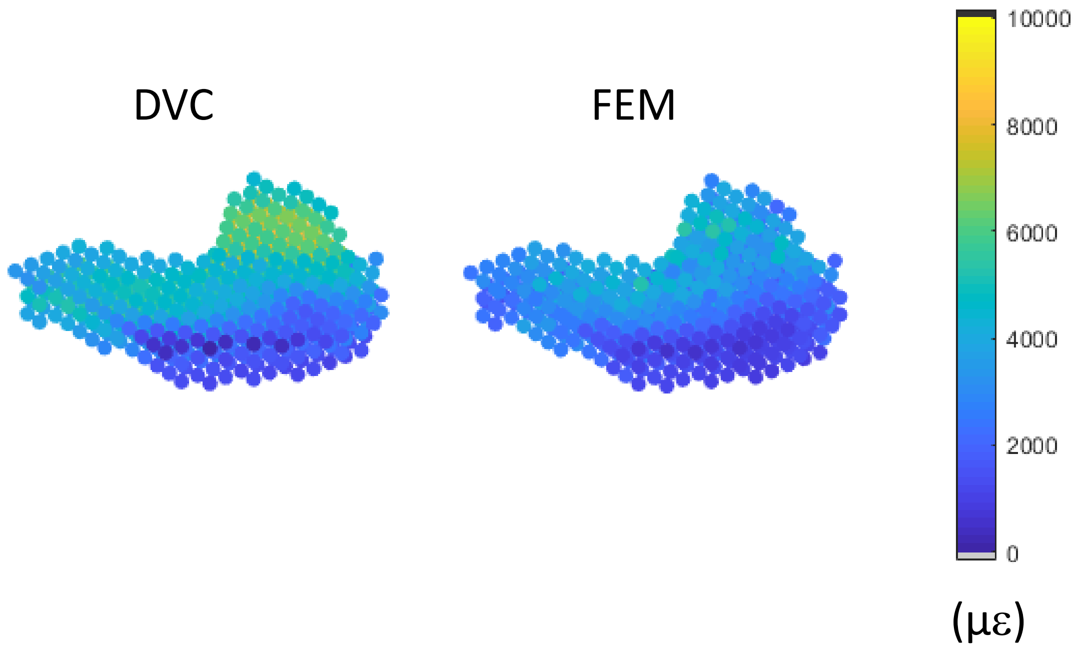

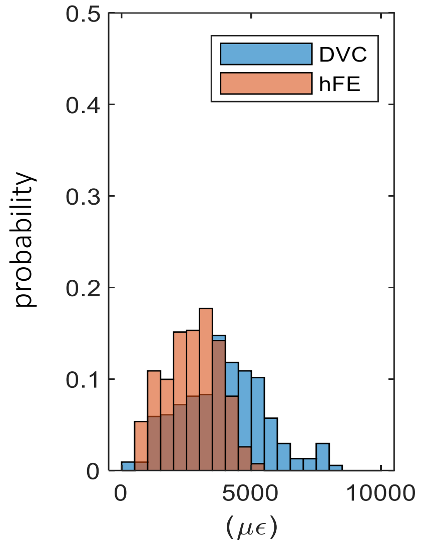

Maximum principal strains

Minimum principal strains

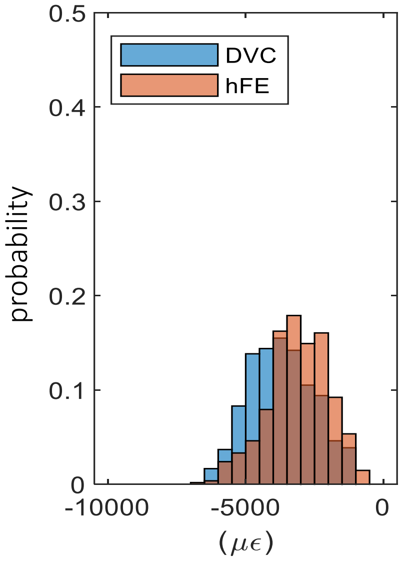

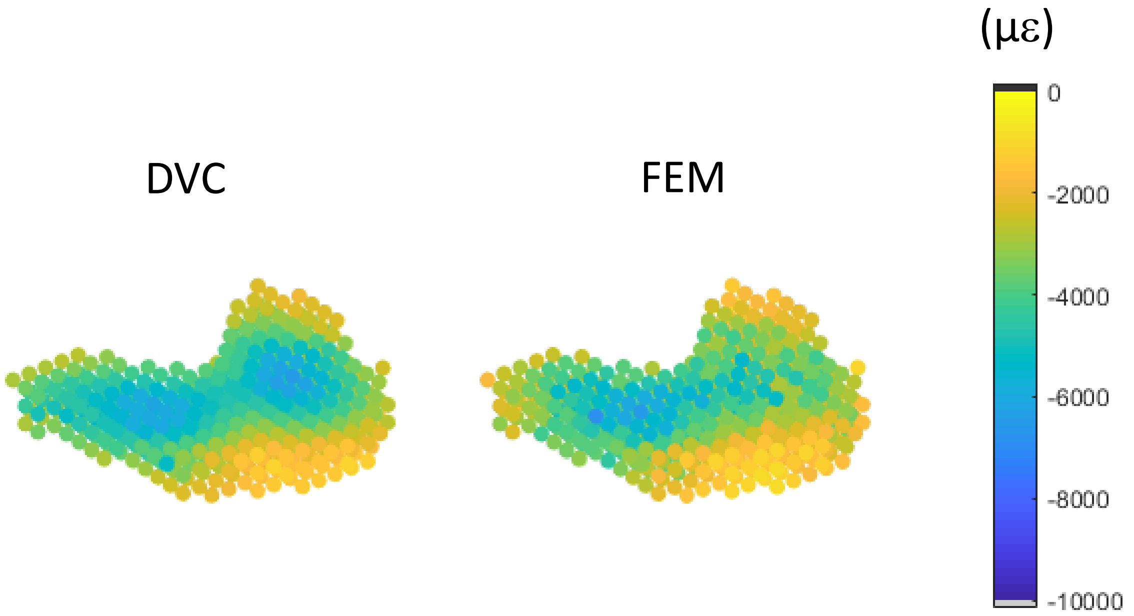

16 - Specimen 781 t11

- Fracture in the other vertebra at the failure step
- Control vertebra

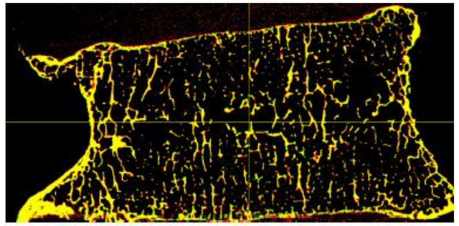

Displacements Correlations

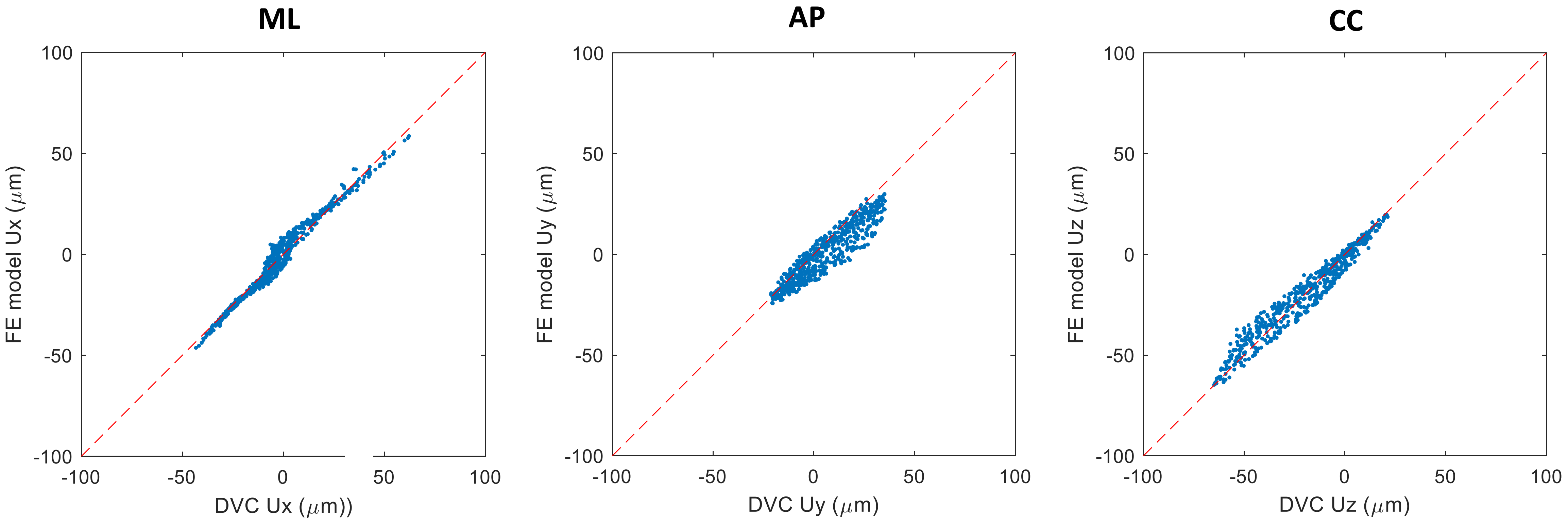

y = 1,01 x – 0,28
R^2 = 0,97
RMSE = 3,2 μm
RMSE% = 5 %

y = 0,85 x – 3,27
R^2 = 0,89
RMSE = 4,7 μm
RMSE% = 12 %

y = 0,92 x – 1,39
R^2 = 0,96
RMSE = 4,4 μm
RMSE% = 5 %

Spatial distribution of the errors

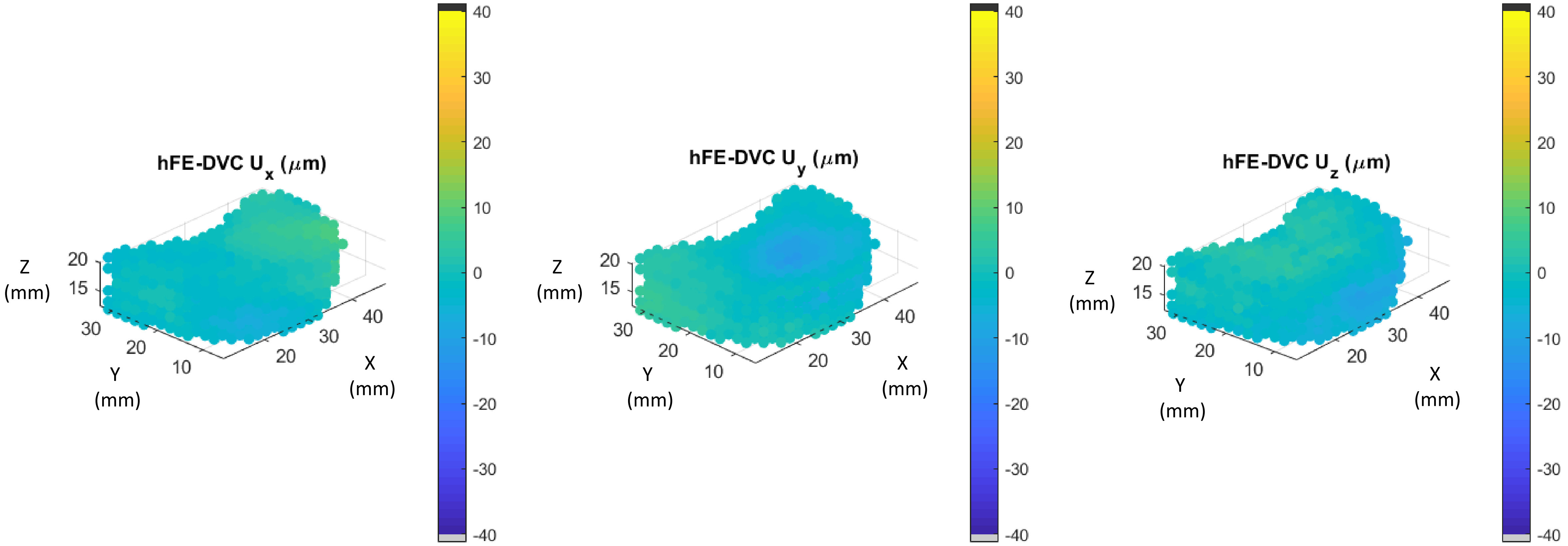

Qualitative comparison of the strains

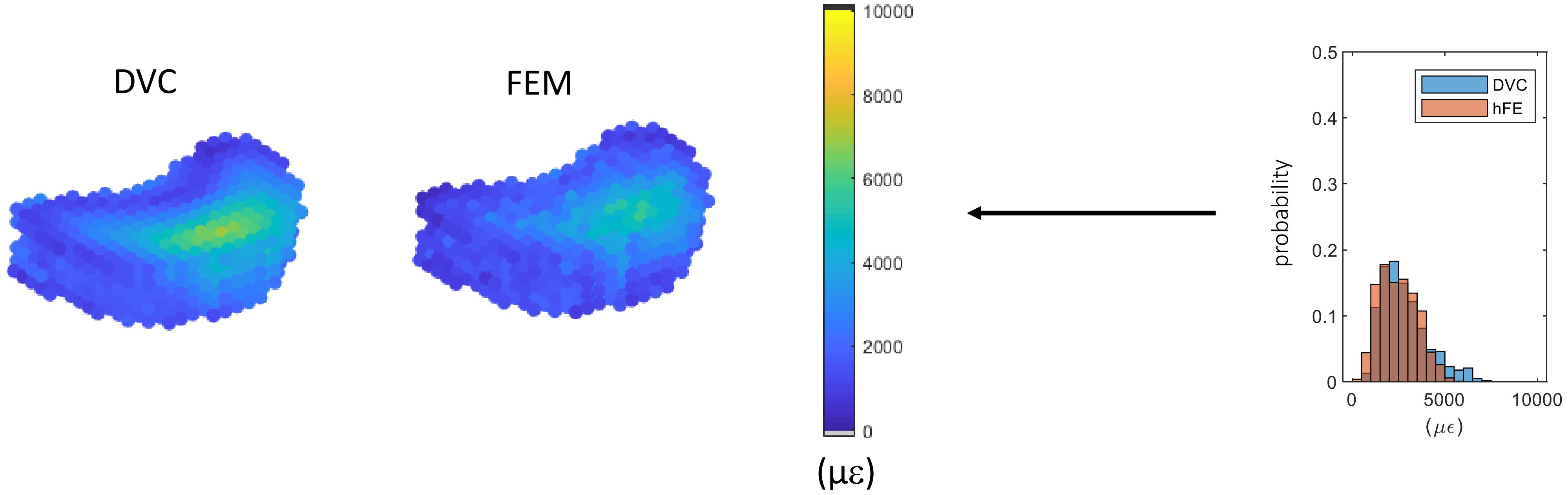

Maximum principal strains

Minimum principal strains

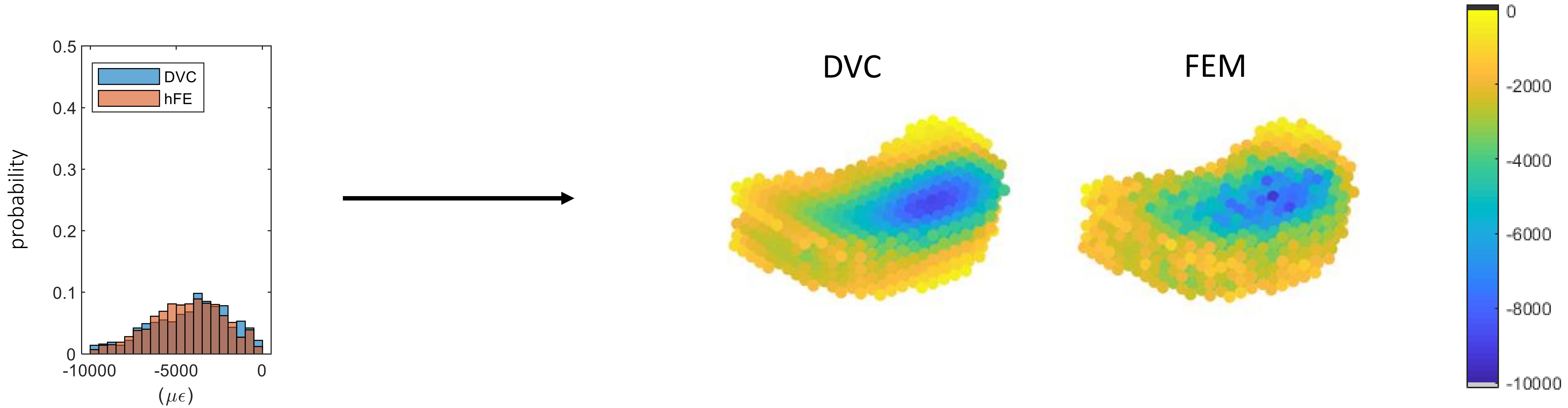

17 - Specimen 782 t5

- Fracture in this vertebra at the failure step
- Metastatic vertebra (lytic)

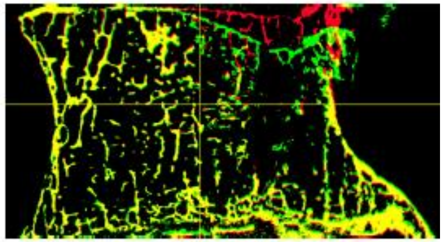

Displacements Correlations

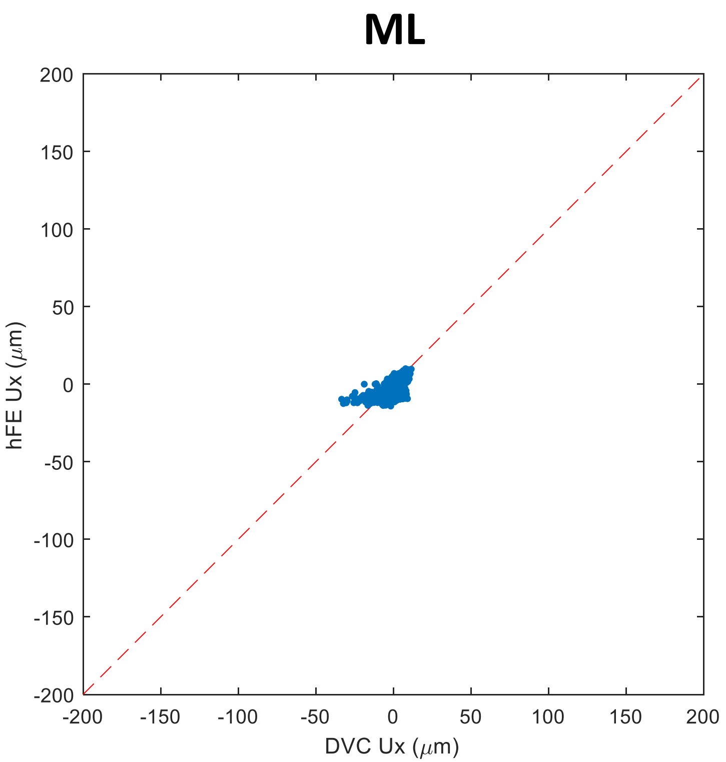

y = 0,32 x -5,48
R^2 = 0,22
RMSE = 4 µm
RMSE% = 17 %

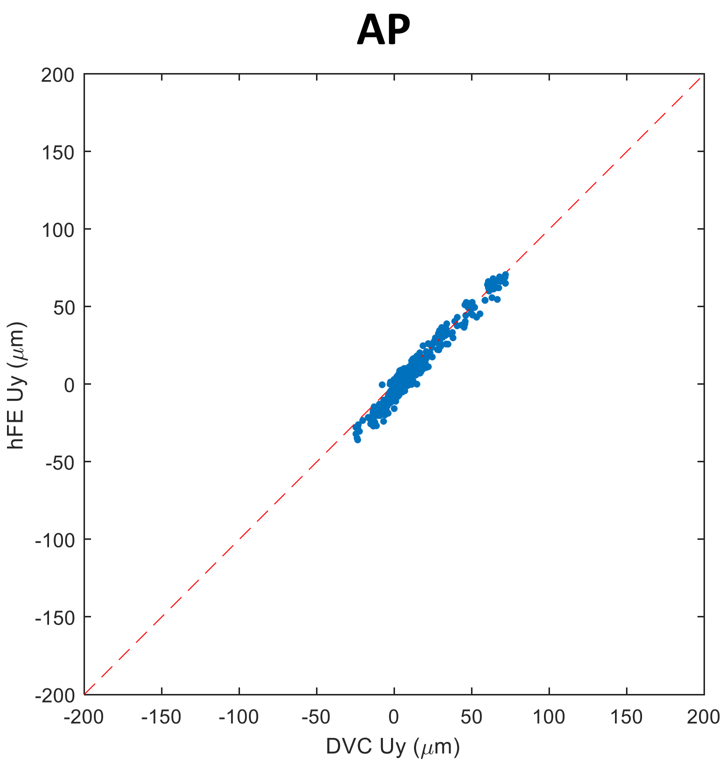

y = 1,06 x – 4,65
R^2 = 0,96
RMSE = 3,54 µm
RMSE% = 5,54 %

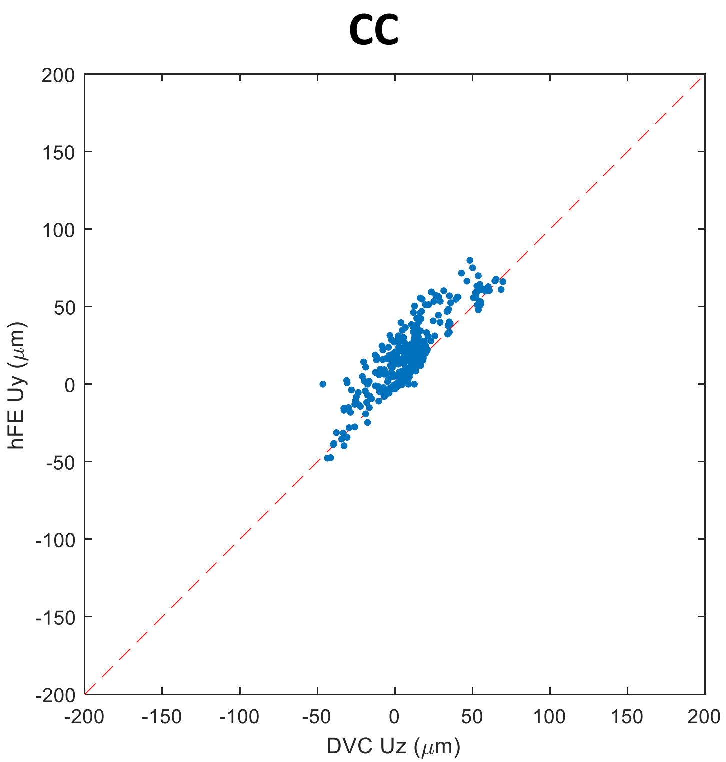

y = 0,81 x + 10,30
R^2 = 0,52
RMSE = 10 µm
RMSE% = 18 %

Spatial distribution of the errors

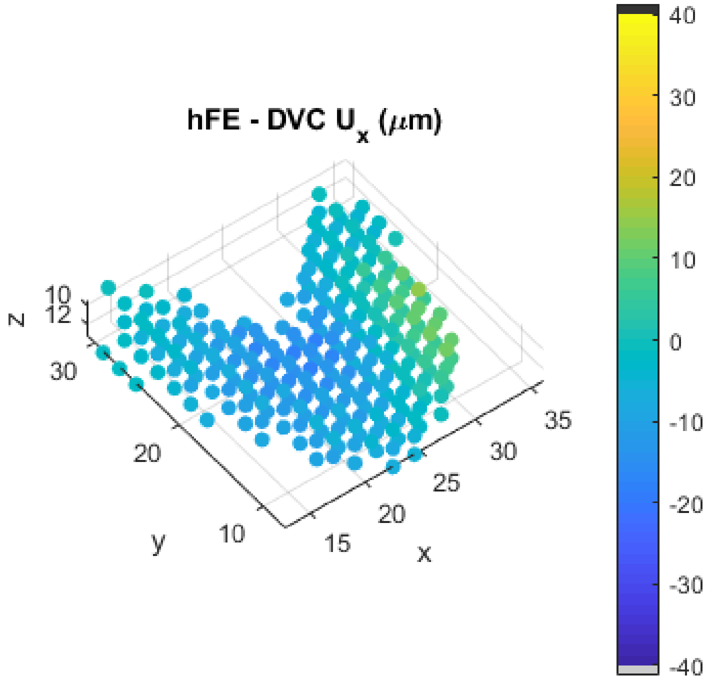

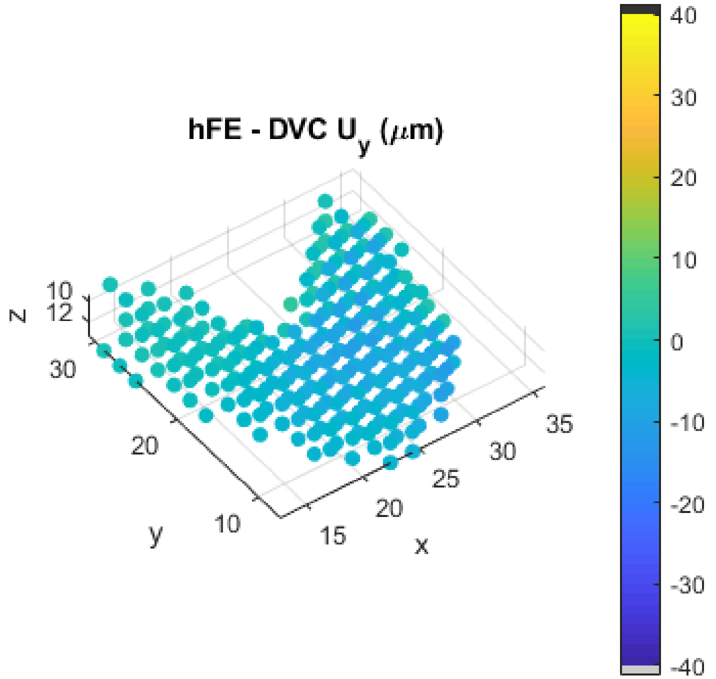

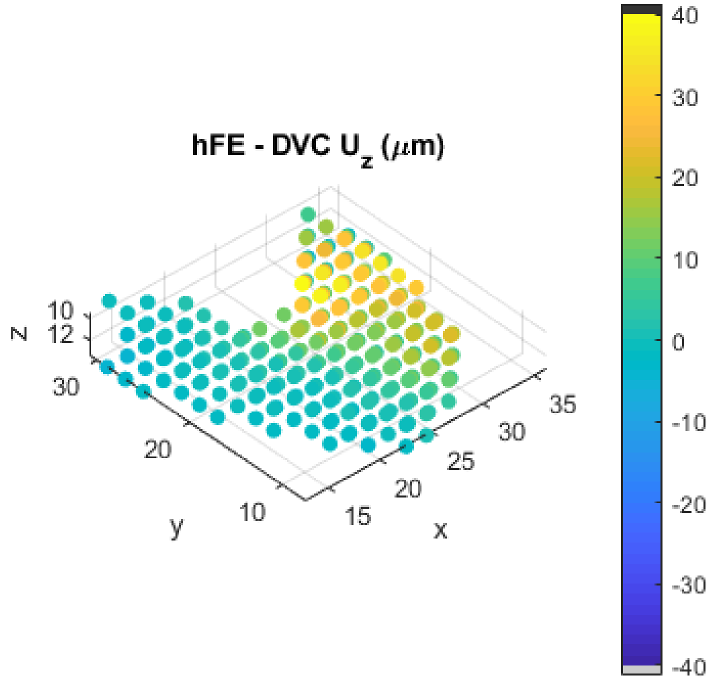

Qualitative comparison of the strains

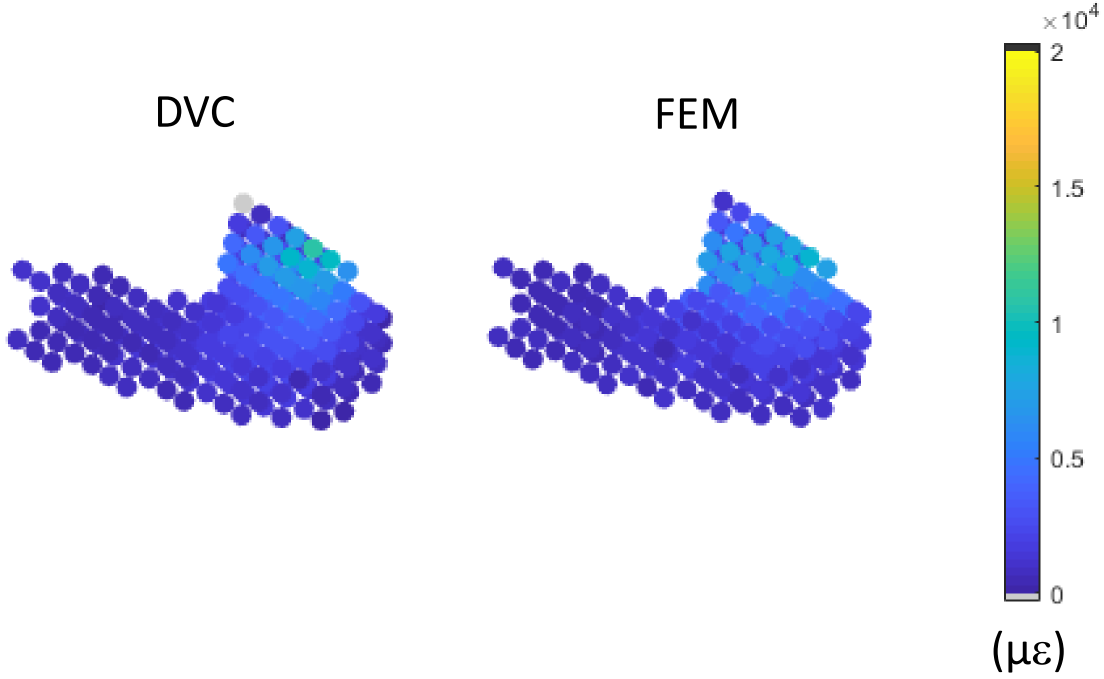

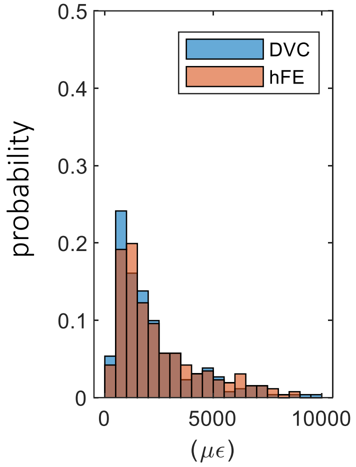

Maximum principal strains

Minimum principal strains

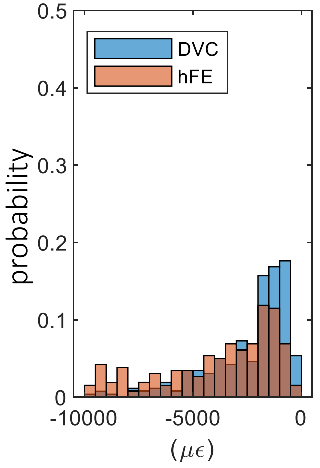

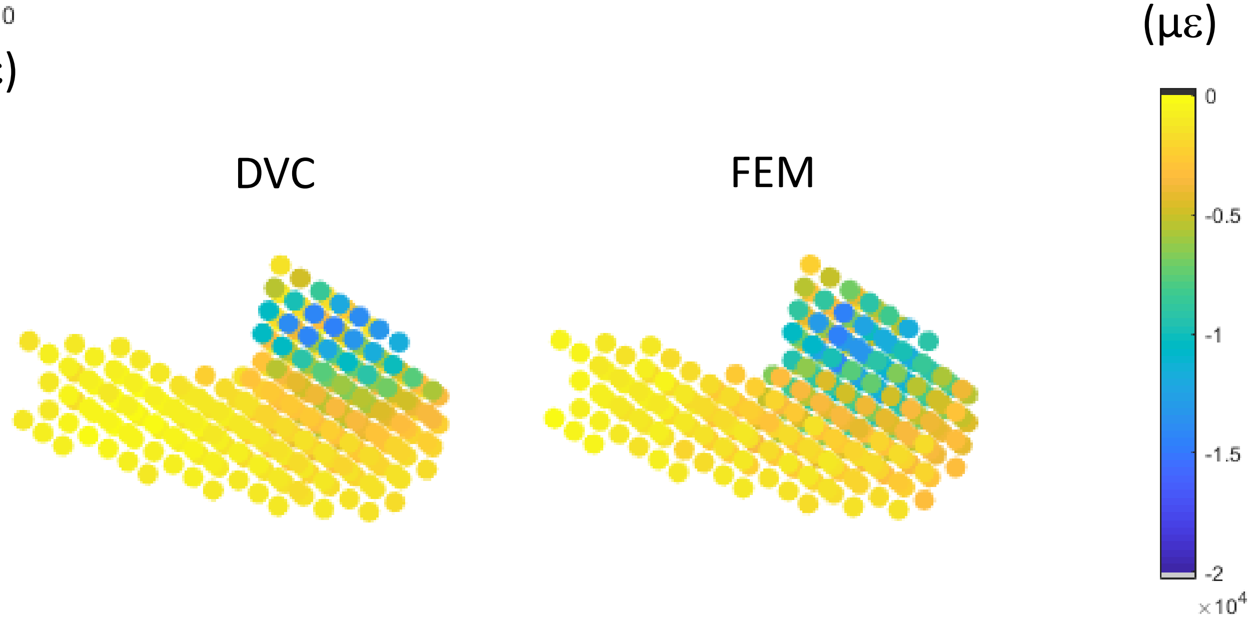

18 - Specimen 785 t7

- Fracture in this vertebra at the failure step
- Control vertebra

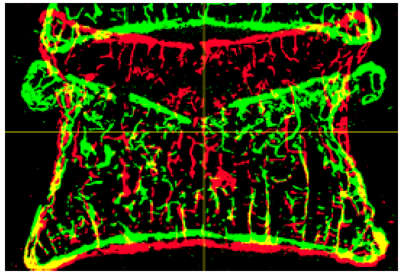

Displacements Correlations

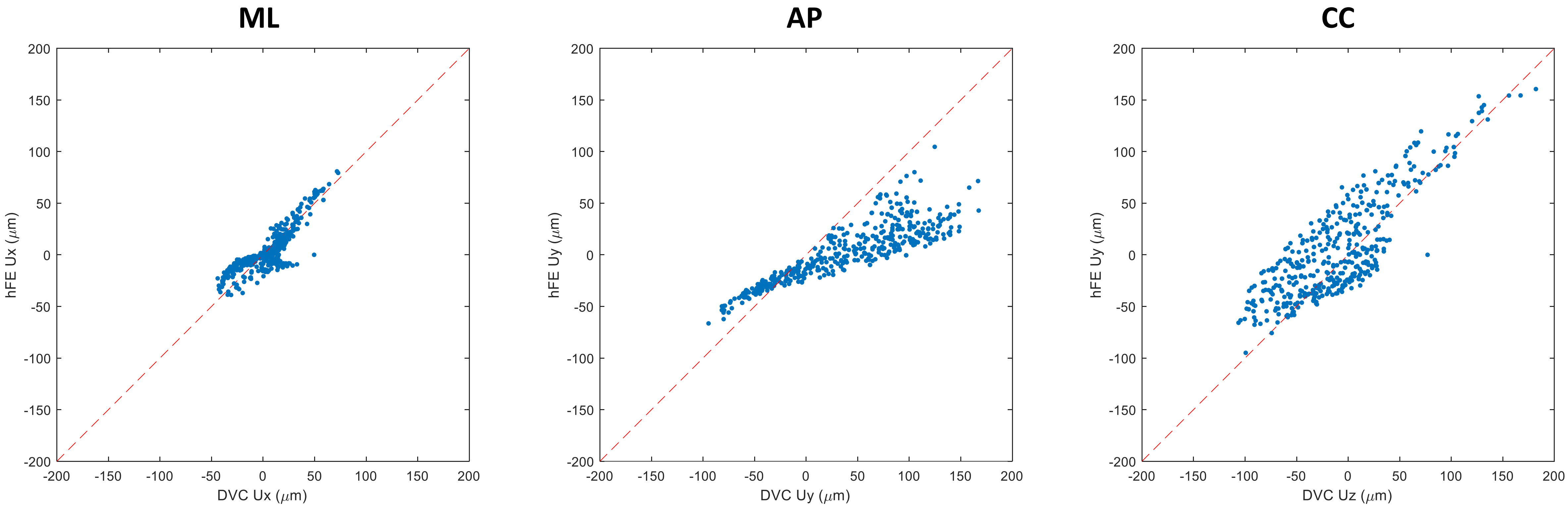

y = 0,70 x + 0,31
R^2 = 0,69
RMSE = 10 µm
RMSE% = 14 %

y = 0,37 x – 14,32
R^2 = 0,81
RMSE = 11 µm
RMSE% = 7 %

y = 0,78 x + 13,57
R^2 = 0,65
RMSE = 27 µm
RMSE% = 17 %

Spatial distribution of the errors on displacements

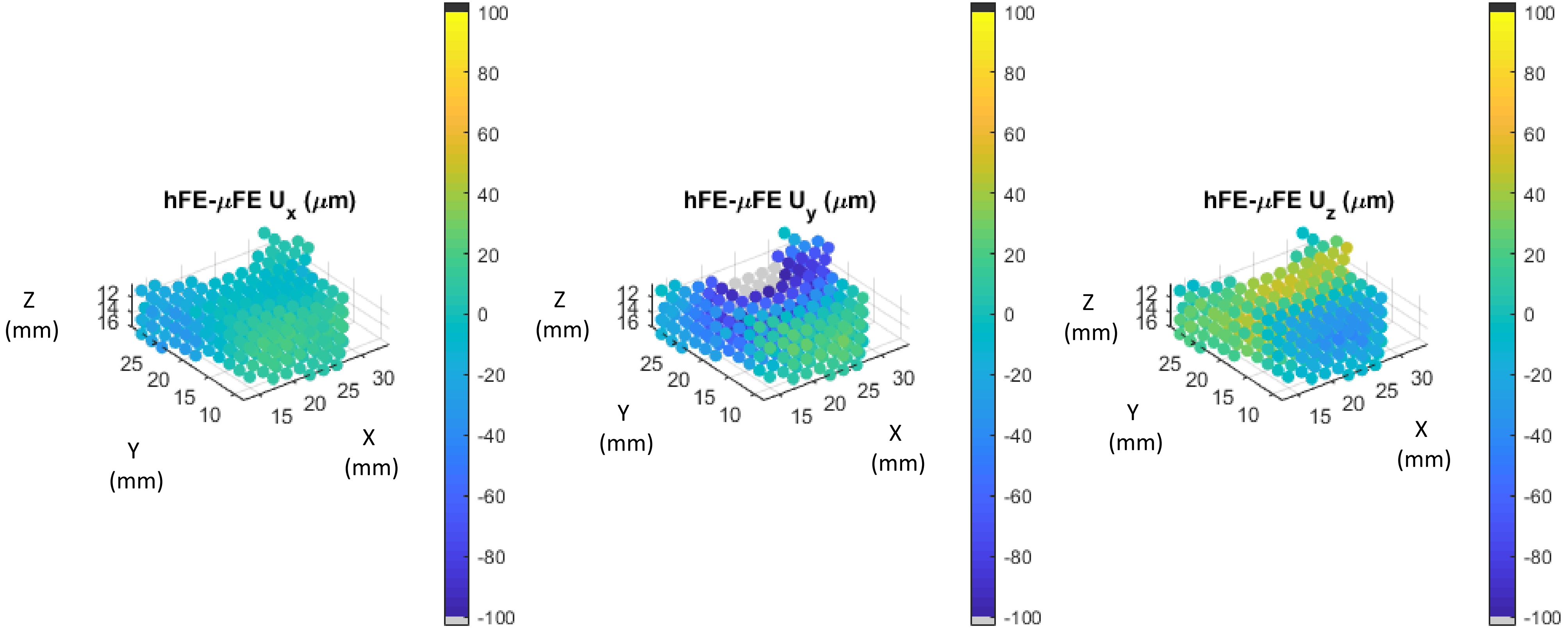

Qualitative comparison of the strains

DVC FEM

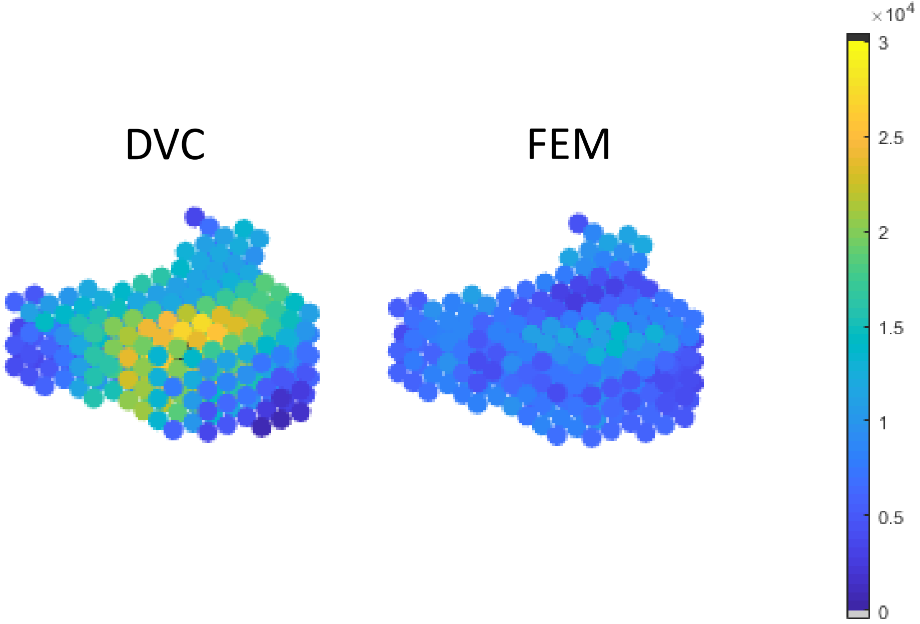

(με)

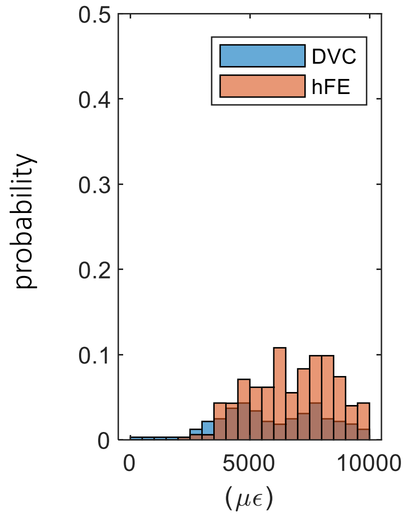

Maximum principal strains

(με)

Minimum principal strains

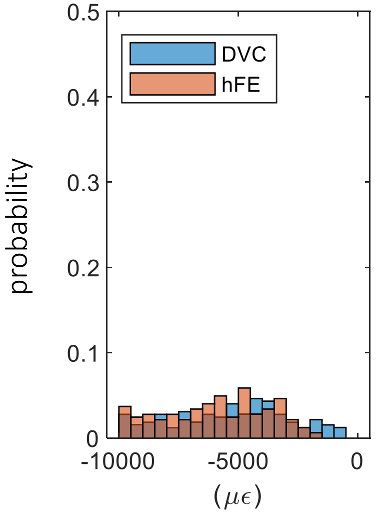

DVC FEM

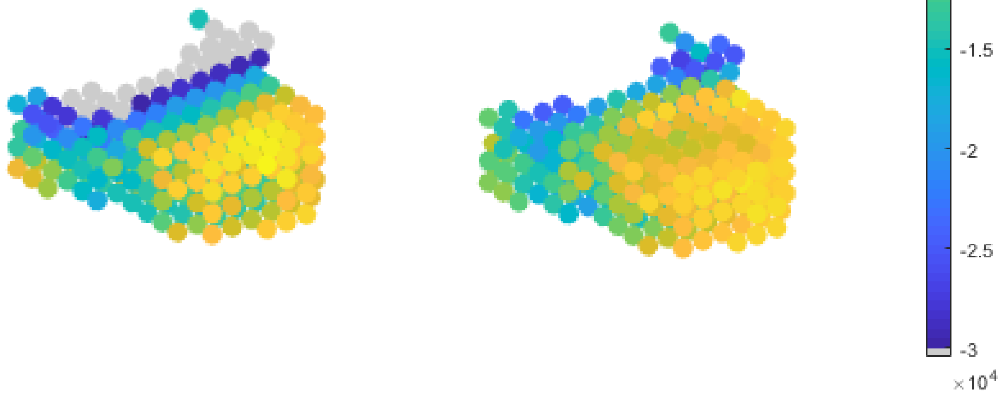

Supplement: Supplementary file 1 — Supplementary file1 (PDF 4678 KB) [file 10237_2025_1950_MOESM1_ESM.pdf]
