# Supplementary material for: Comparing the predictions of CT-based subject-specific finite element models of human metastatic vertebrae with digital volume correlation measurements
Source: Biomech Model Mechanobiol. 2025 Apr 19;24(3):1017–30. doi: 10.1007/s10237-025-01950-x (PMC12162702; doi:10.1007/s10237-025-01950-x)
Supplement: Supplementary file 2 — Supplementary file2 (DOCX 168 KB) [file 10237_2025_1950_MOESM2_ESM.docx]

SUPPLEMENTARY MATERIAL

**Excluded specimen**

Among the analysed vertebrae, one has been excluded from the validation because it showed a strong dependence of the prediction errors on the DVC uncertainties (Fig.1s). The reason was linked to the possibility that the prediction error was dependent on the experimental uncertainty instead of being caused by modelling assumptions. Hence, those results were no further considered in the analysis.

Predictive error was computed as the absolute difference between DVC and FE displacements at each DVC point. Experimental uncertainties on strains (DVC errors Zero-Strain) were computed applying the BoneDVC algorithm to two subsequent scans of an unloaded specimen (zero-strain condition) (Cavazzoni et al. 2023). They were extracted by considering the standard deviation (SDER) of the average of the absolute values of the six DVC-derived strain components.


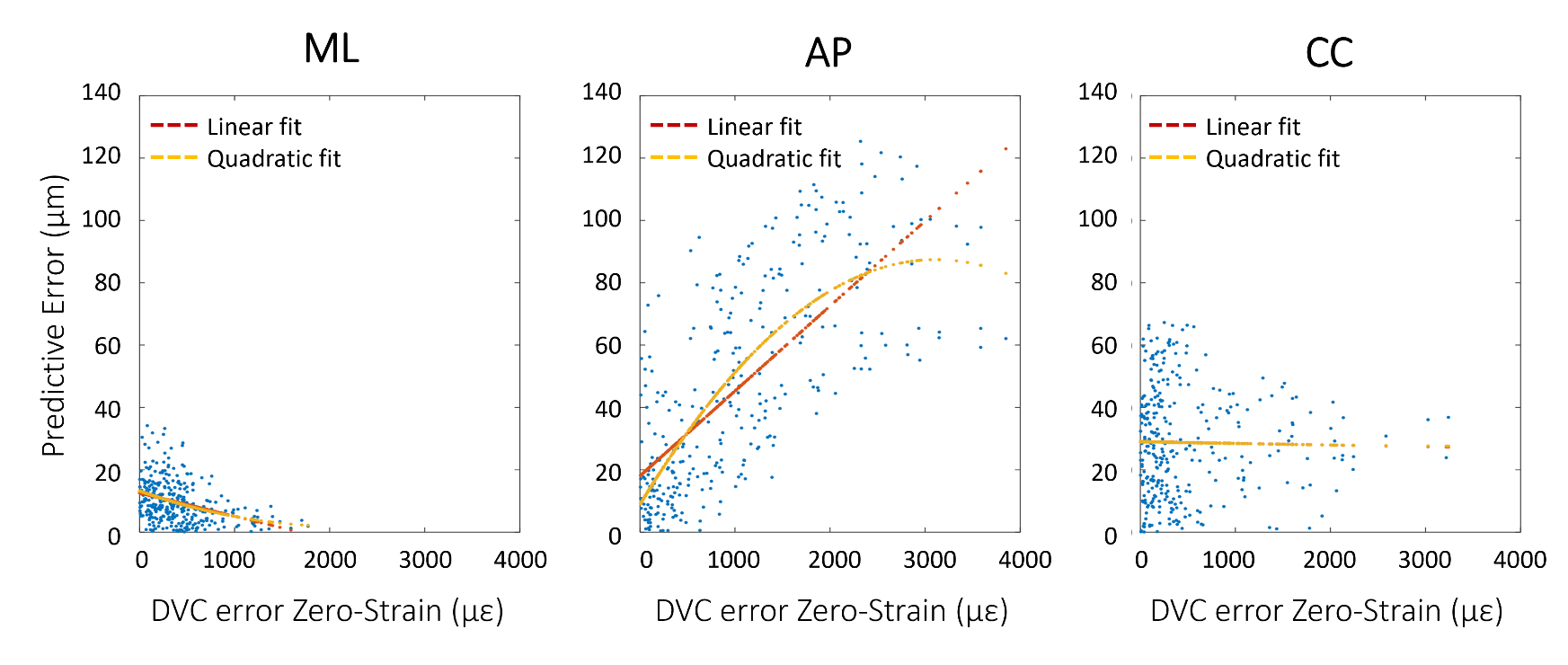


**Fig.1s** Scatter plots between DVC uncertainties (horizontal axis) and predictive errors on displacements (vertical axis), on mediolateral (ML), anteroposterior (AP) and craniocaudal (CC) directions respectively. In the AP direction strong dependence was highlighted.
